# Author Correction: A meta-analysis of genetic effects associated with neurodevelopmental disorders and co-occurring conditions

**DOI:** 10.1038/s41562-025-02299-y

**Published:** 2025-08-11

**Authors:** Agnieszka Gidziela, Yasmin I. Ahmadzadeh, Giorgia Michelini, Andrea G. Allegrini, Jessica Agnew-Blais, Lok Yan Lau, Megan Duret, Francesca Procopio, Emily Daly, Angelica Ronald, Kaili Rimfeld, Margherita Malanchini

**Affiliations:** 1 School of Biological and Behavioural Sciences, Queen Mary University of London, London, UK; 2 Social, Genetic and Developmental Psychiatry Centre, King’s College London, London, UK; 3 UCLA Semel Institute for Neuroscience, Division of Child and Adolescent Psychiatry, University of California, Los Angeles, Los Angeles, CA USA; 4 Division of Psychology and Language Sciences, University College London, London, UK; 5 Department of Psychological Sciences, Birkbeck University of London, London, UK; 6 Department of Psychology, Royal Holloway University of London, Egham, UK

**Keywords:** Behavioural genetics, Human behaviour

Correction to: *Nature Human Behaviour* 10.1038/s41562-023-01530-y. Published online 20 February 2023.

In the originally published version of this study, 8 out of 18 studies addressing the genetic overlap between ADHD and dimensions of specific learning disorders (7 studies addressing reading ability and 1 study addressing mathematic ability) reported negative genetic correlation estimates. Due to a coding artifact, the authors did not reverse direction of these estimates to account for differences in measurement of learning difficulties, as opposed to learning abilities. In the corrected version of the manuscript, accounting for this differential directionality resulted in a greater meta-analytic estimate of the genetic correlation between ADHD and specific learning disorders from 0.07 (s.e. = 0.12) to 0.33 (s.e. = 0.04), which still remained the lowest out of all other genetic correlations among neurodevelopmental disorders. The overall meta-analytic genetic correlation between all categories of neurodevelopmental disorders was also affected: this increased from 0.36 (s.e. = 0.12) to 0.47 (s.e. = 0.06). Differences in these estimates did not substantially impact the interpretation of results and the conclusion. In this corrected version, the estimates of genetic correlation between ADHD and specific learning disorders and across neurodevelopmental disorders have been amended throughout the Abstract, Results and Supplementary Information. Figures 4 and 7 have also been amended. All changes are reflected in the HTML and PDF versions of the article.

