# Supplementary material for: A meta-analysis of genetic effects associated with neurodevelopmental disorders and co-occurring conditions
Source: Nat Hum Behav. 2023 Feb 20;7(4):642–56. doi: 10.1038/s41562-023-01530-y (PMC10129867; doi:10.1038/s41562-023-01530-y)
Supplement: Supplementary file 1 — Supplementary Notes 1–15, Tables 1–50, Figs. 1–30 and References. [file 41562_2023_1530_MOESM1_ESM.pdf]

nature human behaviour

Article https://doi.org/10.1038/s41562-023-01530-y

A meta-analysis of genetic effects associated with neurodevelopmental disorders and co-occurring conditions

In the format provided by the authors and unedited

Table of contents	
**Supplementary Notes**	Page 2
**Supplementary Tables**	Page 28
**Supplementary Figures**	Page 119
**Supplementary References**	Page 151

Supplementary Notes

Supplementary Note 1: Meta-analytic results for shared and nonshared environmental factors. 3
Supplementary Note 2: Meta-analytic results for NDDs phenotypic sub-categories....................................7
Supplementary Note 3: Description of moderators. 9
Supplementary Note 4: Categorical versus continuous measurement of NDDs. 11
Supplementary Note 6: Heterogeneity assessment...14
Supplementary Note 7: Publication bias. 16
Supplementary Note 9: PRISMA 2020 Checklist. 17
Supplementary Note 10: PRISMA 2020 for Abstracts Checklist. 20
Supplementary Note 11: Indexes, timespans, search strategy and key words. 22
Supplementary Note 12: Description of SNP-based methods targeted by the meta-analysis. 24
Supplementary Note 13: Quality scoring checklist. 25
Supplementary Note 14: Requesting missing data from study authors...26
Supplementary Note 15: Aggregation sensitivity analyses. 27

Supplementary Note 1: Meta-analytic results for shared and nonshared environmental factors.

Shared and nonshared environmental influences on NDDs

Shared environment (c^2)

We identified 127 studies that reported information on shared environmental influences on NDDs, only a little over half (53.6%) of all studies that reported on h^2 also reported on c^2. Out of the total 127 studies, 65 studies focused on specific learning disorders, 48 on ADHD, 15 on communication disorders, 14 on ASD, 3 on motor disorders, and 0 studies included c^2 estimates for intellectual disabilities, the only two studies that had examined the aetiology of intellectual disabilities had reported a model only including genetic and nonshared environmental factors (AE) as the best fitting model (see **Methods** and **Supplementary Note 3**). The contribution of shared environmental influences to all NDD categories was modest ($c^2 = 0.17$, SE= 0.02), ranging from weak ($c^2 = 0.10$, SE= 0.02) for ADHD to moderate ($c^2 = 0.36$, SE= 0.06) for communication disorders (**Figure 3** in the main text and **Supplementary Table 1**).

Nonshared environment (e^2)

We identified 195 family-based studies (82.2% of the total) that reported on the nonshared environmental contribution to NDDs, out of which 107 studies focused on ADHD, 67 on specific learning disorders, 28 on ASD, 18 on communication disorders, 6 on motor disorders and 2 studies on intellectual disabilities. Nonshared environmental influences on all NDDs were moderate ($e^2 = 0.29$, SE= 0.02), but ranged from weak ($e^2 = 0.10$, SE= 0.16) for intellectual disabilities to moderate ($e^2 = 0.38$, SE= 0.11) for motor disorders. Nonshared environmental estimates did not differ significantly across all NDDs (**Figure 3** in the main text and **Supplementary Table 1**).

Shared and nonshared environmental overlap between NDDs

Shared environmental correlations (rC)

Since several studies only reported the most parsimonious, best-fitting, model (see **Supplementary Note 3**), meta-analytic estimates of rC could be derived from 16 studies (43.2% of the total number; **Supplementary Table 3**). A first meta-analysis of all NDD categories jointly, yielded a significant and substantial grand estimate for the shared environmental co-occurrence between different NDDs (rC= 0.63, SE= 0.32), although estimates varied substantially between studies, as indicated by the large meta-analytic standard error.

Nonshared environmental correlations (rE)

A total of 22 studies (59.5%) reported on the nonshared environmental co-occurrence between NDDs, this was largely due to the fact that different studies adopted different family-based designs, some of which do not provide nonshared environmental estimates[1] (see **Supplementary Note 3**). The grand estimate for the transdiagnostic rE was 0.17, SE= 0.5. When we considered NDD categories separately, nonshared environmental correlations could only be estimated between ASD & ADHD (5 studies, rE = 0.22, SE= 0.13), and between ADHD & specific learning disorders (9 studies, rE = 0.11, SE= 0.05; **Figure 4** in the main text and **Supplementary Table 3**).

Shared and nonshared environmental overlap between NDDs and DICCs

Shared environmental correlations (rC)

Out of 15 studies that reported genetic correlations between NDDs and DICCs, 11 also reported shared environmental correlations (73.3%). These included 4 studies looking at the co-occurrence between ADHD & oppositional defiant disorder, 3 studies looking at the co-occurrence between ADHD & conduct disorder, and 3 studies looking at the co-occurrence between ASD & conduct disorder. A strong meta-analytic shared environmental correlation was found between all NDDs and DICCs (0.88, SE= 0.34). The grand shared environmental overlap was consistently estimated as very high for all co-occurring disorders for which we identified sufficient studies: rC= 0.96 (SE= 0.57) between ADHD & oppositional defiant disorder, rC= 0.94 (SE= 0.71) between ADHD & conduct disorder, and rC= 0.88 (SE= 0.57) between ASD & conduct disorder (**Figure 4** in the main text and **Supplementary Table 5**).

Nonshared environmental correlations (rE)

Thirteen out of 15 studies that reported on the genetic overlap between NDDs and DICCs also reported nonshared environmental correlations (86.7%). These 13 studies consisted of 5 studies targeting the co-occurrence between ADHD & conduct disorder, 5 studies that between ADHD & oppositional defiant disorder, and 3 studies the co-occurrence between ASD & conduct disorder. The nonshared environmental overlap across all NDD and DICC pairs was moderate (rE = 0.39, SE= 0.14), but differed between specific pairs of disorders. The strongest correlation (rE = 0.54, SE= 0.25) was found between ADHD & oppositional defiant disorder and was markedly higher if compared to the overlap between ADHD & conduct disorder (rE = 0.11, SE= 0.08) and between ASD & conduct disorder (0.07, SE= 0.08) (**Figure 4** in the main text and **Supplementary Table 5**).

Sex differences

Sex differences in environmental aetiology of NDDs

Across all NDDs, family-based shared and nonshared environmental influences were not significantly different between males (c^2= 0.35, SE= 0.09; e^2= 0.31, SE= 0.05) and females (c^2= 0.28, SE= 0.08; e^2= 0.33, SE= 0.04). Distributions of sex-specific family-based variance components for all NDDs, except for motor disorders for which a sufficient number of studies (>1) was not identified, are presented in **Figure 5** in the main text and **Supplementary Table 16)**

Sex differences in environmental overlap between NDDs

Sex-specific shared environmental correlations could not be estimated, whereas nonshared environmental correlations were estimated at 0.09 (SE= 0.08) in males and 0.10 (SE= 0.11) in females (**Supplementary Table 17)**. Sex-specific grand estimates of environmental correlations between specific disorders are not reported because of the limited number of studies identified. The only exception was the co-occurrence between ASD & ADHD in males, where 2 studies were identified (rE = 0.20, SE= 0.14; **Supplementary Table 17)**. Due to the lack of available studies, the shared environmental overlap could not be calculated.

Sex differences in environmental overlap between NDDs and DICCs
We could only meta-analyse the co-occurrence between ADHD & conduct disorder in females. We found a meta-analytic nonshared environmental correlation of 0.06 (SE= 0.12; **Supplementary Table 18)**.

Developmental trends trajectories

Age-related differences in environmental aetiology of NDDs
Across all NDDs, grand shared and nonshared environmental influences were observed to decrease from childhood (c^2= 0.21, SE= 0.04; e^2= 0.27, SE= 0.03) to middle childhood (c^2= 0.12, SE= 0.03; e^2= 0.25, SE= 0.02) followed by a later increase in adolescence (c^2= 0.17, SE= 0.03; e^2= 0.36, SE= 0.03). This trend was consistent across some specific NDDs, such as ASD and ADHD, but not for others. For example, for communication disorders and specific learning disorders genetic and shared environmental variance decreased while nonshared environmental variance increased developmentally (**Figure 6A** in the main text and **Supplementary Table 19**).

Age-related differences in environmental overlap between NDDs, as well as between NDDs and DICCs
Overall, we could not explore developmental trends in genetic and environmental correlations due to a lack of available studies, the only exceptions were grand estimates for adolescence (see **Supplementary Tables 28-30)**.

Categorical versus continuous measurement of NDDs

We found no significant differences in shared and nonshared environmental influences between measurement methods (**Supplementary Figure 22** and **Supplementary Table 25**). Furthermore, shared and nonshared environmental genetic overlap could not be compared across co-occurrences between NDDs, and between NDDs and DICCs, due to insufficient number of identified studies (**Supplementary Figure 22** and **Supplementary Tables 26 and 27**).

Geographical differences

Geographical differences in environmental aetiology of NDDs
Grand shared environmental influences ranged between 0.30 (SE= 0.13) in Chinese cohorts and 0.07 (SE= 0.04) in Swedish cohorts (**Figure 7A** in the main text and **Supplementary Table 19**), whereas nonshared environmental influences were highest in Canada (0.38, SE= 0.07), if compared to the lowest grand estimate of nonshared environmental influence (0.17, SE= 0.05) obtained for Australian cohorts (**Figure 7A** in the main text and **Supplementary Table 22**).

Geographical differences in environmental overlap between NDDs
The highest meta-analytic estimate of shared environmental correlation was estimated in United Kingdom-based samples (0.91, SE= 0.29), while the lowest in United States-based studies (0.07, SE= 0.21; **Figure 7B** in the main text and **Supplementary Table 23**). The strongest grand estimate of nonshared environmental correlation was found in Swedish samples (0.36, SE= 0.12) while the lowest in Australian samples (0.03, SE= 0.09; **Figure 7B** in the main text and **Supplementary Table 23**).

Geographical differences in environmental overlap between NDDs and DICCs
Studies yielded consistently strong estimates of shared environmental correlation across the United Kingdom, United Stated and Sweden (0.97, SE= 0.57; 0.85, SE= 0.56; and 0.89, SE= 0.55; **Figure 7C** in the main text and **Supplementary Table 24**). Grand nonshared environmental correlations could only be calculated for United Kingdom and United States-based studies and were estimated at 0.49 (SE= 0.44) and 0.24 (SE= 0.09), respectively (**Supplementary Figure 28** and **Supplementary Table 24**).

Ancestral differences

Ancestry-related differences in the environmental aetiology of NDDs
Meta-analytic shared environmental influences remained relatively stable across sample ancestral composition (mean of c^2= 0.24) with only a slight drop observed when the sample included 100% of participants of European ancestry (c^2= 0.19, SE= 0.04; **Supplementary Figure 27** and **Supplementary Table 25**). However, estimates differed for specific disorders. The decrease in shared environmental influences in fully European descent samples was especially evident for ADHD, where the estimates dropped from a mean of 0.17 for more diverse categories to 0.04 (SE= 0.09) for 100% European ancestry samples. A similar pattern was observed for specific learning disorders, with estimates dropping from a mean of 0.26 to 0.16 (SE= 0.04) (**Supplementary Figure 27** and **Supplementary Table 25**).

All NDDs were subject to subtle changes in nonshared environmental influences depending on the ancestral composition of the samples, with the exception of motor disorders for which only studies using 100% European ancestry samples were found. Across all NDDs, the meta-analytic estimate for nonshared environmental influences decreased as the percentage of participants of European ancestry in the sample increased: from 0.44 (SE= 0.08) for samples where participants of European ancestry were in the minority, to 0.32 (SE= 0.13) for samples where they were between 50 and 74% to 0.25 (SE =0.03) for samples between 75 and 99% European ancestry) to 0.32 (SE= 0.05) for 100% European ancestry samples. This same trend was observed for ADHD (from 0.54, SE= 0.09 to 0.39, SE= 0.06) and specific learning disorders (0.28, SE= 0.06 to 0.19, SE= 0.06, although the estimate increased again for samples 100% of European descent, 0.30, SE= 0.07; **Supplementary Figure 27** and **Supplementary Table 25**). For communication disorders, e^2 increased from 0.16 (SE= 0.11) for samples 75-99% European ancestry to 0.24 (SE= 0.06) for samples where all participants were of European ancestry.

Ancestry-related differences in environmental overlap between NDDs
Differences in sources of co-occurrence between NDDs could not be estimated for shared and nonshared environmental overlap. Estimates for samples comprising only individuals of European ancestry are presented in **Supplementary Table 26**.

Ancestry-related differences in environmental overlap between NDDs and DICCs
We were able to estimate the meta-analytic shared environmental overlap between NDDs and DICCs, as 4 out of 5 studies reporting on genetic correlations also reported on shared environmental correlations. The grand shared environmental overlap remained stable across samples ancestral composition (0.88, SE= 0.87 and 0.89, SE= 0.85, respectively; **Supplementary Table 27**).

Supplementary Note 2: Meta-analytic results for NDDs phenotypic sub-categories.

Where the number of studies identified was sufficiently large, we were able to stratify sources of variance and co-occurrence by specific phenotypic sub-categories to reflect within-category differences. **Supplementary Figure 2** presents family and SNP-based heritability, shared and nonshared environmental influences on sub-categories of NDDs, whereas **Supplementary Figure 3** shows family-based genetic, shared and nonshared environmental overlap between sub-categories of NDDs, as well as between sub-categories of NDDs and DICCs. All estimates with standard errors are presented in **Supplementary Tables 2-5.**

For example, within intellectual disabilities, we estimated heritability of learning disability (0.86, SE= 0.43), which constitutes one of the sub-categories. Within communication disorders, we distinguished 5 specific phenotypes, out of which specific language impairment had the highest meta-analytic heritability (0.87, SE= 0.60), whereas the lowest grand heritability estimate was estimated for stuttering (0.58, SE= 0.17). All ADHD-related specific phenotypes were highly heritable, ranging from 0.76 (SE= 0.07) for impulsivity to 0.65 (SE= 0.05) for inattention. For ASD, the highest grand heritability was found for restrictive and repetitive behaviours and interests (0.83, SE= 0.49), whereas the lowest was found for social impairments (0.67, SE= 0.05). Within motor disorders, we identified 4 specific sub-categories. The highest grand heritability estimate was found for motor coordination (0.82, SE= 0.08) and the weakest for tic disorders (0.56, SE= 0.17).

Specific learning disorders were divided into three primary sub-categories, i.e., dyslexia, dysgraphia, and dyscalculia-related phenotypes with heritabilities ranging from 0.62 (SE= 0.04) for dyslexia (and/or the continuously measured phenotype of reading difficulty) to 0.56 (SE= 0.18) for dysgraphia (and/or the continuously measured phenotype of writing difficulty), and 0.55 (SE= 0.04) for dyscalculia (and/or the continuously measured phenotype of mathematics difficulty). The three subcategories of dyslexia, dysgraphia, and dyscalculia were further divided into secondary sub-categories comprising specific reading, writing and mathematics-related phenotypes. Within the dyslexia sub-category, the highest meta-analytic heritability was estimated for decoding (0.69, SE= 0.14), while the lowest for vocabulary (0.25, SE= 0.14). Within the dysgraphia-related phenotype, writing difficulty had a grand heritability estimate of 0.56 (SE= 0.17). Within the Dyscalculia sub-category, we identified 4 further specific phenotypes, out of which broadly defined mathematics difficulty was most heritable, with a meta-analytic estimate of 0.57 (SE= 0.04), with the lowest grand heritability obtained for mathematics problem solving (0.36, SE= 0.18).

Stratified estimates for specific phenotypes could also be calculated for a few homotypic and heterotypic co-occurrent disorders. The co-occurrence between ASD & ADHD was divided into 4 sub-categories, out of which the highest meta-analytic genetic correlation was obtained between broadly defined ASD & ADHD (0.71, SE= 0.27), while the lowest was estimated between restrictive and repetitive behaviours and interests & inattention (0.16, SE= 0.11; see **Supplementary Table 4**).
We could only distinguish only one specific phenotype sub-category for the co-occurrence between ADHD & motor disorders, namely the association between ADHD & developmental coordination disorder for which grand genetic correlation of 0.91 (SE= 0.80) was found. The co-occurrence between ADHD & specific learning disorders was stratified into 6 phenotypic sub-categories, with the overlap ranging between

0.39 (SE= 0.08) for inattention & reading difficulty and 0.21 (SE= 0.06) for hyperactivity & reading difficulty. The co-occurrence between specific language impairment and dyslexia was the only specific phenotype sub-category identified for the co-occurrence between communication disorders & specific learning disorders and yielded grand genetic overlap of 0.66 (0.15), whereas the co-occurrence between subtypes of specific learning disorders was stratified into dyslexia and dyscalculia and quantitatively measured reading difficulty and mathematics difficulty, both of which yielded comparable meta-analytic genetic overlaps: 0.56 (SE= 0.07) and 0.55 (SE= 0.08), respectively.

When considering the genetic overlap between NDDs and DICCs, stratification was only possible for the co-occurrence between ADHD & oppositional defiant disorder, where the grand genetic overlap between hyperactivity & oppositional defiant disorder traits was stronger (0.80, SE=0.57) if compared to the genetic overlap between inattention & oppositional defiant disorder traits (0.52, SE= 0.10).

Supplementary Note 3: Description of moderators.

Age

The age group moderator was created based on age range of the study, or the mean age when the age range was not reported, and consisted of six levels, three separate categories and three groups cutting across age categories: childhood (ages 4-7), middle childhood (ages 8-10), adolescence (ages 11-24), childhood & middle childhood (ages 4-10), middle childhood & adolescence (ages 8-24) and childhood & adolescence (ages 4-24). The same age categories were used across all methods.

Design

The design covariate consisted of different categories, depending on whether the study had employed family or SNP-based methods. For family-based studies, 8 types of designs were identified: classical twin study, categorical threshold twin study, DFextremes twin study, classical twin and sibling study, categorical threshold twin and sibling study, DFextremes twin and sibling study, classical sibling study and categorical threshold sibling study. We identified two types of designs for SNP-based studies: those using genome-wide (GREML) and summary-level data (LDSC).

Model

When meta-analysing family-based studies we also controlled for type of model, i.e., full model (twin or twin and sibling studies reporting A, C and E estimates), DFextremes full model (DFextremes studies reporting A, C and E estimates), best model (twin or twin and sibling studies reporting best-fitting parsimonious models, that is either AE, CE or E only models), DFextremes best model (DFextremes studies reporting best-fitting parsimonious models, that is either AE, CE or E only models), A only model (twin or twin and sibling studies reporting heritability estimates only, without providing estimates of C and E), DFextremes A only model (DFextremes studies reporting heritability estimates only, without providing estimates of C and E).

Rater

Eight types of raters were identified with the meta-analytic dataset, referring to both family and SNP-based studies. NDD and DICC symptoms were rated by either parents, teachers, self-reports, or researchers, with several studies reporting cross-rater measures assessed by parents & teachers and parents & self-reports. In addition, specific learning disorders and communication disorders symptoms were often assessed using reading, writing, mathematical and language ability tests, hence test was also included as an additional level of this covariate. A further level, diagnosis, was also incorporated to reflect clinical diagnosis of NDDs and DICCs.

Measurement scale

Measurement scale moderator involved two levels, continuous reflecting quantitatively measured symptoms and categorical reflecting binary diagnoses and clinical cut-offs.

Ancestry

From studies that reported on the ancestral composition of the sample used in analyses we recorded the percentage of participants of European ancestry. We created the %European ancestry and created a moderator with four levels: less than 50%, more than 50% but less than 75%, more than 75% but less than 100% and 100%.

Number of covariates

Behaviour genetic studies often include covariates in the models or regress covariates out prior to analyses. It is a common procedure to control for age and sex in both family and SNP-based studies, and additionally controlling for batch effects and population stratification in molecular genetics studies[2,3]. To determine the impact of including covariates on estimate heterogeneity, we created a moderator by adding up the number of covariates used in each study. This resulted in a moderator including 5 levels: 0 to 4 covariates included.

Measure

Further heterogeneity between studies may arise from differences in the measurement instruments used to assess NDDs and DICCs. Diagnostic and assessment tools tend to be specific to the disorder being measured, therefore we created a moderator variable indexing the specific measurement instrument used to assess each NDD category, with levels varying within and between conditions.

Country

The last moderator involved the country where each cohort was based. We distinguished eight levels of this moderator: Australia, Canada, China, Netherlands, Norway, Sweden, United Kingdom, and United States.

Supplementary Note 4: Categorical versus continuous measurement of NDDs.

Family-based studies

Categorical phenotypes were measured by 28 family-based studies, whereas 215 studies reported estimates for continuous phenotypes. Higher grand heritability was estimated for categorically measured NDDs (0.77, SE= 0.07), compared to NDDs measured on a continuum (0.64, SE= 0.03) (**Supplementary Figure 26; Supplementary Table 28**). No significant differences in shared and nonshared environmental influences were present between measurement methods.

Disparities in family-based genetic overlap was found across co-occurrences between NDDs, with grand genetic correlation of 0.56 (SE= 0.32) estimated from studies using categorical phenotypes and 0.47 (SE= 0.06) estimated from studies using quantitative measures (**Supplementary Figure 26** and **Supplementary Table 29**). Shared and nonshared environmental genetic overlap could not be compared across co-occurrences between NDDs due to insufficient number of identified studies. Similarly, sources of co-occurrence could not be compared between measurement scales for the co-occurrence between NDDs and DICCs as less than 2 studies investigated categorically defined phenotypes (**Supplementary Figure 26** and **Supplementary Table 30**).

SNP-based studies

Categorically and quantitatively defined NDDs were measured by 12 and 17 SNP-based studies, respectively. Just as family-based heritability, SNP heritability across NDDs differed between measures: categorical phenotypes yielded lower heritability (0.17, SE= 0.03) estimates if compared to quantitatively measured symptom scores (0.25, SE= 0.06; **Supplementary Figure 26** and **Supplementary Table 28**).

Supplementary Note 5: Meta-analytic results for different levels of sample diversity.

Family-based heritability (h^2)

Given the general lack of diversity in participants' ancestry, we could only examine this issue by calculating how samples differed between each other in terms of their percentage of participants of European ancestry. A related issue was also that less than half of the studies reported information on the ancestral composition of their sample (97 out of the 236 studies).

Across all NDDs, heritability was observed to increase with increasing percentage of participants of European ancestry, from 0.46 (SE= 0.07) when they constituted less than half of the sample to 0.66 (SE= 0.06) when 100% of the sample was of European ancestry (**Supplementary figure 27; Supplementary Table 25**). This trend was particularly observed for ADHD, where the heritability increased from 0.41 (SE= 0.12) in samples where European ancestry participants were the minority (less than 50%) to 0.67 (SE= 0.04) in samples where European ancestry participants were the totality. On the other hand, genetic influences on communication disorders and specific learning disorders remained stable across ancestral compositions: For communication disorders, heritability estimates ranged between 0.59 (SE= 0.27) in samples less than 75-99% of European ancestry to 0.56 (SE= 0.09) in samples 100% of European descent. For Specific learning disorders, heritability was 0.54 (SE= 0.16) in samples where European ancestry participants were in the minority vs. 0.61 (SE= 0.04) in samples 100% of European ancestry.

SNP heritability (SNP h^2)

We did not identify SNP-based studies that used samples other than 100% European ancestry in populations of children and adolescents.

Ancestry-related differences in genetic overlap between NDDs

Differences in sources of co-occurrence between NDDs could only be estimated for the genetic overlap between all NDDs, where a total of 6 studies were identified. Two studies (one focusing on the co-occurrence between ADHD & specific learning disorders, and the other on the co-occurrence between subtypes of specific learning disorders) reported estimates for sample where participants were between 75% and 99% of European ancestry, while 4 studies (2 on the co-occurrence between ADHD & specific learning disorders, and 2 on the co-occurrence between subtypes of specific learning disorders) included samples where 100% of the participants were of European descent. The meta-analytic genetic overlap between NDDs decreased, albeit not significantly, from 0.63 (SE= 0.44) in samples where 75-99% of European ancestry to 0.54 (SE= 0.10) in samples entirely of European ancestry (**Supplementary Table 26)**.

SNP-based studies (6 in total) addressing the co-occurrence between NDDs were exclusively conducted in combined samples from the United Kingdom and Denmark (**Supplementary Table 26**).

Ancestry-related differences in genetic overlap between NDDs and DICCs

Estimating the sources of co-occurrence between NDDs and DICCs by percentage of sample diversity was similarly challenging as we could identify only 5 studies that included the relevant information. Out of the total number of studies, 3 involved samples of between 75% and 99% participants of European ancestry and focused on examining the genetic overlap between ADHD & conduct disorder and ADHD & oppositional defiant disorder, while 2 involved samples of 100% European descent and examined the genetic correlations

between ADHD & oppositional defiant disorder and ADHD & disruptive behaviour. The meta-analytic genetic overlap between NDDs and DICCs increased, albeit not significantly, from 0.57 (SE= 0.25) in samples involving less than 100% of European ancestry participants to 0.71 (SE= 0.31) in 100% European ancestry samples (**Supplementary Table 27)**.

Supplementary Note 6: Heterogeneity assessment.

Across all NDDs we found that 74% of the total variance in family-based heritability was due to heterogeneity, out of which 53% could be attributed to between-cluster and 22% to within-cluster heterogeneity, where clusters refer to cohorts and individual studies (**Supplementary figure 4; Supplementary table 7**). The lowest I^2 statistic was estimated for motor disorders (36%, with equal contribution of between and within-cluster heterogeneity of 18% each), while the highest one for ASD (86%, where 78% was attributed to between-cluster and 8% to within-cluster heterogeneity). When considering SNP heritability, the proportion of total variance accounted for by heterogeneity was very low across disorders (6-8%, most of which was represented by between-cluster heterogeneity). Total variance in shared environmental influences across NDDs was moderate (18%) and almost exclusively attributable to within-cluster heterogeneity. The highest proportion of variance in shared environmental influences accounted for by heterogeneity was found for ASD (41%) and was accounted for solely by within-cluster heterogeneity, while the lowest was found for specific learning disorders and motor disorders, for which variance explained by heterogeneity was less than 0.001%. A similar degree of heterogeneity was estimated for nonshared environmental factors, where the variance explained across NDDs was 38% (21% and 17% attributed to between and within-cluster heterogeneity, respectively) and ranged from 43% (accounted solely by within-cluster heterogeneity) for ADHD to less than 0.001% for intellectual disabilities.

Overall, heterogeneity in genetic correlations between NDDs were estimated as 75%, entirely attributed to within-cluster heterogeneity (**Supplementary figure 4; Supplementary table 8**). The largest proportion of total variance accounted for by heterogeneity was estimated for the co-occurrence between ADHD & motor disorders (99%, with equal contribution of between and within-cluster heterogeneity of 49%), whereas the lowest one was estimated for the co-occurrence between communication disorders & motor disorders, communication disorders & specific learning disorders and ADHD & specific learning disorders (<0.001% each). Heterogeneity in SNP-based genetic overlap across co-occurrences between NDDs accounted for 49% of the total variance, with 33% attributed to between-cluster and 15% to within-cluster heterogeneity. Between ASD & ADHD, 24% of the total variance was explained by heterogeneity, all of which was accounted for by between-cluster heterogeneity.

Variance in shared environmental overlap across co-occurrences between NDDs accounted for by heterogeneity was estimated as 95%, with 36% attributed to between-cluster and 59% to within-cluster heterogeneity and for the only pair of NDDs where meta-analysis of shared environmental correlations was possible, i.e., ADHD & specific learning disorders, we found 53% of the total variance to be explained by heterogeneity with 6% attributed to between-cluster and 47% to within-cluster heterogeneity. Variance in nonshared environmental overlap across NDDs was modest (24%, all accounted for by between-cluster heterogeneity) and ranged from 62% (all accounted for by between-cluster heterogeneity) for the co-occurrence between ASD & ADHD to less than 0.001% for the co-occurrence between ADHD & specific learning disorders.

Finally, 93% of the total variance in genetic overlap across co-occurrences between NDDs and DICCs was accounted for by heterogeneity, with 55% attributed to between-cluster and 38% to within-cluster heterogeneity (**Supplementary Figure 4** and **Supplementary Table 9**). The variance explained by heterogeneity was high for co-occurrence between ADHD & conduct disorder (92%, with equal contribution

of between and within-cluster heterogeneity, 46% each) and between ADHD & oppositional defiant disorder (84%, with equal contribution of between and within-cluster heterogeneity, 42% each), but much lower between ASD & conduct disorder (less than 0.001%). In case of shared environmental overlap between NDDs and DICCs, 95% of the variance was due to heterogeneity and was solely accounted for by within-cluster heterogeneity. The highest proportion of variance in shared environmental correlations explained by heterogeneity was estimated for co-occurrence between ADHD & conduct disorder (96%, with equal contribution of between and within-cluster heterogeneity, 48% each), whereas the lowest was estimated between ASD & conduct disorder (67%, all accounted for by within-cluster heterogeneity). Total variance in nonshared environmental overlap was high across all co-occurrences between NDDs and DICCs (91%, all accounted for by within-cluster heterogeneity), as well as between ADHD & oppositional defiant disorder (92%, equally accounted for by between and within-cluster heterogeneity, 46% each), whereas less than 0.001% of variance in nonshared environmental overlap between ADHD & conduct disorder and ASD & conduct disorder was explained by heterogeneity.

Supplementary Note 7: Publication bias.

Publication bias refers to the higher probability of studies reporting statistically significant findings being accepted for publication. In an unbiased scenario, we would expect to find as many studies reporting significant results, as those not rejecting the null hypothesis. The publication bias can be reflected by the linear relationship between the estimate and standard error[4]. **Supplementary Figures 8-14** include funnel plots of studies that reported estimates of heritability, shared and nonshared environmental influences on NDDs. **Supplementary Table 13** presents the results of Egger's regressions for all NDDs, apart from intellectual disabilities where the number of parameters to be estimated was larger than the number of studies. A significant risk of publication bias ($z= -3.95$, $beta= 0.73$ (95% CIs: 0.69, .78), $p< 0.001$) for family-based heritability was found across all NDDs, largely driven by ADHD and specific learning disorders. The overall relationship between shared environmental influences and their standard errors was significant across all NDDs, suggesting the greater likelihood of reporting significant estimates in larger studies. This relationship was not significant for specific NDDs. Publication bias was also found for nonshared environmental influences across all NDDs, which was likely driven by nonshared environmental influences on ADHD. Risk of publication bias was not observed for SNP heritability.

Supplementary Figures 15-20 include funnel plots of studies that reported estimates of genetic, shared and nonshared environmental overlap between NDDs. **Supplementary Table 14** presents the results of Egger's regressions across all comorbidities between NDDs, as well as for comorbidities between ASD & ADHD and ADHD & specific learning disorders. For the remaining comorbidities between NDDs the number of parameters to be estimated was larger than the number of studies identified. Risk of publication bias was not significant for family-based genetic and environmental correlations nor for SNP-based genetic correlations.

Supplementary Figures 21-24 include funnel plots of studies that reported estimates for the genetic, shared and nonshared environmental overlap between NDDs and DICCs. **Supplementary Table 15** presents the results of Egger's regressions across all comorbidities between NDDs and DICCs, as well as for comorbidities between ADHD & conduct disorder and ADHD & oppositional defiant disorder and ASD & antisocial personality disorder. We found a significant relationship between environmental influences and standard errors, i.e., publication bias, for shared environmental correlation between all NDDs and all DICCs, and, when considering specific disorder categories, between ADHD & conduct disorder.

Supplementary Note 9: PRISMA 2020 Checklist.

PRISMA 2020 Checklist

Section and Topic	Item #	Checklist item	Location where item is reported
**TITLE**			
Title	1	Identify the report as a systematic review.	Title
**ABSTRACT**			
Abstract	2	See the PRISMA 2020 for Abstracts checklist.	Supplementary Material
**INTRODUCTION**			
Rationale	3	Describe the rationale for the review in the context of existing knowledge.	Introduction
Objectives	4	Provide an explicit statement of the objective(s) or question(s) the review addresses.	Introduction
**METHODS**			
Eligibility criteria	5	Specify the inclusion and exclusion criteria for the review and how studies were grouped for the syntheses.	Methods
Information sources	6	Specify all databases, registers, websites, organisations, reference lists and other sources searched or consulted to identify studies. Specify the date when each source was last searched or consulted.	Methods
Search strategy	7	Present the full search strategies for all databases, registers and websites, including any filters and limits used.	Methods
Selection process	8	Specify the methods used to decide whether a study met the inclusion criteria of the review, including how many reviewers screened each record and each report retrieved, whether they worked independently, and if applicable, details of automation tools used in the process.	Methods
Data collection process	9	Specify the methods used to collect data from reports, including how many reviewers collected data from each report, whether they worked independently, any processes for obtaining or confirming data from study investigators, and if applicable, details of automation tools used in the process.	Methods
Data items	10a	List and define all outcomes for which data were sought. Specify whether all results that were compatible with each outcome domain in each study were sought (e.g. for all measures, time points, analyses), and if not, the methods used to decide which results to collect.	Methods
	10b	List and define all other variables for which data were sought (e.g. participant and intervention characteristics, funding sources). Describe any assumptions made about any missing or unclear information.	Methods

Study risk of bias assessment	11	Specify the methods used to assess risk of bias in the included studies, including details of the tool(s) used, how many reviewers assessed each study and whether they worked independently, and if applicable, details of automation tools used in the process.	Methods
Effect measures	12	Specify for each outcome the effect measure(s) (e.g. risk ratio, mean difference) used in the synthesis or presentation of results.	Methods
Synthesis methods	13a	Describe the processes used to decide which studies were eligible for each synthesis (e.g. tabulating the study intervention characteristics and comparing against the planned groups for each synthesis (item #5)).	Methods
	13b	Describe any methods required to prepare the data for presentation or synthesis, such as handling of missing summary statistics, or data conversions.	Methods
	13c	Describe any methods used to tabulate or visually display results of individual studies and syntheses.	Methods
	13d	Describe any methods used to synthesize results and provide a rationale for the choice(s). If meta-analysis was performed, describe the model(s), method(s) to identify the presence and extent of statistical heterogeneity, and software package(s) used.	Methods
	13e	Describe any methods used to explore possible causes of heterogeneity among study results (e.g. subgroup analysis, meta-regression).	Methods
	13f	Describe any sensitivity analyses conducted to assess robustness of the synthesized results.	Methods
Reporting bias assessment	14	Describe any methods used to assess risk of bias due to missing results in a synthesis (arising from reporting biases).	Methods
Certainty assessment	15	Describe any methods used to assess certainty (or confidence) in the body of evidence for an outcome.	Methods
**RESULTS**			
Study selection	16a	Describe the results of the search and selection process, from the number of records identified in the search to the number of studies included in the review, ideally using a flow diagram.	Results
	16b	Cite studies that might appear to meet the inclusion criteria, but which were excluded, and explain why they were excluded.	Results
Study characteristics	17	Cite each included study and present its characteristics.	Supplementary Material
Risk of bias in studies	18	Present assessments of risk of bias for each included study.	Supplementary Material
Results of individual studies	19	For all outcomes, present, for each study: (a) summary statistics for each group (where appropriate) and (b) an effect estimate and its precision (e.g. confidence/credible interval), ideally using structured tables or plots.	Results
Results of syntheses	20a	For each synthesis, briefly summarise the characteristics and risk of bias among contributing studies.	Results & Supplementary Material
	20b	Present results of all statistical syntheses conducted. If meta-analysis was done, present for each the summary estimate and its precision (e.g. confidence/credible interval) and measures of statistical heterogeneity. If comparing groups, describe the direction of the effect.	Results

	20c	Present results of all investigations of possible causes of heterogeneity among study results.	Supplementary Material
	20d	Present results of all sensitivity analyses conducted to assess the robustness of the synthesized results.	Supplementary Material
Reporting biases	21	Present assessments of risk of bias due to missing results (arising from reporting biases) for each synthesis assessed.	Supplementary Material
Certainty of evidence	22	Present assessments of certainty (or confidence) in the body of evidence for each outcome assessed.	Methods
**DISCUSSION**			
Discussion	23a	Provide a general interpretation of the results in the context of other evidence.	Discussion
	23b	Discuss any limitations of the evidence included in the review.	Discussion
	23c	Discuss any limitations of the review processes used.	Methods
	23d	Discuss implications of the results for practice, policy, and future research.	Discussion
**OTHER INFORMATION**			
Registration and protocol	24a	Provide registration information for the review, including register name and registration number, or state that the review was not registered.	Methods
	24b	Indicate where the review protocol can be accessed, or state that a protocol was not prepared.	Methods
	24c	Describe and explain any amendments to information provided at registration or in the protocol.	Methods
Support	25	Describe sources of financial or non-financial support for the review, and the role of the funders or sponsors in the review.	Acknowledgements
Competing interests	26	Declare any competing interests of review authors.	Competing interests
Availability of data, code and other materials	27	Report which of the following are publicly available and where they can be found: template data collection forms; data extracted from included studies; data used for all analyses; analytic code; any other materials used in the review.	Methods

From: Page MJ, McKenzie JE, Bossuyt PM, Boutron I, Hoffmann TC, Mulrow CD, et al. The PRISMA 2020 statement: an updated guideline for reporting systematic reviews. BMJ 2021;372:n71. doi: 10.1136/bmj.n71

For more information, visit: http://www.prisma-statement.org/

Supplementary Note 10: PRISMA 2020 for Abstracts Checklist.

PRISMA 2020 for Abstracts Checklist

Section and Topic	Item #	Checklist item	Reported (Yes/No)
**TITLE**			
Title	1	Identify the report as a systematic review.	Yes
**BACKGROUND**			
Objectives	2	Provide an explicit statement of the main objective(s) or question(s) the review addresses.	Yes
**METHODS**			
Eligibility criteria	3	Specify the inclusion and exclusion criteria for the review.	Yes
Information sources	4	Specify the information sources (e.g. databases, registers) used to identify studies and the date when each was last searched.	Yes
Risk of bias	5	Specify the methods used to assess risk of bias in the included studies.	Yes
Synthesis of results	6	Specify the methods used to present and synthesise results.	Yes
**RESULTS**			
Included studies	7	Give the total number of included studies and participants and summarise relevant characteristics of studies.	Yes
Synthesis of results	8	Present results for main outcomes, preferably indicating the number of included studies and participants for each. If meta-analysis was done, report the summary estimate and confidence/credible interval. If comparing groups, indicate the direction of the effect (i.e. which group is favoured).	Yes
**DISCUSSION**			
Limitations of evidence	9	Provide a brief summary of the limitations of the evidence included in the review (e.g. study risk of bias, inconsistency and imprecision).	Yes
Interpretation	10	Provide a general interpretation of the results and important implications.	Yes
**OTHER**			
Funding	11	Specify the primary source of funding for the review.	No

Registration	12	Provide the register name and registration number.	No

From: Page MJ, McKenzie JE, Bossuyt PM, Boutron I, Hoffmann TC, Mulrow CD, et al. The PRISMA 2020 statement: an updated guideline for reporting systematic reviews. BMJ 2021;372:n71. doi: 10.1136/bmj.n71

For more information, visit: http://www.prisma-statement.org/

Supplementary Note 11: Indexes, timespans, search strategy and key words.

Searches were conducted with the aid of Covidence (https://www.covidence.org/) and using the following sources:

1) Web of Science.
Core Collection Indexes and timespans:
•Science Citation Index Expanded (SCI-Expanded) -- 1900-present
•Social Sciences Citation Index (SSCI) -- 1900-present
•Arts & Humanities Citation Index (A&HCI) -- 1975-present
•Emerging Sources Citation Index (ESCI) -- 2015-present
•Conference Proceedings Citation Index - Science (CPCI-S) -- 1990-present •Conference Proceedings Citation Index - Social Sciences & Humanities (CPCI SSH) -- 1990-present

2) Ovid platform.
Indexes and timespans:
• Embase (1974 - present)
• Ovid MEDLINE(R), including Epub Ahead of Print and In-Process & Other Non- Indexed Citations (1946 - present)
• LWW Health Library: Speech, Language & Hearing Collection
• Global Health (1973 - present)
• PsycINFO (1806 - present)

To identify studies focusing on the phenotypes of interest, we used the following key terms in the first (primary) search:

((heritab* OR genetic* OR twin* OR genom* OR sibling*) AND (Neurodevelopmental OR "Intellectual* Disabilit*" OR "Learning* Disabilit*" OR "Intellectual* Developmental* Disorder*" OR "Global* Developmental* Delay" OR "Communication Disorder*" OR "Language Disorder*" OR "Speech* Sound* Disorder*" OR "Childhood-Onset* Fluency* Disorder*" OR Stutter* OR "Social Communication Disorder*" OR "Pragmatic Communication Disorder*" OR Autis* OR ASD OR "Attention-Deficit*" OR Hyperactiv* OR Hyperkinetic OR Inattent* OR ADHD OR "Specific Learning Disorder*" OR SLD OR Dyslex* OR Dysgraph* OR Dyscalcul* OR "Motor Disorder*" OR "Developmental Coordination Disorder*" OR Dysprax* OR "Stereotypic Movement Disorder*" OR "Tic* Disorder*" OR "Tourett* Disorder*" OR Disruptive OR "Impulse control" OR "Oppositional Defiant Disorder*" OR ODD OR "Intermittent* Explosive* Disorder*" OR "Conduct* disorder" OR Antisocial* OR APD OR Pyromani* OR Kleptomani* OR "behavio* problem*" OR Deliquen* OR Externalizing))

In the second (confirmatory) search, we decided to include an additional set of terms to capture studies focusing on Specific Learning Disorder and Communication Disorder measured on a continuum (i.e., reading, mathematics, writing, language) that had not been identified by the diagnosis-related search terms (i.e., dyslexia, dyscalculia, dysgraphia, language disorder). The following confirmatory search terms were used:

((heritab* OR genetic* OR twin* OR genom* OR sibling*) AND (Neurodevelopmental OR “Intellectual* Disabilit*” OR “Learning* Disabilit*” OR “Intellectual* Developmental* Disorder*” OR “Global* Developmental* Delay” OR “Communication Disorder*” OR “Language Disorder*” OR “Speech* Sound* Disorder*” OR “Childhood-Onset* Fluency* Disorder*” OR Stutter* OR “Social Communication Disorder*” OR “Pragmatic Communication Disorder*” OR Autis* OR ASD OR “Attention-Deficit*” OR Hyperactiv* OR Hyperkinetic OR Inattent* OR ADHD OR “Specific Learning Disorder*” OR SLD OR Dyslex* OR Dysgraph* OR Dyscalcul* OR Reading OR Math* OR Writing OR Language OR “Motor Disorder*” OR “Developmental Coordination Disorder*” OR Dysprax* OR “Stereotypic Movement Disorder*” OR “Tic* Disorder*” OR “Tourett* Disorder*” OR Disruptive OR “Impulse control” OR “Oppositional Defiant Disorder*” OR ODD OR “Intermittent* Explosive* Disorder*” OR “Conduct* disorder” OR Antisocial* OR APD OR Pyromani* OR Kleptomani* OR “behavio* problem*” OR Deliquen* OR Externalizing))

Supplementary Note 12: Description of SNP-based methods targeted by the meta-analysis.

Genome-wide complex trait analysis and restricted maximum likelihood (GCTA; REML)
The genome-wide complex trait analysis (GCTA) software employs restricted maximum likelihood method (REML) that allows for the estimation of the variance in a trait that is captured by single nucleotide polymorphisms (SNPs) assessed on SNP arrays commonly used in GWAS[5]. This method estimates SNP heritability from DNA in unrelated individuals. The first step is to calculate a genetic relatedness matrix by weighting genetic similarities between all possible pairs of individuals by the allele frequencies across all SNPs on the SNP array. The matrix of pair-by-pair genetic similarity is compared to the matrix of pair-by-pair phenotypic similarity using residual maximum likelihood estimation to obtain the proportion of phenotypic variation accounted for by genetic variation. GCTA can also be used to quantify the degree of shared genetic variance (genetic covariance) between two phenotypes, two disorders for example[5].

Linkage disequilibrium score regression (LDSC)
LDSC quantifies the proportion of variance in a trait explained by common genetic variants (i.e., SNP heritability), as well as the proportion of shared genetic variance between traits (i.e., genetic covariance), using GWAS summary statistics[6]. LDSC applies regression to calculate the association between SNP test statistics obtained from GWAS results, and linkage disequilibrium (LD) scores, therefore allowing us to dissect the true polygenic signal (i.e., the contribution of multiple genetic variants of small effect to variability in a trait or disorder) from confounding signal, including for example false positive associations due to population stratification[6].

Summary-data-based BayesS (SBayeS)
SBayeS is a Bayesian approach to estimating SNP heritability using GWA summary statistics[7]. SBayeS employs an array of linear mixed models using GWA data to estimate SNP heritability, as well as polygenicity and the relationship between variant effect sizes and minor allele frequencies[7].

Supplementary Note 13: Quality scoring checklist.

Quality scoring of the studies included in the present meta-analysis was conducted in line with the framework proposed by Kmet, Cook and Lee (2004)[8].Namely, we used the following checklist:

1. Question/objective sufficiently described?
2. Study design evident and appropriate?
3. Method of subject/comparison group selection or source of information/input variables described and appropriate?
4. Subject (and comparison group, if applicable) characteristics sufficiently described?
5. Outcome and (if applicable) exposure measure(s) well defined and robust to measurement / misclassification bias? Means of assessment reported?
6. Analytic methods described/justified and appropriate?
7. Some estimate of variance is reported for the main results?
8. Results reported in sufficient detail?
9. Conclusions supported by the results?

Items were scored based on the scale developed by Kmet et. al. (2004)[8], where: 0= NO, 1= PARTIAL and 2= YES. Quality scoring was conducted by a primary reviewer and checked by a secondary reviewer. Following completion of the checklist, we calculated the mean total score obtained by each reviewer to ensure inter-rater agreement. Reviewer discrepancies were identified and resolved through discussion.

Supplementary Figure 25 shows our findings for the first 82 studies that were extracted (27.7% of the total). 93.8% of studies showed a low risk of bias across all 9 quality checklist items, and the remaining 6.2% showed moderate risk. Therefore, given the generally low bias, we did not repeat the analyses excluding low-quality studies.

Supplementary Note 14: Requesting missing data from study authors.

The first author of Partitioning the heritability of Tourette syndrome and obsessive compulsive disorder reveals differences in genetic architecture[9] was contacted via e-mail about the age range of the sample. Response was received that the age range of the sample was not restricted and consisted of both children and adults. Therefore, the study was not included in the meta-analysis.

We contacted authors of two other studies via ResearchGate, however we did not receive a response.

Supplementary Note 15: Aggregation sensitivity analyses.

We explored multiple aggregation techniques, that is aggregating non-independent effect sizes by study, by cohort, as well as by country. Furthermore, we checked whether estimates differed when setting different correlation thresholds (r= 0.3, r= 0.5 and r= 0.9) for aggregating between effect sizes. Grand estimates across all NDDs and co-occurring disorders resulting from various aggregation methods are presented in **Supplementary Figure 30**. Grand estimates were not significantly different across aggregation methods and correlation thresholds, therefore we proceeded with aggregating by study and set a fixed correlation between related effect sizes of r= 0.5 for all downstream analyses.

Supplementary Tables

Supplementary Table 1. Heritability, shared and nonshared environmental influences on NDDs. 31
Supplementary Table 2. Heritability, shared and nonshared environmental influences on NDDs, stratified by specific phenotypic sub-categories. 32
Supplementary Table 3. Genetic, shared and nonshared environmental correlations between NDDs. 34
Supplementary Table 4. Genetic, shared and nonshared environmental correlations between NDDs, stratified by specific phenotypic sub-categories. 35
Supplementary Table 5. Genetic, shared and nonshared environmental correlations between NDDs and DICCs. 36
Supplementary Table 6. Genetic, shared and nonshared environmental correlations between NDDs and DICCs, stratified by specific phenotypic sub-categories. 37
Supplementary Table 7. Proportion of variance in heritability, shared and nonshared environmental influences on NDDs accounted for by heterogeneity. 38
Supplementary Table 8. Proportion of variance in genetic, shared and nonshared environmental correlations between NDDs accounted for by heterogeneity. 39
Supplementary Table 9. Proportion of variance in genetic, shared and nonshared environmental correlations between NDDs and DICCs accounted for by heterogeneity. 40
Supplementary Table 10. Proportion of variance in heritability, shared and nonshared environmental influences on NDDs accounted for by heterogeneity, following exclusion of studies identified as outliers. 41
Supplementary Table 11. Proportion of variance in genetic, shared and nonshared environmental correlations between NDDs accounted for by heterogeneity, following exclusion of studies identified as outliers. 42
Supplementary Table 12. Proportion of variance in genetic, shared and nonshared environmental correlations between NDDs and DICCs accounted for by heterogeneity, following exclusion of studies identified as outliers. 43
Supplementary Table 13. Results of Egger's regression for studies addressing heritability and environmental influences on NDDs. 44
Supplementary Table 14. Results of Egger's regression for studies addressing genetic and environmental overlap between NDDs. 45
Supplementary Table 15. Results of Egger's regression for studies addressing genetic and environmental overlap between NDDs and DICCs. 46
Supplementary Table 16. Sex-specific heritability, shared and nonshared environmental influences on NDDs. 47
Supplementary Table 17. Sex-specific genetic, shared and nonshared environmental correlations between NDDs. 48
Supplementary Table 18. Sex-specific genetic, shared and nonshared environmental correlations between NDDs and DICCs. 49
Supplementary Table 19. Heritability, shared and nonshared environmental influences on NDDs, stratified by age categories. 50
Supplementary Table 20. Genetic, shared and nonshared environmental correlations between NDDs, stratified by age categories. 52
Supplementary Table 21. Genetic, shared and nonshared environmental correlations between NDDs and DICCs, stratified by age categories. 53
Supplementary Table 22. Heritability, shared and nonshared environmental influences on NDDs, stratified by countries. 54

Supplementary Table 23. Genetic, shared and nonshared environmental correlations between NDDs, stratified by countries. 56
Supplementary Table 24. Genetic, shared and nonshared environmental correlations between NDDs and DICCs, stratified by countries. 57
Supplementary Table 25. Heritability, shared and nonshared environmental influences on NDDs, stratified by the percentage of individuals of European ancestry. 58
Supplementary Table 26. Genetic, shared and nonshared environmental correlations between NDDs, stratified by the percentage of individuals of European ancestry. 59
Supplementary Table 27. Genetic, shared and nonshared environmental correlations between NDDs and DICCs, stratified by the percentage of individuals of European ancestry. 60
Supplementary Table 28. Heritability, shared and nonshared environmental influences on NDDs, stratified by measurement scales. 61
Supplementary Table 29. Genetic, shared and nonshared environmental correlations between NDDs, stratified by measurement scales. 62
Supplementary Table 30. Genetic, shared and nonshared environmental correlations between NDDs and DICCs, stratified by measurement scales. 63
Supplementary Table 31. Overview of family-based studies using samples of males and females combined. Co-occurrences between disorders annotated with an asterisk (*) indicate pairs of disorders for which meta-analysis could not be performed. 64
Supplementary Table 32. Overview of family-based studies using male samples. Co-occurrences between disorders annotated with an asterisk (*) indicate pairs of disorders for which meta-analysis could not be performed. 84
Supplementary Table 33. Overview of family-based studies using female samples. Co-occurrences between disorders annotated with an asterisk (*) indicate pairs of disorders for which meta-analysis could not be performed. 89
Supplementary Table 34. Overview of SNP-based studies using samples of males and females combined. Disorders annotated with an asterisk (*) indicate disorders for which meta-analysis could not be performed. 94
Supplementary Table 35. Overview of SNP-based studies using male samples. Disorders annotated with an asterisk (*) indicate disorders for which meta-analysis could not be performed. 97
Supplementary Table 36. Overview of SNP-based studies using female samples. Disorders annotated with an asterisk (*) indicate disorders for which meta-analysis could not be performed. 98
Supplementary Table 37. Heritability, shared and nonshared environmental influences on NDDs, stratified by designs. 99
Supplementary Table 38. Genetic, shared and nonshared environmental correlations between NDDs, stratified by designs. 101
Supplementary Table 39. Genetic, shared and nonshared environmental correlations between NDDs and DICCs, stratified by designs. 102
Supplementary Table 40. Heritability, shared and nonshared environmental influences on NDDs, stratified by models 103
Supplementary Table 41. Genetic, shared and nonshared environmental correlations between NDDs, stratified by models. 105
Supplementary Table 42. Genetic, shared and nonshared environmental correlations between NDDs and DICCs, stratified by models. 106
Supplementary Table 43. Heritability, shared and nonshared environmental influences on NDDs, stratified by raters. 107

Supplementary Table 44. Genetic, shared and nonshared environmental correlations between NDDs, stratified by raters. 109
Supplementary Table 45. Genetic, shared and nonshared environmental correlations between NDDs and DICCs, stratified by raters. 110
Supplementary Table 46. Heritability, shared and nonshared environmental influences on NDDs, stratified by number of covariates included in analyses. 111
Supplementary Table 47. Genetic, shared and nonshared environmental correlations between NDDs, stratified by number of covariates included in analyses............................. 113
Supplementary Table 48. Genetic, shared and nonshared environmental correlations between NDDs and DICCs, stratified by number of covariates included in analyses.......... 114
Supplementary Table 49. Heritability, shared and nonshared environmental influences on NDDs, stratified by measurement instruments. 115
Supplementary Table 50. Genetic, shared and nonshared environmental correlations between NDDs, stratified by measurement instruments. 118

Supplementary Table 1. Heritability, shared and nonshared environmental influences on NDDs.

NDDs	Family h^2 (SE)	N	Family c^2 (SE)	N	Family e^2 (SE)	N	SNP h^2 (SE)	N
**NDDs combined**	0.66 (0.03)	236	0.17 (0.02)	127	0.29 (0.02)	195	0.19 (0.03)	29
**Intellectual disabilities**	0.86 (0.44)	2	-	-	0.1 (0.16)	2	-	-
**Communication disorders**	0.64 (0.19)	23	0.35 (0.06)	15	0.21 (0.04)	18	0.32 (0.14)	4
**ASD**	0.76 (0.11)	36	0.13 (0.05)	14	0.27 (0.03)	28	0.14 (0.04)	15
**ADHD**	0.67 (0.04)	121	0.11 (0.02)	48	0.3 (0.02)	107	0.20 (0.04)	14
**Specific learning disorders**	0.62 (0.04)	89	0.19 (0.02)	65	0.24 (0.02)	67	0.30 (0.08)	9
**Motor disorders**	0.74 (0.08)	6	0.13 (0.11)	3	0.38 (0.11)	6	-	-

Note. H^2= heritability; c^2= shared environmental influences; e^2= nonshared environmental influences; N= number of studies identified; SE= standard error.

Supplementary Table 2. Heritability, shared and nonshared environmental influences on NDDs, stratified by specific phenotypic sub-categories.

**Specific phenotypes from family-based studies**							**Specific phenotypes from SNP-based studies**		
**NDDs**	**Family h^2 (SE)**	**N**	**Family c^2 (SE)**	**N**	**Family e^2 (SE)**	**N**	**NDDs**	**SNP h^2 (SE)**	**N**
**Intellectual disabilities**									
Learning disability	0.86 (0.44)	2	-	-	0.1 (0.16)	2	-	-	-
**Communication disorders**									
Language difficulty	0.65 (0.2)	20	0.36 (0.07)	13	0.21 (0.04)	15	Language difficulty	0.32 (0.14)	4
Specific language impairment	0.87 (0.6)	2	-	-	-	-	-	-	-
Speech	0.8 (0.17)	2	-	-	0.2 (0.15)	2	-	-	-
Stuttering	0.58 (0.17)	2	-	-	0.21 (0.12)	2	-	-	-
Syntax	0.65 (0.37)	2	-	-	0.49 (0.24)	2	-	-	-
**ASD**									
ASD	0.79 (0.14)	26	0.06 (0.04)	12	0.26 (0.03)	19	ASD	0.13 (0.04)	10
CIs	0.76 (0.09)	8	-	-	0.27 (0.06)	5	Sis	0.2 (0.09)	6
RRBIs	0.83 (0.49)	10	0.24 (0.24)	2	0.35 (0.09)	6	-	-	-
Sis	0.67 (0.05)	15	0.31 (0.22)	3	0.3 (0.05)	11	-	-	-
Strict autism	0.51 (0.28)	2	-	-	-	-	-	-	-
**ADHD**									
ADHD	0.7 (0.05)	54	0.12 (0.03)	22	0.3 (0.03)	47	ADHD	0.21 (0.04)	11
Hyperactivity	0.66 (0.16)	2	-	-	0.38 (0.11)	2	Hyperactivity/Impulsivity	0.13 (0.11)	5
Impulsivity	0.76 (0.07)	2	-	-	0.24 (0.08)	2	Inattention	0.27 (0.17)	4
Hyperactivity/Impulsivity	0.69 (0.06)	63	0.16 (0.06)	24	0.27 (0.03)	56	-	-	-
Inattention	0.65 (0.05)	65	0.08 (0.03)	26	0.28 (0.02)	58	-	-	-
**Specific learning disorders**									
Dyslexia	0.62 (0.04)	76	0.19 (0.02)	55	0.23 (0.02)	55	-	-	-
Dysgraphia	0.56 (0.18)	3	0.08 (0.08)	3	0.38 (0.12)	3	-	-	-

Dyscalculia	0.55 (0.04)	30	0.19 (0.04)	24	0.27 (0.02)	25	-	-	-
Decoding	0.69 (0.14)	7	0.17 (0.1)	6	0.15 (0.06)	6	-	-	-
Grammar	0.55 (0.1)	2	0.3 (0.24)	2	0.26 (0.1)	2	-	-	-
Nonword reading	0.67 (0.13)	3	-	-	-	-	-	-	-
Orthographic skills	0.49 (0.15)	4	0.46 (0.18)	2	-	-	-	-	-
Phonological skills	0.59 (0.09)	13	0.2 (0.08)	11	0.23 (0.06)	10	-	-	-
Rapid naming	0.6 (0.12)	7	0.17 (0.13)	5	0.25 (0.08)	5	-	-	-
Reading difficulty	0.62 (0.04)	51	0.19 (0.03)	33	0.23 (0.03)	34	-	-	-
Reading comprehension	0.56 (0.07)	11	0.19 (0.07)	10	0.26 (0.05)	10	-	-	-
Reading fluency	0.64 (0.13)	5	0.16 (0.09)	4	0.25 (0.06)	4	-	-	-
Spelling	0.62 (0.11)	8	0.14 (0.08)	6	0.23 (0.06)	6	-	-	-
Vocabulary	0.25 (0.14)	4	0.57 (0.15)	4	0.18 (0.07)	4	-	-	-
Word reading	0.65 (0.08)	16	0.22 (0.06)	13	0.12 (0.04)	13	-	-	-
Writing difficulty	0.56 (0.18)	3	0.08 (0.08)	3	0.38 (0.12)	3	-	-	-
Calculations	0.39 (0.13)	3	-	-	0.55 (0.23)	2	-	-	-
Mathematic difficulty	0.57 (0.04)	27	0.19 (0.04)	22	0.25 (0.02)	22	-	-	-
Mathematic fluency	0.52 (0.14)	5	0.21 (0.14)	4	0.27 (0.09)	4	-	-	-
Mathematic problems solving	0.36 (0.19)	2	0.28 (0.19)	2	0.36 (0.13)	2	-	-	-
**Motor disorders**									
Coordination	0.82 (0.07)	2	-	-	0.38 (0.26)	2	-	-	-
DCD	0.69 (0.13)	2	0.12 (0.15)	2	0.43 (0.2)	3	-	-	-
Motor control	0.68 (0.12)	2	-	-	0.41 (0.33)	2	-	-	-
Tics	0.56 (0.17)	2	-	-	0.44 (0.16)	2	-	-	-

Note. H^2= heritability; c^2= shared environmental influences; e^2= nonshared environmental influences; N= number of studies identified; SE= standard error; Sis= social impairments; CIs= communication impairments; RRBIs= restrictive, repetitive behaviours and interests; DCD= developmental coordination disorder.

Supplementary Table 3. Genetic, shared and nonshared environmental correlations between NDDs.

NDDs	Family rA (SE)	N	Family rC (SE)	N	Family rE (SE)	N	SNP rG (SE)	N
**NDDs combined**	0.47 (0.06)	37	0.63 (0.33)	16	0.17 (0.05)	22	0.39 (0.19)	6
**ASD & ADHD**	0.67 (0.3)	6	-	-	0.22 (0.13)	5	0.26 (0.14)	5
**ADHD & motor disorders**	0.9 (0.82)	2	-	-	-	-	-	-
**ADHD & specific learning disorders**	0.33 (0.04)	18	0.32 (0.14)	7	0.11 (0.04)	9	-	-
**Communication disorders & motor disorders**	0.33 (0.16)	2	-	-	-	-	-	-
**Communication disorders & specific learning disorders**	0.66 (0.15)	2	-	-	-	-	-	-

Note. rA/rG= genetic correlation; rC= shared environmental correlation; rE= nonshared environmental correlation; N= number of studies identified; SE= standard error.

Supplementary Table 4. Genetic, shared and nonshared environmental correlations between NDDs, stratified by specific phenotypic sub-categories.

**NDDs**	**Family rA (SE)**	**N**	**Family rC (SE)**	**N**	**Family rE (SE)**	**N**
**ASD & ADHD**						
ASD & ADHD	0.71 (0.27)	4	-	-	0.27 (0.11)	3
Hyperactivity & Sis	0.22 (0.19)	2	-	-	0.02 (0.08)	2
Inattention & RRBIs	0.16 (0.11)	2	-	-	0.09 (0.11)	2
Inattention & Sis	0.27 (0.24)	2	-	-	0.03 (0.08)	2
**ADHD & motor disorders**						
ADHD & DCD	0.91 (0.8)	2	-	-	-	-
**ADHD & specific learning disorders**						
ADHD & Dyslexia	0.33 (0.04)	17	0.32 (0.15)	7	0.11 (0.04)	9
ADHD & Dyscalculia	0.29 (0.11)	2	-	-	0.09 (0.1)	2
ADHD & Reading difficulty	0.32 (0.07)	6	0.12 (0.11)	3	0.1 (0.08)	3
Hyperactivity & Reading difficulty	0.20 (0.06)	11	0.66 (0.19)	4	0.03 (0.05)	6
Inattention & Reading difficulty	0.39 (0.08)	13	0.43 (0.26)	5	0.16 (0.06)	7
Inattention & Maths difficulty	0.32 (0.11)	2	-	-	0.15 (0.1)	2
**Communication disorders & specific learning disorders**						
Specific language disorder & dyslexia	0.66 (0.15)	2	-	-	-	-
Note. rA/rG= genetic correlation; rC= shared environmental correlation; rE= nonshared environmental correlation; N= number of studies identified; SE= standard error; Sis= social impairments; RRBIs= restrictive, repetitive behaviours and interests; DCD= developmental coordination disorder.						

Supplementary Table 5. Genetic, shared and nonshared environmental correlations between NDDs and DICCs.

**NDDs and DICCs**	**Family rA (SE)**	**N**	**Family rC (SE)**	**N**	**Family rE (SE)**	**N**
**NDDs and DICCs combined**	0.62 (0.20)	15	0.88 (0.34)	11	0.38 (0.14)	13
**ADHD & conduct disorder**	0.66 (0.36)	6	0.94 (0.71)	3	0.11 (0.08)	5
**ADHD & oppositional defiant disorder**	0.66 (0.18)	6	0.96 (0.57)	4	0.54 (0.25)	5
**ASD & conduct disorder**	0.35 (0.10)	3	0.88 (0.57)	3	0.07 (0.08)	3
Note. rA/rG= genetic correlation; rC= shared environmental correlation; rE= nonshared environmental correlation; N= number of studies identified; SE= standard error.						

Supplementary Table 6. Genetic, shared and nonshared environmental correlations between NDDs and DICCs, stratified by specific phenotypic sub-categories.

**NDDs and DICCs**	**Family rA (SE)**	**N**	**Family rC (SE)**	**N**	**Family rE (SE)**	**N**
**ADHD & oppositional defiant disorder**						
ADHD & oppositional defiant disorder	0.58 (0.2)	5	0.95 (0.68)	3	0.29 (0.1)	4
Hyperactivity & oppositional defiant disorder	0.8 (0.57)	2	0.87 (0.86)	2	0.87 (0.74)	2
Inattention & oppositional defiant disorder	0.52 (0.1)	2	-	-	0.49 (0.11)	2
Note. rA/rG= genetic correlation; rC= shared environmental correlation; rE= nonshared environmental correlation; N= number of studies identified; SE= standard error.						

Supplementary Table 7. Proportion of variance in heritability, shared and nonshared environmental influences on NDDs accounted for by heterogeneity.

**NDDs**	**Family h^2**			**Family c^2**			**Family e^2**			**SNP h^2**		
	I^2_t	I^2_b	I^2_w	I^2_t	I^2_b	I^2_w	I^2_t	I^2_b	I^2_w	I^2_t	I^2_b	I^2_w
**NDDs combined**	0.75	0.53	0.21	0.18	<0.001	0.18	0.38	0.21	0.17	<0.001	<0.001	<0.001
**Intellectual disabilities**	0.84	0.42	0.42	-	-	-	<0.001	<0.001	<0.001	-	-	-
**Communication disorders**	0.82	0.74	0.09	0.21	<0.001	0.21	0.09	<0.001	0.9	<0.001	<0.001	<0.001
**ASD**	0.86	0.78	0.07	0.41	<0.001	0.41	0.11	<0.001	0.11	<0.001	<0.001	<0.001
**ADHD**	0.78	0.54	0.24	0.03	0.03	<0.001	0.43	<0.001	0.43	<0.001	<0.001	<0.001
**Specific learning disorders**	0.47	0.33	0.14	<0.001	<0.001	<0.001	0.05	0.05	<0.001	<0.001	<0.001	<0.001
**Motor disorders**	0.36	0.18	0.18	<0.001	<0.001	<0.001	0.37	0.18	0.18	-	-	-

Note. H^2= heritability; c^2= shared environmental influences; e^2= nonshared environmental influences; I^2_t= total variance accounted for by heterogeneity; I^2_b= between-cluster heterogeneity; I^2_w= within-cluster heterogeneity.

Supplementary Table 8. Proportion of variance in genetic, shared and nonshared environmental correlations between NDDs accounted for by heterogeneity.

NDDs	Family rA			Family rC			Family rE			SNP rG		
	I^2_t	I^2_b	I^2_w	I^2_t	I^2_b	I^2_w	I^2_t	I^2_b	I^2_w	I^2_t	I^2_b	I^2_w
**NDDs combined**	0.75	<0.001	0.75	0.95	0.36	0.59	0.24	0.24	<0.001	0.49	0.33	0.16
**ASD & ADHD**	0.94	0.65	0.29	-	-	-	0.62	0.62	<0.001	0.24	<0.001	0.24
**ADHD & motor disorders**	0.99	0.49	0.49	-	-	-	-	-	-	-	-	-
**ADHD & specific learning disorders**	<0.001	<0.001	<0.001	0.53	0.06	0.47	<0.001	<0.001	<0.001	-	-	-
**Communication disorders & motor disorders**	<0.001	<0.001	<0.001	-	-	-	-	-	-	-	-	-
**Communication disorders & specific learning disorders**	<0.001	<0.001	<0.001	-	-	-	-	-	-	-	-	-

Note. rA/rG= genetic correlation; rC= shared environmental correlation; rE= nonshared environmental correlation; I^2_t= total variance accounted for by heterogeneity; I^2_b= between-cluster heterogeneity; I^2_w= within-cluster heterogeneity.

Supplementary Table 9. Proportion of variance in genetic, shared and nonshared environmental correlations between NDDs and DICCs accounted for by heterogeneity.

**NDDs and DICCs**	**Family rA**			**Family rC**			**Family rE**		
	**I^2_t**	**I^2_b**	**I^2_w**	**I^2_t**	**I^2_b**	**I^2_w**	**I^2_t**	**I^2_b**	**I^2_w**
**NDDs and DICCs combined**	0.93	0.55	0.38	95	0	95	91	0	91
**ADHD & conduct disorder**	0.93	0.46	0.46	96	48	48	<0.001	<0.001	<0.001
**ADHD & oppositional defiant disorder**	0.83	0.42	0.42	94	47	47	93	46	46
**ASD & conduct disorder**	<0.001	<0.001	<0.001	67	<0.001	67	<0.001	<0.001	<0.001

Note. rA/rG= genetic correlation; rC= shared environmental correlation; rE= nonshared environmental correlation; I^2_t= total variance accounted for by heterogeneity; I^2_b= between-cluster heterogeneity; I^2_w= within-cluster heterogeneity.

Supplementary Table 10. Proportion of variance in heritability, shared and nonshared environmental influences on NDDs accounted for by heterogeneity, following exclusion of studies identified as outliers.

**NDDs**	**Family h^2**		**Family c^2**		**Family e^2**		**SNP h^2**	
	**I^2_t**	**N_r**	**I^2_t**	**N_r**	**I^2_t**	**N_r**	**I^2_t**	**N_r**
**NDDs combined**	0.64	85	0.53	71	0.64	69	0.69	25
**Intellectual disabilities**	-	-	-	-	-	-	-	-
**Communication disorders**	0.84	16	0.76	14	0.82	11	-	-
**ASD**	0.95	19	0.43	9	0.89	18	0.77	12
**ADHD**	0.86	45	0.56	29	0.69	47	0.75	12
**Specific learning disorders**	0.52	49	0.63	44	0.69	27	-	-
**Motor disorders**	0.91	6	-	-	0.92	5	-	-

Note. H^2= heritability; c^2= shared environmental influences; e^2= nonshared environmental influences; N_r= number of studies remaining after exclusion of outliers; I^2_t= total variance accounted for by heterogeneity; -= no outliers detected.

Supplementary Table 11. Proportion of variance in genetic, shared and nonshared environmental correlations between NDDs accounted for by heterogeneity, following exclusion of studies identified as outliers.

**NDDs**	**Family rA**		**Family rC**		**Family rE**		**SNP rG**	
	I^2_t	N_r	I^2_t	N_r	I^2_t	N_r	I^2_t	N_r
**NDDs combined**	0.75	17	0.98	6	0.94	14	-	-
**ASD & ADHD**	0.99	5	0.99	4	0.94	5	-	-
**ADHD & motor disorders**	-	-	-	-	-	-	-	-
**ADHD & specific learning disorders**	0.44	9	0.91	6	0.75	7	-	-
**Communication disorders & motor disorders**	-	-	-	-	-	-	-	-
**Communication disorders & specific learning disorders**	-	-	-	-	-	-	-	-

Note. rA/rG= genetic correlation; rC= shared environmental correlation; rE= nonshared environmental correlation; N_r= number of studies remaining after exclusion of outliers; I^2_t= total variance accounted for by heterogeneity; -= no outliers detected.

Supplementary Table 12. Proportion of variance in genetic, shared and nonshared environmental correlations between NDDs and DICCs accounted for by heterogeneity, following exclusion of studies identified as outliers.

NDDs and DICCs	Family rA		Family rC		Family rE	
	I^2_t	N_r	I^2_t	N_r	I^2_t	N_r
**NDDs and DICCs combined**	0.96	10	0.90	6	0.92	9
**ADHD & conduct disorder**	0.73	6	-	-	0.74	5
**ADHD & oppositional defiant disorder**	-	-	-	-	0.88	4
**ASD & conduct disorder**	-	-	-	-	-	-

Note. rA/rG= genetic correlation; rC= shared environmental correlation; rE= nonshared environmental correlation; N_r= number of studies remaining after exclusion of outliers; I^2_t= total variance accounted for by heterogeneity; -= no outliers detected.

Supplementary Table 13. Results of Egger's regression for studies addressing heritability and environmental influences on NDDs.

NDDs	Family h^2			Family c^2			Family e^2			SNP h^2		
	**Z**	**P**	**Estimate (95% CIs)**	**Z**	**P**	**Estimate (95% CIs)**	**Z**	**P**	**Estimate (95% CIs)**	**Z**	**P**	**Estimate (95% CIs)**
**NDDs combined**	0.0	<0.001	0.73 (0.69-0.78)	3.82	<0.001	0.03 (-0.04-0.1)	3.76	<0.001	0.17 (0.11-0.22)	1.59	0.11	0.09 (-0.05-0.22)
**Communication disorders**	0.71	0.48	0.43 (0.23-0.63)	-1.8	0.07	0.6 (0.33-0.88)	1.62	0.1	0.05 (-0.14-0.25)	1.62	0.1	0.05 (-0.14-0.25)
**ASD**	0.14	0.89	0.68 (0.57-0.79)	1.65	0.1	-0.01 (-0.15-0.14)	0.65	0.52	0.23 (0.13-0.33)	1.49	0.14	0.01 (-0.18-0.2)
**ADHD**	-2.58	0.01	0.75 (0.69-0.81)	1.83	0.07	0.01 (-0.09-0.11)	3.43	<0.001	0.17 (0.09-0.24)	-0.17	0.87	0.22 (0.01-0.42)
**Specific learning disorders**	-5.03	<0.001	0.75 (0.69-0.81)	1.52	0.13	0.08 (-0.06-0.22)	1.62	0.1	0.16 (0.06-0.27)	-0.25	0.81	0.38 (-0.34-1.11)
**Motor disorders**	-1.19	0.23	0.83 (0.71-0.95)	0.27	0.78	0.04 (-0.62-0.71)	0.81	0.42	0.09 (-0.56-0.74)	-	-	-

Note. The Egger's test uses weighted regression to determine whether there is a relationship between the effect sizes and the standard errors, which can imply asymmetry in the funnel plot, and therefore, the publication bias. Multiple comparisons correction was not applied. H^2= heritability; c^2= shared environmental influences; e^2= nonshared environmental influences; CIs= confidence intervals; Estimate= the limit estimate; -= number of parameters to be estimated was larger than the number of observations; Z= z-value of the test statistic; P= two-sided p-value.

Supplementary Table 14. Results of Egger's regression for studies addressing genetic and environmental overlap between NDDs.

NDDs	Family rA			Family rC			Family rE			SNP rG		
	Z	P	Estimate (95% CIs)	Z	P	Estimate (95% CIs)	Z	P	Estimate (95% CIs)	Z	P	Estimate (95% CIs)
**NDDs combined**	-0.24	0.81	0.45 (0.27-0.63)	1.84	0.07	0.09 (-0.36-0.54)	1.65	0.1	<0.001 (-0.2-0.2)	1.07	0.28	-0.38 (-1.61-0.85)
**ASD & ADHD**	-0.49	0.62	0.68 (-0.03-1.39)	-	-	-	0.73	0.47	0.01 (-0.5-0.52)	0.47	0.64	-0.14 (-1.71-1.44)
**ADHD & specific learning disorders**	1.08	0.28	0.22 (0.02-0.42)	1.17	0.24	-0.02 (-0.46-0.42)	1.15	0.25	-0.04 (-0.32-0.23)	-	-	-

Note. The Egger's test uses weighted regression to determine whether there is a relationship between the effect sizes and the standard errors, which can imply asymmetry in the funnel plot, and therefore, the publication bias. Multiple comparisons correction was not applied. rA/rG= genetic correlation; rC= shared environmental correlation; rE= nonshared environmental correlation; CIs= confidence intervals; Estimate= the limit estimate; -= number of parameters to be estimated was larger than the number of observations; Z= z-value of the test statistic; P= two-sided p-value.

Supplementary Table 15. Results of Egger's regression for studies addressing genetic and environmental overlap between NDDs and DICCs.

**NDDs and DICCs**	**Family rA**			**Family rC**			**Family rE**		
	**Z**	**P**	**Estimate (95% CIs)**	**Z**	**P**	**Estimate (95% CIs)**	**Z**	**P**	**Estimate (95% CIs)**
**NDDs and DICCs combined**	-0.79	0.43	0.63 (0.26, 1)	3.62	<0.001	-0.17 (-0.42, 0.07)	0.78	0.44	0.12 (-0.11, 0.35)
**ADHD & conduct disorder**	0.32	0.75	0.38 (-0.28, 1.04)	2.88	<0.001	-0.43 (-0.95, 0.09)	1.1	0.27	-0.15 (-0.64, 0.34)
**ADHD & oppositional defiant disorder**	-0.66	0.51	0.73 (0.32, 1.14)	1.46	0.14	0.06 (-0.78, 0.89)	-0.79	0.43	0.63 (0.14, 1.12)
**ASD & conduct disorder**	0.52	0.60	-0.06 (-1.61, 1.49)	0.45	0.65	-0.24 (-4.32, 3.84)	0.85	0.40	-0.16 (-0.71, 0.38)

Note. The Egger's test uses weighted regression to determine whether there is a relationship between the effect sizes and the standard errors, which can imply asymmetry in the funnel plot, and therefore, the publication bias. Multiple comparisons correction was not applied. rA/rG= genetic correlation; rC= shared environmental correlation; rE= nonshared environmental correlation; CIs= confidence intervals; Estimate= the limit estimate; Z= z-value of the test statistic; P= two-sided p-value.

Supplementary Table 16. Sex-specific heritability, shared and nonshared environmental influences on NDDs.

NDDs	Males		Females		Males		Females		Males		Females		Males		Females	
	Family h^2 (SE)	N	Family h^2 (SE)	N	Family c^2 (SE)	N	Family c^2 (SE)	N	Family e^2 (SE)	N	Family e^2 (SE)	N	SNP h^2 (SE)	N	SNP h^2 (SE)	N
**NDDs combined**	0.65 (0.06)	68	0.67 (0.06)	67	0.35 (0.08)	36	0.28 (0.08)	34	0.31 (0.04)	63	0.33 (0.04)	61	0.19 (0.07)	2	0.09 (0.10)	2
**Intellectual disabilities**	-	-	-	-	-	-	-	-	-	-	-	-	-	-	-	-
**Communication disorders**	0.64 (0.33)	4	0.67 (0.42)	4	0.35 (0.14)	3	0.35 (0.16)	3	0.28 (0.14)	4	0.29 (0.14)	4	-	-	-	-
**ASD**	0.64 (0.16)	21	0.68 (0.09)	23	0.46 (0.20)	12	0.30 (0.14)	12	0.28 (0.06)	19	0.24 (0.02)	21	-	-	-	-
**ADHD**	0.68 (0.08)	38	0.71 (0.08)	38	0.38 (0.17)	14	0.13 (0.07)	12	0.32 (0.06)	36	0.34 (0.06)	35	0.20 (0.08)	2	0.13 (0.11)	2
**Specific learning disorders**	0.61 (0.08)	9	0.61 (0.09)	9	0.21 (0.07)	8	0.18 (0.06)	8	0.30 (0.07)	8	0.34 (0.08)	8	-	-	-	-
**Motor disorders**	0.59 (0.36)	2	0.58 (0.34)	2	-.	-	-.	-	0.24 (0.09)	2	0.27 (0.08)	2	-	-	-	-

Note. H^2= heritability; c^2= shared environmental influences; e^2= nonshared environmental influences; N= number of studies identified; SE= standard error.

Supplementary Table 17. Sex-specific genetic, shared and nonshared environmental correlations between NDDs.

NDDs	Males		Females		Males		Females		Males		Females	
	**Family rA (SE)**	**N**	**Family rA (SE)**	**N**	**Family rC (SE)**	**N**	**Family rC (SE)**	**N**	**Family rE (SE)**	**N**	**Family rE (SE)**	**N**
**NDDs combined**	0.88 (0.54)	4	0.37 (0.24)	2	-	-	-	-	0.09 (0.08)	3	0.10 (0.11)	2
**ASD & ADHD**	0.79 (0.42)	2	-	-	-	-	-	-	0.20 (0.14)	2	-	-

Note. rA/rG= genetic correlation; rC= shared environmental correlation; rE= nonshared environmental correlation; N= number of studies identified; SE= standard error.

Supplementary Table 18. Sex-specific genetic, shared and nonshared environmental correlations between NDDs and DICCs.

**NDDs and DICCs**	**Males**		**Females**		**Males**		**Females**		**Males**		**Females**	
	**Family rA (SE)**	**N**	**Family rA (SE)**	**N**	**Family rC (SE)**	**N**	**Family rC (SE)**	**N**	**Family rE (SE)**	**N**	**Family rE (SE)**	**N**
**NDDs and DICCs combined**	-	-	0.75 (0.58)	2	-	-	-	-	-	-	0.06 (0.12)	2
**ADHD & conduct disorder**	-	-	0.75 (0.58)	2	-	-	-	-	-	-	0.06 (0.12)	2
Note. rA/rG= genetic correlation; rC= shared environmental correlation; rE= nonshared environmental correlation; N= number of studies identified; SE= standard error.												

Supplementary Table 19. Heritability, shared and nonshared environmental influences on NDDs, stratified by age categories.

NDDs	Family h^2 (SE)	N	Family c^2 (SE)	N	Family e^2 (SE)	N	SNP h^2 (SE)	N
**NDDs combined**								
Childhood (4-7y)	0.63 (0.03)	54	0.21 (0.04)	36	0.27 (0.03)	51	0.24 (0.11)	6
Middle childhood (8-10y)	0.68 (0.04)	54	0.12 (0.03)	33	0.25 (0.02)	51	0.26 (0.08)	7
Adolescence (11-24y)	0.62 (0.04)	79	0.17 (0.03)	47	0.35 (0.03)	72	0.23 (0.07)	13
Childhood & middle childhood (4-10y)	0.67 (0.06)	14	0.33 (0.08)	7	0.21 (0.05)	11	-	-
Childhood & adolescence (4-24y)	0.72 (0.07)	40	0.20 (0.05)	19	0.20 (0.03)	31	0.17 (0.03)	11
Middle childhood & adolescence (8-24y)	0.69 (0.04)	50	0.14 (0.04)	19	0.28 (0.03)	31	-	-
**Communication disorders**								
Childhood (4-7y)	0.56 (0.08)	15	0.41 (0.07)	12	0.21 (0.05)	14	-	-
Adolescence (11-24y)	0.45 (0.07)	7	0.26 (0.08)	5	0.27 (0.06)	5	0.32 (0.16)	3
Childhood & middle childhood (4-10y)	0.92 (0.75)	2	-	-	-	-	-	-
**ASD**								
Childhood (4-7y)	0.69 (0.16)	3	-	-	0.31 (0.08)	3	-	-
Middle childhood (8-10y)	0.88 (0.40)	11	0.13 (0.07)	5	0.22 (0.05)	9	0.26 (0.12)	4
Adolescence (11-24y)	0.61 (0.07)	9	0.31 (0.17)	4	0.28 (0.07)	7	0.16 (0.09)	7
Childhood & adolescence (4-24y)	0.79 (0.17)	5	0.02 (0.05)	3	0.21 (0.13)	4	0.13 (0.05)	7
Middle childhood & adolescence (8-24y)	0.75 (0.07)	10	0.13 (0.08)	3	0.29 (0.04)	8	-	-
**ADHD**								
Childhood (4-7y)	0.64 (0.05)	21	0.07 (0.06)	7	0.33 (0.04)	19	0.10 (0.17)	2
Middle childhood (8-10y)	0.65 (0.07)	28	0.07 (0.04)	12	0.30 (0.04)	28	0.19 (0.12)	3
Adolescence (11-24y)	0.64 (0.05)	44	0.23 (0.08)	17	0.37 (0.03)	39	0.09 (0.13)	3
Childhood & middle childhood (4-10y)	0.68 (0.10)	7	0.39 (0.13)	2	0.27 (0.07)	6	-	-
Childhood & adolescence (4-24y)	0.73 (0.08)	24	0.19 (0.06)	10	0.20 (0.04)	20	0.21 (0.05)	7

Middle childhood & adolescence (8-24y)	0.73 (0.06)	19	0.04 (0.07)	4	0.30 (0.04)	15	-	-
**Specific learning disorders**								
Childhood (4-7y)	0.63 (0.05)	18	0.18 (0.04)	18	0.21 (0.03)	18	0.29 (0.14)	3
Middle childhood (8-10y)	0.62 (0.06)	20	0.17 (0.04)	18	0.26 (0.03)	19	-	-
Adolescence (11-24y)	0.57 (0.03)	33	0.17 (0.03)	27	0.30 (0.03)	29	0.31 (0.09)	8
Childhood & middle childhood (4-10y)	0.59 (0.10)	6	0.24 (0.13)	5	0.24 (0.07)	6	-	-
Childhood & adolescence (4-24y)	0.61 (0.10)	11	0.22 (0.06)	8	0.20 (0.05)	8	-	-
Middle childhood & adolescence (8-24y)	0.65 (0.06)	26	0.22 (0.06)	13	0.18 (0.04)	12	-	-
**Motor disorders**								
Childhood & adolescence (4-24y)	0.73 (0.09)	4	0.21 (0.15)	2	0.20 (0.12)	3	-	-
Note. H^2= heritability; c^2= shared environmental influences; e^2= nonshared environmental influences; N= number of studies identified; SE= standard error.								

Supplementary Table 20. Genetic, shared and nonshared environmental correlations between NDDs, stratified by age categories.

NDDs	Family rA (SE)	N	Family rC (SE)	N	Family rE (SE)	N	SNP rG (SE)	N
**NDDs combined**								
Adolescence (11-24y)	0.55 (0.20)	11	0.80 (0.37)	8	0.18 (0.05)	10	0.73 (0.29)	2
Childhood & middle childhood (4-10y)	0.33 (0.09)	4	-	-	0.12 (0.10)	3	-	-
Childhood & adolescence (4-24y)	0.30 (0.06)	8	-	3	0.04 (0.07)	4	-	-
**ASD & ADHD**								
Adolescence (11-24y)	0.66 (0.49)	3	0.15 (0.07)	3	0.15 (0.07)	3	-	-
**ADHD & specific learning disorders**								
Adolescence (11-24y)	0.26 (0.07)	5	0.26 (0.11)	4	0.12 (0.06)	4	-	-
Childhood & middle childhood (4-10y)	0.43 (0.14)	3	-	-	0.12 (0.10)	3	-	-
Childhood & adolescence (4-24y)	0.25 (0.09)	3	-	-	0.05 (0.09)	2	-	-
**Communication disorders & motor disorders**								
Childhood & adolescence (4-24y)	0.33 (0.16)	2	-	-	-	-	-	-
Note. rA/rG= genetic correlation; rC= shared environmental correlation; rE= nonshared environmental correlation; N= number of studies identified; SE= standard error.								

Supplementary Table 21. Genetic, shared and nonshared environmental correlations between NDDs and DICCs, stratified by age categories.

NDDs and DICCs	Family rA (SE)	N	Family rC (SE)	N	Family rE (SE)	N
**NDDs and DICCs combined**						
Adolescence (11-24y)	0.73 (.29)	3	0.70 (0.63)	2	0.82 (0.64)	2
Childhood & adolescence (4-24y)	0.83 (0.61)	3	0.09 (0.56)	2	0.27 (0.08)	3
**ADHD & conduct disorder**						
Childhood & adolescence (4-24y)	0.90 (0.81)	2	-	-	0.15 (0.18)	2
Note. rA/rG= genetic correlation; rC= shared environmental correlation; rE= nonshared environmental correlation; N= number of studies identified; SE= standard error.						

Supplementary Table 22. Heritability, shared and nonshared environmental influences on NDDs, stratified by countries.

NDDs	Family h^2 (SE)	N	Family c^2 (SE)	N	Family e^2 (SE)	N	SNP h^2 (SE)	N
**NDDs combined**								
Australia	0.76 (0.17)	11	0.21 (0.07)	9	0.17 (0.05)	8	-	-
Australia & United States & Norway & Sweden	0.74 (0.13)	2	0.05 (0.11)	2	0.24 (0.09)	2	-	-
Canada	0.43 (0.09)	7	0.18 (0.09)	6	0.38 (0.07)	6	-	-
China	0.5 (0.15)	4	0.3 (0.13)	3	0.29 (0.12)	4	-	-
Netherlands	0.52 (0.26)	19	0.12 (0.12)	5	0.37 (0.13)	17	0.47 (0.22)	3
Norway	0.53 (0.09)	2	0.25 (0.23)	2	0.28 (0.14)	2	-	-
Sweden	0.74 (0.05)	24	0.07 (0.04)	9	0.28 (0.03)	22	-	-
United Kingdom	0.7 (0.06)	96	0.18 (0.02)	53	0.27 (0.02)	85	0.22 (0.06)	14
United States	0.61 (0.04)	77	0.22 (0.03)	44	0.32 (0.04)	53	-	-
**Intellectual disabilities**								
Sweden	0.86 (0.44)	2	-	-	0.1 (0.16)	2	-	-
**Communication disorders**								
Canada	0.32 (0.2)	2	0.38 (0.18)	2	0.35 (0.12)	2	-	-
Netherlands	0.45 (0.19)	2	-	-	0.3 (0.18)	2	-	-
United Kingdom	0.77 (0.41)	17	0.35 (0.07)	11	0.2 (0.04)	13	0.32 (0.14)	4
United States	0.71 (0.38)	2	-	-	-	-	-	-
**ASD**								
Netherlands	0.5 (0.17)	2	-	-	0.52 (0.16)	2	-	-
Sweden	0.74 (0.05)	10	0.09 (0.06)	5	0.28 (0.04)	9	-	-
United Kingdom	0.8 (0.24)	20	0.19 (0.08)	8	0.24 (0.04)	15	0.18 (0.08)	7
United States	0.8 (0.5)	3	-	-	-	-	-	-
**ADHD**								

Australia	0.83 (0.31)	7	0.26 (0.11)	6	0.11 (0.05)	5	-	-
Australia & United States & Norway & Sweden	0.73 (0.14)	2	0.03 (0.12)	2	0.26 (0.1)	2	-	-
Canada	0.45 (0.16)	3	-	-	0.38 (0.19)	2	-	-
China	0.49 (0.33)	2	0.26 (0.17)	2	0.31 (0.24)	2	-	-
Netherlands	0.52 (0.27)	15	0.05 (0.08)	4	0.28 (0.03)	12	0.42 (0.24)	2
Sweden	0.75 (0.07)	18	0.04 (0.06)	6	0.27 (0.04)	17	-	-
United Kingdom	0.71 (0.03)	42	0.2 (0.11)	14	0.29 (0.02)	39	0.08 (0.11)	4
United States	0.62 (0.06)	30	0.12 (0.06)	12	0.38 (0.05)	25	-	-
**Specific learning disorders**								
Australia	0.72 (0.11)	5	0.09 (0.07)	4	0.23 (0.06)	4	-	-
Canada	0.53 (0.13)	4	0.1 (0.11)	4	0.39 (0.09)	4	-	-
Netherlands	0.59 (0.19)	2	-	-	0.33 (0.13)	2	-	-
United Kingdom	0.59 (0.03)	33	0.17 (0.03)	26	0.29 (0.02)	29	0.31 (0.08)	8
United States	0.57 (0.05)	47	0.24 (0.04)	33	0.21 (0.03)	30	-	-
**Motor disorders**								
Sweden	0.69 (0.12)	4	0.06 (0.17)	2	0.36 (0.12)	4	-	-
Note. H^2= heritability; c^2= shared environmental influences; e^2= nonshared environmental influences; N= number of studies identified; SE= standard error.								

Supplementary Table 23. Genetic, shared and nonshared environmental correlations between NDDs, stratified by countries.

NDDs	Family rA (SE)	N	Family rC (SE)	N	Family rE (SE)	N	SNP rG (SE)	N
**NDDs combined**								
Australia	0.27 (0.08)	2	0.1 (0.09)	2	0.02 (0.08)	2	-	-
Canada	0.44 (0.24)	2	0.19 (0.2)	2	0.16 (0.15)	2	-	-
Sweden	0.8 (0.26)	3	-	-	0.36 (0.12)	2	-	-
United Kingdom	0.45 (0.07)	18	0.91 (0.29)	10	0.16 (0.04)	14	0.74 (0.28)	2
United States	0.48 (0.07)	11	0.07 (0.2)	2	-	-	-	-
**ASD & ADHD**								
Sweden	0.8 (0.25)	3	-	-	0.36 (0.12)	2	-	-
United Kingdom	0.28 (0.09)	3	-	-	0.1 (0.07)	3	-	-
**ADHD & specific learning disorders**								
Canada	0.44 (0.24)	2	0.19 (0.2)	2	0.16 (0.15)	2	-	-
United Kingdom	0.31 (0.06)	6	0.48 (0.2)	3	0.13 (0.05)	5	-	-
United States	0.44 (0.09)	8	-	-	-	-	-	-
**Communication disorders & specific learning disorders**								
United Kingdom	0.66 (0.15)	2	-	-	-	-	-	-
Note. rA/rG= genetic correlation; rC= shared environmental correlation; rE= nonshared environmental correlation; N= number of studies identified; SE= standard error.								

Supplementary Table 24. Genetic, shared and nonshared environmental correlations between NDDs and DICCs, stratified by countries.

NDDs and DICCs	Family rA (SE)	N	Family rC (SE)	N	Family rE (SE)	N
**NDDs and DICCs combined**						
Sweden	0.68 (0.41)	3	0.89 (0.55)	2	0.68 (0.64)	3
United Kingdom	0.58 (0.29)	3	0.97 (0.57)	3	0.49 (0.44)	3
United States	0.42 (0.15)	6	0.85 (0.55)	5	0.24 (0.09)	4
**ADHD & conduct disorder**						
United States	0.41 (0.17)	3	0.99 (0.28)	2	0.12 (0.14)	2
**ADHD & oppositional defiant disorder**						
United States	0.59 (0.32)	3	0.99 (0.57)	2	0.25 (0.14)	2
**ASD & conduct disorder**						
United Kingdom	0.33 (0.13)	2	0.93 (0.77)	2	0.04 (0.08)	2
Note. rA/rG= genetic correlation; rC= shared environmental correlation; rE= nonshared environmental correlation; N= number of studies identified; SE= standard error.						

Supplementary Table 25. Heritability, shared and nonshared environmental influences on NDDs, stratified by the percentage of individuals of European ancestry.

NDDs	Family h^2 (SE)	N	Family c^2 (SE)	N	Family e^2 (SE)	N	SNP h^2 (SE)	N
**NDDs combined**								
Less than 50%	0.46 (0.07)	7	0.24 (0.08)	6	0.43 (0.08)	7	-	-
50-74%	0.47 (0.08)	12	0.24 (0.08)	9	0.32 (0.13)	9	-	-
75-99%	0.71 (0.07)	37	0.24 (0.06)	15	0.25 (0.03)	32	-	-
100%	0.66 (0.06)	41	0.19 (0.04)	29	0.32 (0.05)	40	0.19 (0.03)	29
**Communication disorders**								
75-99%	0.59 (0.27)	3	0.36 (0.15)	3	0.16 (0.11)	3	-	-
100%	0.56 (0.09)	11	0.33 (0.1)	8	0.24 (0.06)	10	0.32 (0.14)	4
**ASD**								
75-99%	0.91 (0.57)	9	-	-	0.29 (0.06)	6	-	-
**ADHD**								
Less than 50%	0.41 (0.12)	3	0.17 (0.15)	2	0.54 (0.09)	3	-	-
50-74%	0.49 (0.11)	5	0.18 (0.13)	3	0.35 (0.19)	4	-	-
75-99%	0.73 (0.06)	20	0.17 (0.07)	6	0.27 (0.04)	19	-	-
100%	0.67 (0.04)	11	0.04 (0.09)	3	0.39 (0.05)	10	0.2 (0.04)	14
**Specific learning disorders**								
Less than 50%	0.54 (0.16)	5	0.25 (0.09)	5	0.28 (0.06)	5	-	-
50-74%	0.52 (0.1)	7	0.24 (0.1)	6	0.24 (0.06)	6	-	-
75-99%	0.55 (0.09)	7	0.29 (0.12)	6	0.19 (0.06)	6	-	-
100%	0.61 (0.04)	22	0.16 (0.04)	19	0.3 (0.07)	21	0.3 (0.08)	9
**Motor disorders**								
100%	0.8 (0.05)	2	-	-	0.47 (0.27)	2	-	-
Note. H^2= heritability; c^2= shared environmental influences; e^2= nonshared environmental influences; N= number of studies identified; SE= standard error.								

Supplementary Table 26. Genetic, shared and nonshared environmental correlations between NDDs, stratified by the percentage of individuals of European ancestry.

NDDs	Family rA (SE)	N	Family rC (SE)	N	Family rE (SE)	N	SNP rG (SE)	N
**NDDs combined**								
75-99%	0.63 (0.44)	2	-	-	-	-	-	-
100%	0.54 (0.1)	4	0.93 (0.18)	2	0.24 (0.09)	4	0.39 (0.19)	6
**ASD & ADHD**								
100%	-	-	-	-	-	-	0.26 (0.14)	5
**ADHD & specific learning disorders**								
100%	0.48 (0.13)	2	-	-	0.26 (0.15)	2	-	-
Note. rA/rG= genetic correlation; rC= shared environmental correlation; rE= nonshared environmental correlation; N= number of studies identified; SE= standard error.								

Supplementary Table 27. Genetic, shared and nonshared environmental correlations between NDDs and DICCs, stratified by the percentage of individuals of European ancestry.

NDDs and DICCs	Family rA (SE)	N	Family rC (SE)	N	Family rE (SE)	N
**NDDs and DICCs combined**						
75-99%	0.57 (0.25)	3	0.88 (0.87)	2	-	-
100%	0.71 (0.31)	2	0.89 (0.85)	2	0.74 (0.49)	2
**ADHD & conduct disorder**						
75-99%	0.41 (0.22)	2	-	-	-	-
**ADHD & oppositional defiant disorder**						
75-99%	0.61 (0.48)	2	-	-	-	-
Note. rA/rG= genetic correlation; rC= shared environmental correlation; rE= nonshared environmental correlation; N= number of studies identified; SE= standard error.						

Supplementary Table 28. Heritability, shared and nonshared environmental influences on NDDs, stratified by measurement scales.

NDDs	Family h^2 (SE)	N	Family c^2 (SE)	N	Family e^2 (SE)	N	SNP h^2 (SE)	N
**NDDs combined**								
Categorical	0.77 (0.07)	28	0.19 (0.08)	12	0.28 (0.06)	25	0.17 (0.03)	12
Continuous	0.64 (0.03)	215	0.16 (0.02)	116	0.28 (0.01)	175	0.25 (0.06)	17
**Intellectual disabilities**								
Categorical	0.86 (0.44)	2	-	-	0.1 (0.16)	2	-	-
**Communication disorders**								
Categorical	0.67 (0.24)	6	0.47 (0.12)	4	0.13 (0.06)	5	-	-
Continuous	0.65 (0.2)	19	0.3 (0.06)	12	0.25 (0.05)	14	0.32 (0.14)	4
**ASD**								
Categorical	0.83 (0.08)	11	0.03 (0.08)	5	0.18 (0.06)	9	0.13 (0.05)	7
Continuous	0.72 (0.15)	29	0.18 (0.07)	9	0.27 (0.03)	23	0.2 (0.08)	8
**ADHD**								
Categorical	0.79 (0.1)	13	0.05 (0.08)	5	0.26 (0.07)	12	0.21 (0.04)	8
Continuous	0.66 (0.04)	109	0.11 (0.03)	43	0.31 (0.02)	96	0.16 (0.1)	6
**Specific learning disorders**								
Continuous	0.62 (0.04)	89	0.19 (0.02)	65	0.24 (0.02)	67	0.31 (0.08)	8
**Motor disorders**								
Categorical	0.72 (0.08)	5	0.13 (0.11)	3	0.38 (0.12)	6	-	-
Continuous	0.69 (0.2)	3	-	-	-	-	-	-
Note. H^2= heritability; c^2= shared environmental influences; e^2= nonshared environmental influences; N= number of studies identified; SE= standard error.								

Supplementary Table 29. Genetic, shared and nonshared environmental correlations between NDDs, stratified by measurement scales.

NDDs co-occurrences	Family rA (SE)	N	Family rC (SE)	N	Family rE (SE)	N	SNP rG (SE)	N
**NDDs combined**								
Categorical	0.56 (0.32)	3	-	-	-	-	-	-
Continuous	0.47 (0.06)	34	0.67 (0.33)	15	0.18 (0.05)	21	0.74 (0.28)	2
**ASD & ADHD**								
Continuous	0.56 (0.34)	5	-	-	0.22 (0.13)	5	-	-
**ADHD & motor disorders**								
Categorical	0.9 (0.82)	2	-	-	-	-	-	-
**ADHD & specific learning disorders**								
Continuous	0.33 (0.04)	17	0.32 (0.14)	7	0.11 (0.04)	9	-	-
**Communication disorders & specific learning disorders**								
Continuous	0.66 (0.15)	2	-	-	-	-	-	-
Note. rA/rG= genetic correlation; rC= shared environmental correlation; rE= nonshared environmental correlation; N= number of studies identified; SE= standard error.								

Supplementary Table 30. Genetic, shared and nonshared environmental correlations between NDDs and DICCs, stratified by measurement scales.

Co-occurrences between NDDs and DICCs	Family rA (SE)	N	Family rC (SE)	N	Family rE (SE)	N
**NDDs and DICCs combined**						
Continuous	0.62 (0.19)	15	0.88 (0.34)	11	0.38 (0.14)	13
**ADHD & conduct disorder**						
Continuous	0.66 (0.36)	6	0.94 (0.71)	3	0.11 (0.08)	5
**ADHD & oppositional defiant disorder**						
Continuous	0.66 (0.18)	6	0.96 (0.57)	4	0.54 (0.25)	5
**ASD & conduct disorder**						
Continuous	0.35 (0.10)	3	0.88 (0.57)	3	0.07 (0.08)	3
Note. rA/rG= genetic correlation; rC= shared environmental correlation; rE= nonshared environmental correlation; N= number of studies identified; SE= standard error.						

Supplementary Table 31. Overview of family-based studies using samples of males and females combined. Co-occurrences between disorders annotated with an asterisk (*) indicate pairs of disorders for which meta-analysis could not be performed.

Reference	Cohort	Age category	Country
**Heritability and environmental influences on intellectual disabilities**			
Du Rietz et. al. (2021)[10]	Medical Birth Register, Multi-Generation Register	Childhood & Adolescence	Sweden
Taylor et. al. (2019)[11]	The Child and Adolescent Twin Study in Sweden (CATSS)	Middle Childhood & Adolescence	Sweden
**Heritability and environmental influences on communication disorders**			
Bishop & Hayiou-Thomas (2008)[12]	Twins Early Development Study (TEDS)	Childhood	United Kingdom
Cheesman et. al. (2017)[13]	Twins Early Development Study (TEDS)	Adolescence	United Kingdom
DeThorne et. al. (2006)[14]	Western reserve twin project (WRTP)	Childhood	United States
Hayiou-Thomas, Dale & Plomin (2012)[15]	Twins Early Development Study (TEDS)	Childhood & Middle Childhood	United Kingdom
Hayiou-Thomas, Dale & Plomin (2014)[16]	Twins Early Development Study (TEDS)	Childhood	United Kingdom
Hohnen & Stevenson (1999)[17]	Twin study in London	Childhood	United Kingdom
Tomblin & Buckwalter (1998)[18]	Twin study in Iowa	Childhood	United States
Trzaskowski, Dale & Plomin (2013)[19]	Twins Early Development Study (TEDS)	Adolescence	United Kingdom
van Beijsterveldt, Felsenfeld & Boomsma (2010)[20]	Netherlands twin register (NTR)	Childhood	Netherlands
Bishop (2002)[21]	Twin study in the United Kingdom	Childhood & Adolescence	United Kingdom
Bishop (2005)[22]	Twins Early Development Study (TEDS)	Childhood	United Kingdom
Bishop, Adams & Norbury (2006)[23]	Twins Early Development Study (TEDS)	Childhood	United Kingdom
Bishop, Laws, Adams & Norbury (2006)[24]	Twins Early Development Study (TEDS)	Childhood	United Kingdom

Bishop, North & Donlan (1996)[25]	Twin study in the United Kingdom	Childhood & Middle Childhood	United Kingdom
Dale, Rice, Rimfeld & Hayiou-Thomas (2018)[26]	Twins Early Development Study (TEDS)	Adolescence	United Kingdom
Dionne et. al. (2011)[27]	The Quebec Newborn Twin Study (QNTS)	Childhood	Canada
Dworzynski, Remington, Rijsdijk, Howell & Plomin (2007)[28]	Twins Early Development Study (TEDS)	Childhood	United Kingdom
Hoekstra, Bartels, Van Leeuwen & Boomsma (2009)[29]	Netherlands twin register (NTR)	Middle Childhood & Adolescence	Netherlands
Mimeau et. al. (2018)[30]	The Quebec Newborn Twin Study (QNTS)	Childhood	Canada
Price, Dale & Plomin (2004)[31]	Twins Early Development Study (TEDS)	Childhood	United Kingdom
Tosto et. al. (2017)[32]	Twins Early Development Study (TEDS)	Childhood	United Kingdom
Trzaskowski et. al. (2013)[33]	Twins Early Development Study (TEDS)	Adolescence	United Kingdom
Viding et. al. (2004)[34]	Twins Early Development Study (TEDS)	Childhood	United Kingdom
**Heritability and environmental influences on ASD**			
Bailey et. al. (1995)[35]	The twin study of Folstein & Rutter	Childhood & Adolescence	United Kingdom
Cheesman et. al. (2017)[13]	Twins Early Development Study (TEDS)	Adolescence	United Kingdom
Deng et. al. (2015)[36]	Twin study in China	Childhood & Adolescence	China
Du Rietz et. al. (2021)[10]	Medical Birth Register, Multi-Generation Register	Childhood & Adolescence	Sweden
Dworzynski et. al. (2008)[37]	Twins Early Development Study (TEDS)	Childhood & Middle Childhood	United Kingdom
Dworzynski, Happe, Bolton & Ronald (2009)[38]	Twins Early Development Study (TEDS)	Middle Childhood & Adolescence	United Kingdom
Frazier et. al. (2014)[39]	Interactive Autism Network (IAN)	Middle Childhood	United States
Hallet, Ronald & Happe (2009)[40]	Twins Early Development Study (TEDS)	Middle Childhood	United Kingdom
Hoekstra, Bartels, Verweij & Boomsma (2007)[41]	Netherlands twin register (NTR)	Adolescence	Netherlands
Jones et. al. (2009)[42]	Twins Early Development Study (TEDS)	Middle Childhood	United Kingdom

Lichtenstein, Carlstrom, Rastam, Gillberg & Anckarsater (2010)[1]	Swedish Twin Register	Middle Childhood	Sweden
Lundstrom et. al. (2012)[43]	The Child and Adolescent Twin Study in Sweden (CATSS)	Middle Childhood & Adolescence	Sweden
Pinto, Rijsdijk, Ronald, Asherson & Kuntsi (2016)[44]	Twins Early Development Study (TEDS)	Middle Childhood	United Kingdom
Polderman, Posthuma, De Sonnerville, Verlhulst & Boomsma (2006)[45]	Netherlands twin register (NTR)	Childhood	Netherlands
Robinson et. al. (2011)[46]	Twins Early Development Study (TEDS)	Adolescence	United Kingdom
Robinson et. al. (2012)[47]	Twins Early Development Study (TEDS)	Middle Childhood & Adolescence	United Kingdom
Ronald et. al. (2006)[48]	Twins Early Development Study (TEDS)	Middle Childhood	United Kingdom
Ronald, Happe, Price, Baron-Cohen & Plomin (2006)[49]	Twins Early Development Study (TEDS)	Middle Childhood	United Kingdom
Ronald, Larsson, Anckarsater & Lichtenstein (2014)[50]	The Child and Adolescent Twin Study in Sweden (CATSS)	Middle Childhood & Adolescence	Sweden
Ronald, Simonoff, Kuntsi, Asherson & Plomin (2008)[51]	Twins Early Development Study (TEDS)	Middle Childhood	United Kingdom
Scherff et. al. (2014)[52]	Twins Early Development Study (TEDS)	Adolescence	United Kingdom
Scourfield, Martin, Eley & McGuffin (2004)[53]	The Cardiff Study of All Wales and Northwest of England Twins (CaStANET)	Childhood & Adolescence	United Kingdom
Taylor et. al. (2018)[54]	The Child and Adolescent Twin Study in Sweden (CATSS)	Middle Childhood & Adolescence	Sweden
Taylor et. al. (2019)[11]	The Child and Adolescent Twin Study in Sweden (CATSS)	Middle Childhood & Adolescence	Sweden
Taylor et. al. (2020)[55]	The Child and Adolescent Twin Study in Sweden (CATSS)	Middle Childhood & Adolescence	Sweden
Taylor, Charman & Ronald (2015)[56]	Twins Early Development Study (TEDS)	Adolescence	United Kingdom
Tick et. al. (2016)[57]	Twins Early Development Study (TEDS)	Middle Childhood	United Kingdom
Towers et. al. (2000)[58]	The Nonshared Environment in Adolescent Development (NEAD)	Adolescence	United States
Trzaskowski, Dale & Plomin (2013)[19]	Twins Early Development Study (TEDS)	Adolescence	United Kingdom

Yip et. al. (2018)[59]	Swedish Medical Register, Multi-Generation Register	Childhood	Sweden
Hallmayer et. al. (2011)[60]	California Autism Twins Study	Adolescence	United States
Lundstrom et. al. (2011)[61]	The Child and Adolescent Twin Study in Sweden (CATSS)	Middle Childhood & Adolescence	Sweden
Taniai et. al. (2008)[62]	Nagoya North District Care Center for Disabled Children, Nagoya Child Welfare Center, and Nagoya West District Care Center for Disabled Children	Childhood & Adolescence	Japan
Lundstrom et. al. (2010)[63]	The Child and Adolescent Twin Study in Sweden (CATSS)	Middle Childhood & Adolescence	Sweden
Colvert et. al. (2015)[64]	Twins Early Development Study (TEDS)	Middle Childhood	United Kingdom
Ronald, Happe & Plomin (2005)[65]	Twins Early Development Study (TEDS)	Childhood	United Kingdom
**Heritability and environmental influences on ADHD**			
Boomsma, Van Beijsterveldt, Odinstova, Neale & Dolan (2020)[66]	The Young Netherlands Twin Register (YNTR)	Middle Childhood	Netherlands
Brikell et. al. (2016)[67]	Twin Study of Child and Adolescent Development (TCHAD)	Middle Childhood	Sweden
Brooker et. al. (2020)[68]	Wisconsin Twin Panel	Adolescence	United States
Burt, Krueger, McGue & Iacono (2001)[69]	The Minnesota Twin Family Study (MTFS)	Middle Childhood & Adolescence	United States
Burt, Larsson, Lichtenstein & Klump (2012)[70]	The Michigan State University Twin Registry	Childhood & Middle Childhood	United States
Chang, Lichtenstein & Larsson (2012)[71]	Twin Study of Child and Adolescent Development (TCHAD)	Middle Childhood & Adolescence	Sweden
Chang, Lichtenstein, Asherson & Larsson (2013)[72]	Twin Study of Child and Adolescent Development (TCHAD)	Middle Childhood	Sweden
Cheesman et. al. (2017)[13]	Twins Early Development Study (TEDS)	Adolescence	United Kingdom
Chen et. al. (2016)[73]	Chinese Child and Adolescent Twin Register	Childhood & Adolescence	China
Cheung, Fazier-Wood, Asherson, Rijsdijk & Kuntsi (2014)[74]	Twins Early Development Study (TEDS)	Childhood & Middle Childhood	United Kingdom
Coolidge, Thede & Toung (2000)[75]	Twin study in Colorado	Middle Childhood	United States
Curran et. al. (2003)[76]	The Childhood Hyperactivity and Inattention Project (CHIP)	Childhood & Adolescence	United Kingdom

de Zeuw, van Beijsterveldt, Lubke, Glasner & Boomsma (2015)[77]	Netherlands twin register (NTR)	Childhood	Netherlands
Derks et. al. (2008)[78]	Netherlands twin register (NTR)	Childhood	Netherlands
Derks, Dolan, Hudziak, Neale & Boomsma (2007)[79]	Netherlands twin register (NTR)	Childhood	Netherlands
Derks, Hudziak, van Beijsterveldts, Dolan & Boomsma (2006)[80]	Netherlands twin register (NTR)	Childhood	Netherlands
Dick, Viken, Kaprio, Pulkkinen & Rose (2005)[81]	The Finnish Twin Cohort Study	Adolescence	Finland
Dolan, De Zeeuw, Zayats, Van Beijsterveldt & Boomsma (2020)[82]	Netherlands twin register (NTR)	Adolescence	Netherlands
Du Rietz et. al. (2021)[10]	Medical Birth Register, Multi-Generation Register	Childhood & Adolescence	Sweden
Ebejer et. al. (2010)[83]	Australian Twin Register, Colorado Birth Registry, and Medical Birth Registries in Norway and Sweden	Childhood	Australia, United States, Norway, Sweden
Ebejer et. al. (2015)[84]	The Brisbane Longitudinal Twin Study	Middle Childhood & Adolescence	Australia
Edelbrock, Rende, Plomin & Thompson (1995)[85]	Western reserve twin project (WRTP)	Childhood & Adolescence	United States
Gould, Coventry, Olson & Byrne (2018)[86]	National Assessment Program in Numeracy and Literacy (NAPLAN)	Childhood & Adolescence	Australia
Greven, Asherson, Rijsdijk & Plomin (2011)[87]	Twins Early Development Study (TEDS)	Middle Childhood	United Kingdom
Greven, Harlaar, Dale & Plomin (2011)[88]	Twins Early Development Study (TEDS)	Adolescence	United Kingdom
Greven, Kovas, Willcutt, Petrill & Plomin (2014)[89]	Twins Early Development Study (TEDS)	Adolescence	United Kingdom
Greven, Rijsdijk, Asherson & Plomin (2012)[90]	Twins Early Development Study (TEDS)	Middle Childhood	United Kingdom
Greven, Rijsdijk, Plomin (2011)[91]	Twins Early Development Study (TEDS)	Middle Childhood	United Kingdom
Hay, Bennett, Levy, Sergeant & Swanson (2007)[92]	The Australian Twin ADHD Project (ATAP)	Childhood & Middle Childhood	Australia

Heutink, Verhuls & Boomsma (2006)[93]	Netherlands twin register (NTR)	Childhood	Netherlands
Hudziak, Derks, Althoff, Rettew & Boomsma (2005)[94]	Netherlands twin register (NTR)	Childhood	Netherlands
Hur (2014)[95]	The South Korean Twin Registry (SKTR)	Childhood	South Korea
Jaffee, Hanscombe, Haworth, Davis & Plomin (2012)[96]	Twins Early Development Study (TEDS)	Middle Childhood	United Kingdom
Johnson, McGue & Iacono (2005)[97]	The Minnesota Twin Family Study (MTFS)	Adolescence	United States
Kan et. al. (2013)[98]	Netherlands twin register (NTR)	Childhood & Middle Childhood	Netherlands
Kan, van Beijsterveldt, Bartels & Boomsma (2014)[99]	Netherlands twin register (NTR)	Adolescence	Netherlands
Kuja-Halkola, Lichtenstein, D'Onforio & Larsson (2015)[100]	Twin Study of Child and Adolescent Development (TCHAD)	Middle Childhood	Sweden
Kuntsi & Stevenson (2001)[101]	Twin study in Southern England	Childhood & Adolescence	United Kingdom
Kuntsi et. al. (2014)[102]	Twins Early Development Study (TEDS)	Middle Childhood	United Kingdom
Kuntsi, Gayan & Stevenson (2000)[103]	Twins Early Development Study (TEDS)	Childhood & Adolescence	United Kingdom
Kuntsi, Rijsdijk, Ronald, Asherson & Plomin (2005)[104]	Twins Early Development Study (TEDS)	Childhood	United Kingdom
Larsson, Anckarsater, Rastam, Chang & Lichtenstein (2012)[105]	Swedish Twin Register	Middle Childhood & Adolescence	Sweden
Larsson, Dilshad, Lichtenstein & Barker (2011)[106]	Twin Study of Child and Adolescent Development (TCHAD)	Middle Childhood & Adolescence	Sweden
Lemery-Chalfant, Doelger & Goldsmith (2008)[107]	Wisconsin Twin Panel	Middle Childhood	United States
Levy, Hay, McStephen, Wood & Waldman (1997)[108]	The Australian Twin ADHD Project (ATAP)	Childhood & Adolescence	Australia
Lewis & Plomin (2015)[109]	Twins Early Development Study (TEDS)	Childhood	United Kingdom
Lewis, Haworth & Plomin (2014)[110]	Twins Early Development Study (TEDS)	Adolescence	United Kingdom

Lichtenstein, Carlstrom, Rastam, Gillberg & Anckarsater (2010)[1]	Swedish Twin Register	Middle Childhood	Sweden
Lifford, Harold & Thapar (2009)[111]	The Cardiff Study of All Wales and Northwest of England Twins (CaStANET), South Wales Family Study (SWFS)	Adolescence	United Kingdom
Little, Hart, Schatschneider & Taylor (2016)[112]	Florida Twin Project on Behavior and Environment (FTP-BE)	Adolescence	United States
LoParo & Waldman (2014)[113]	Twin study in Georgia	Middle Childhood	United States
Martin, Piek & Hay (2006)[114]	The Australian Twin ADHD Project (ATAP)	Childhood & Adolescence	Australia
McLoughlin, Ronald, Kuntsi, Asherson & Plomin (2007)[115]	Twins Early Development Study (TEDS)	Childhood & Middle Childhood	United Kingdom
Merwood et. al. (2013)[116]	Twins Early Development Study (TEDS)	Adolescence	United Kingdom
Michelini, Eley, Gregory & McAdams (2015)[117]	The Genesis 12-19 (G1219) Study	Adolescence	United Kingdom
Mikolajewski, Allan, Hart, Lonigan & Taylor (2013)[118]	The Florida Twin Project on Reading (FTP-R)	Childhood & Adolescence	United States
Molenaar, Middeldorp, van Beijsterveldt & Boomsma (2015)[119]	Netherlands twin register (NTR)	Childhood	Netherlands
Moruzzi, Rijsdijk & Battaglia (2014)[120]	Twin study in Italy	Middle Childhood & Adolescence	Italy
Nikolas, Klump & Burt (2015)[121]	The Michigan State University Twin Registry (MSUTR)	Childhood & Adolescence	United States
Niv, Tuvblad, Raine, Wang & Baker (2012)[122]	Southern California Twin Project	Adolescence	United States
Paloyelis, Rijsdijk, Wood, Asherson & Kuntsi (2010)[123]	Twins Early Development Study (TEDS)	Middle Childhood	United Kingdom
Peng et. al. (2016)[124]	Missouri Twin Study	Adolescence	United States
Pingault et. al. (2015)[125]	Twins Early Development Study (TEDS)	Middle Childhood	United Kingdom
Pinto, Rijsdijk, Ronald, Asherson & Kuntsi (2016)[44]	Twins Early Development Study (TEDS)	Middle Childhood	United Kingdom
Plourde et. al. (2015)[126]	The Quebec Newborn Twin Study (QNTS)	Childhood & Middle Childhood	Canada

Plourde, Boivin, Brendgen, Vitaro & Dionne (2017)[127]	The Quebec Newborn Twin Study (QNTS)	Adolescence	Canada
Polderman et. al. (2011)[128]	Netherlands twin register (NTR)	Childhood	Netherlands
Polderman, Posthuma, De Sonnerville, Verlhulst & Boomsma (2006)[45]	Netherlands twin register (NTR)	Childhood	Netherlands
Polderman, van Dongen & Boomsma (2011)[129]	Netherlands twin register (NTR)	Adolescence	Netherlands
Price et. al. (2005)[130]	Twins Early Development Study (TEDS)	Childhood	United Kingdom
Quinn et. al. (2016)[131]	The Child and Adolescent Twin Study in Sweden (CATSS)	Middle Childhood & Adolescence	Sweden
Ronald, Larsson, Anckarsater & Lichtenstein (2014)[50]	The Child and Adolescent Twin Study in Sweden (CATSS)	Middle Childhood & Adolescence	Sweden
Ronald, Simonoff, Kuntsi, Asherson & Plomin (2008)[51]	Twins Early Development Study (TEDS)	Middle Childhood	United Kingdom
Rosenberg, Pennington, Willcut & Olson (2012)[132]	Colorado Learning Disabilities Research Center	Middle Childhood & Adolescence	United States
Rydell, Taylor & Larsson (2017)[133]	Preschool Twin Study in Sweden (PETSS)	Childhood	Sweden
Saudino & Plomin (2007)[134]	Twins Early Development Study (TEDS)	Childhood	United Kingdom
Saunders et. al. (2019)[135]	The Child and Adolescent Twin Study in Sweden (CATSS)	Middle Childhood & Adolescence	Sweden
Siebelink et. al. (2019)[136]	Twins Early Development Study (TEDS)	Adolescence	United Kingdom
Simonoff et. al. (1998)[137]	Virginia twin study of adolescent behavioral development (VTSABD)	Adolescence	United States
Stern et. al. (2020)[138]	E-RISK	Childhood	United Kingdom
Stevenson (1992)[139]	Twin study in London	Adolescence	United Kingdom
Stevenson, Pennington, Gilger, DeFries & Gillis (1993)[140]	Twin study in London	Adolescence	United Kingdom
Taylor et. al. (2019)[11]	The Child and Adolescent Twin Study in Sweden (CATSS)	Middle Childhood & Adolescence	Sweden
Taylor, Allan, Mikolajewski & Hart (2013)[141]	The Florida Twin Project on Reading (FTP-R)	Childhood & Adolescence	United States
Taylor, Charman & Ronald (2015)[56]	Twins Early Development Study (TEDS)	Adolescence	United Kingdom

Thapar, Hervas & McGuffin (1995)[142]	The Cardiff Births Survey (CBS)	Middle Childhood & Adolescence	United Kingdom
Towers et. al. (2000)[58]	The Nonshared Environment in Adolescent Development (NEAD)	Adolescence	United States
Trzaskowski, Dale & Plomin (2013)[33]	Twins Early Development Study (TEDS)	Adolescence	United Kingdom
Tuvblad, Zheng, Raine & Baker (2009)[143]	UoC Twin Study of Risk Factors for Antisocial Behavior	Middle Childhood	United States
Tye et. al. (2012)[144]	Twins Early Development Study (TEDS), The Neurophysiological Study of Activity and Attention in Twins (NEAAT)	Middle Childhood & Adolescence	United Kingdom
Vendlinski et. al. (2014)[145]	Wisconsin Twin Panel	Childhood	United States
Waszczuk, Zavos & Eley (2020)[146]	Twins Early Development Study (TEDS)	Adolescence	United Kingdom
Willcutt et. al. (2007)[147]	Colorado Twin Register, Autstralian Twin Register, Medical Birth Register	Childhood	Australia, United States, Norway, Sweden
Willcutt et. al. (2010)[148]	Colorado Learning Disabilities Research Center	Middle Childhood & Adolescence	United States
Wood, Rijsdijk, Asherson & Kuntsi (2009)[149]	Twins Early Development Study (TEDS)	Middle Childhood	United Kingdom
Wood, Rijsdijk, Asherson & Kuntsi (2011)[150]	Twins Early Development Study (TEDS)	Middle Childhood	United Kingdom
Wood, Rijsdijk, Saudino, Asherson & Kuntsi (2008)[151]	Twins Early Development Study (TEDS)	Middle Childhood	United Kingdom
Zheng, Pingault, Unger & Rijsdijk (2020)[152]	Qingdao Twin Registry (QTR)	Adolescence	China
Zumberge, Baker & Manis (2007)[153]	The Southern California Twin register	Middle Childhood	United States
Burt, McGue, Krueger & Iacono (2005)[154]	The Minnesota Twin Family Study (MTFS)	Middle Childhood & Adolescence	United States
Chen et. al. (2017)[155]	Medical Birth Register, The Swedish Twin Register, The Multi-Generation Register	Childhood & Adolescence	Sweden
Crosbie et. al. (2013)[156]	Ontario Science Centre (OSC)	Childhood	Canada
Eilertsen et. al. (2018)[157]	The Norwegian mother and child cohort study (MoBa)	Childhood	Norway
Fedko et. al. (2017)[158]	Netherlands twin register (NTR)	Middle Childhood	Netherlands

Haberstick et. al. (2008)[159]	National Longitudinal Study of Adolescent Health	Childhood & Adolescence	United States
Lundstrom et. al. (2011)[61]	The Child and Adolescent Twin Study in Sweden (CATSS)	Middle Childhood & Adolescence	Sweden
Martin, Levy, Pieka & Hay (2006)[160]	The Australian Twin ADHD Project (ATAP)	Childhood & Adolescence	Australia
Merwood, Asherson & Larsson (2013)[161]	Twin Study of Child and Adolescent Development (TCHAD)	Adolescence	Sweden
Mogensen, Larsson, Lundholm & Almqvist (2011)[162]	Twin Study of Child and Adolescent Development (TCHAD)	Adolescence	Sweden
Nadder, Silberg, Eaves, Maes & Meyer (1998)[163]	Virginia twin study of adolescent behavioral development (VTSABD)	Childhood & Adolescence	United States
Rhee, Waldman, Hay & Levy (1999)[164]	Australian Twin Register	Childhood & Adolescence	Australia
Rimfeld et. al. (2021)[165]	Twins Early Development Study (TEDS)	Adolescence	United Kingdom
Singh & Waldman (2010)[166]	Georgia Twin Register	Childhood & Adolescence	United States
Willcutt, Pennington & DeFries (2000)[167]	Colorado Learning Disabilities Research Center	Middle Childhood & Adolescence	United States
Willcutt, Pennington, Olson & DeFries (2007)[168]	Colorado Learning Disabilities Research Center	Middle Childhood & Adolescence	United States
Merwood et. al. (2014)[169]	The Cardiff Study of All Wales and Northwest of England Twins (CaStANET)	Childhood & Adolescence	United Kingdom
Thapar, Harringnton, Ross & McGuffin (2000)[170]	The Greater Manchester Twin Register	Childhood & Adolescence	United Kingdom
Ehringer, Rhee, Young, Corley & Hewitt (2006)[171]	Colorado Twin Register	Adolescence	United States
Smith et. al. (2011)[172]	Center for AntisocialDrug Dependence (CADD)	Adolescence	United States
Thapar, Harringnton & McGuffin (2001)[173]	The Greater Manchester Twin Register	Childhood & Adolescence	United Kingdom
Martin, Scourfield & McGuffin (2002)[174]	Twin study in South Wales	Childhood & Adolescence	United Kingdom

Heritability and environmental influences on specific learning disorders			
Alarcon, DeFries, Light & Pennington (1997)[175]	Colorado Learning Disabilities Research Center	Middle Childhood & Adolescence	United States
Bishop (2001)[176]	Local United Kingdom sample	Childhood & Adolescence	United Kingdom
Cheesman et. al. (2017)[13]	Twins Early Development Study (TEDS)	Adolescence	United Kingdom
Cheung, Fazier-Wood, Asherson, Rijsdijk & Kuntsi (2014)[74]	Twins Early Development Study (TEDS)	Childhood & Middle Childhood	United Kingdom
Davis et. al. (2001)[177]	Colorado Twin Study of Reading Disability	Middle Childhood & Adolescence	United States
Davis et. al. (2008)[178]	Twins Early Development Study (TEDS)	Middle Childhood	United Kingdom
Davis et. al. (2014)[179]	Twins Early Development Study (TEDS), Avon Longitudinal Study of Parents and Children (ALSPAC)	Adolescence	United Kingdom
DeFries & Alarcon (1996)[180]	Colorado Learning Disabilities Research Center	Middle Childhood & Adolescence	United States
DeFries, Knopik & Wadsworth (1999)[181]	Colorado Learning Disabilities Research Center	Middle Childhood & Adolescence	United States
Ebejer et. al. (2010)[83]	Australian Twin Register, Colorado Birth Registry, Medical Birth Registries in Norway and Sweden	Middle Childhood	Australia, United States, Norway, Sweden
Erbeli, Hart, Wagner & Taylor (2018)[182]	The Florida Twin Project on Reading (FTP-R)	Childhood & Adolescence	United States
Erbeli, Hart & Taylor (2019)[183]	Florida Twin Project on Behavior and Environment (FTP-BE)	Middle Childhood	United States
Gayan & Olson (2001)[184]	Colorado Learning Disabilities Research Center	Middle Childhood & Adolescence	United States
Greven, Harlaar, Dale & Plomin (2011)[88]	Twins Early Development Study (TEDS)	Adolescence	United Kingdom
Greven, Kovas, Willcutt, Petrill & Plomin (2014)[89]	Twins Early Development Study (TEDS)	Adolescence	United Kingdom
Greven, Rijsdijk, Asherson & Plomin (2012)[90]	Twins Early Development Study (TEDS)	Middle Childhood	United Kingdom

Harlaar, Kovas, Dale, Petrill & Plomin (2012)[185]	Twins Early Development Study (TEDS)	Adolescence	United Kingdom
Harlaar, Trzaskowski, Dale & Plomin (2014)[186]	Twins Early Development Study (TEDS)	Childhood	United Kingdom
Hart, Petrill, Thompson & Plomin (2009)[187]	Western reserve twin project (WRTP)	Childhood & Middle Childhood	United States
Hensler, Schatschneider, Taylor & Wagner (2010)[188]	The Florida Twin Project on Reading (FTP-R)	Childhood	United States
Hohnen & Stevenson (1999)[17]	Twin study in London	Childhood	United Kingdom
Kovas et. al. (2007)[189]	Twins Early Development Study (TEDS)	Childhood	United Kingdom
Little, Hart, Schatschneider & Taylor (2016)[112]	Florida Twin Project on Behavior and Environment (FTP-BE)	Adolescence	United States
Marlow et. al. (2001)[190]	Twin study in Reading	Childhood & Adolescence	United Kingdom
Newsome, Boisvert & Wright (2014)[191]	The Early Childhood Longitudinal Study (ECLS)	Childhood	United States
Olson, Gillis, Rack, DeFries & Fulker (1991)[192]	Colorado Reading Project	Childhood & Adolescence	United States
Paloyelis, Rijsdijk, Wood, Asherson & Kuntsi (2010)[123]	Twins Early Development Study (TEDS)	Middle Childhood	United Kingdom
Petrill et. al. (2007)[193]	Western reserve twin project (WRTP)	Childhood & Middle Childhood	United States
Plourde et. al. (2015)[126]	The Quebec Newborn Twin Study (QNTS)	Childhood & Middle Childhood	Canada
Plourde, Boivin, Brendgen, Vitaro & Dionne (2017)[127]	The Quebec Newborn Twin Study (QNTS)	Adolescence	Canada
Polderman et. al. (2011)[128]	Netherlands twin register (NTR)	Middle Childhood	Netherlands
Rosenberg, Pennington, Willcut & Olson (2012)[132]	Colorado Learning Disabilities Research Center	Middle Childhood & Adolescence	United States
Samuelsson et. al. (2007)[194]	Colorado Twin Register	Childhood	Australia
Taylor et. al. (2019)[11]	The Child and Adolescent Twin Study in Sweden (CATSS)	Middle Childhood & Adolescence	Sweden

Tosto et. al. (2014)[195]	Twins Early Development Study (TEDS)	Adolescence	United Kingdom
Trzaskowski, Dale & Plomin (2013)[19]	Twins Early Development Study (TEDS)	Adolescence	United Kingdom
Wadsworth, DeFries, Willcutt, Pennington & Olson (2015)[196]	Colorado Learning Disabilities Research Center	Childhood & Adolescence	United States
Wadsworth, DeFries, Willcutt, Pennington & Olson (2016)[197]	Longitudinal Twin Study of Early Reading Development	Middle Childhood & Adolescence	United States
Wadsworth, Olson & DeFries (2010)[198]	Colorado Reading Project, Colorado Learning Disabilities Research Center	Middle Childhood & Adolescence	United States
Wadsworth, Olson, Penningtom & DeFries (2000)[199]	Colorado Reading Project, Colorado Learning Disabilities Research Center	Middle Childhood & Adolescence	United States
Willcutt et. al. (2010)[148]	Colorado Learning Disabilities Research Center	Middle Childhood & Adolescence	United States
Willcutt et. al. (2019)[200]	Colorado Learning Disabilities Research Center	Middle Childhood & Adolescence	United States
Willcutt, Pennington & DeFries (2000)[201]	Colorado Learning Disabilities Research Center	Middle Childhood & Adolescence	United States
Zumberge, Baker & Manis (2007)	The Southern California Twin register	Middle Childhood	United States
Astrom, Wadsworth, Olson, Willcutt & DeFries (2011)[202]	Colorado Learning Disabilities Research Center	Middle Childhood	United States
Betjemann et. al. (2010)[203]	Colorado Learning Disabilities Research Center	Middle Childhood & Adolescence	United States
Bishop, Adams & Norbury (2004)[204]	Twins Early Development Study (TEDS)	Childhood	United Kingdom
Castles, Datta, Gayan & Olson (1999)	Colorado Learning Disabilities Research Center	Middle Childhood & Adolescence	United States
Christopher et. al. (2013)[206]	International Longitudinal Twin Study (ILTS)	Childhood	United States
Daucourt, Haughbrook, Van Bergen & Hart (2020)[207]	Florida Twin Project on Behavior and Environment (FTP-BE)	Childhood & Adolescence	United States
DeFries, Fulker & LaBuda (1987)[208]	Colorado Reading Project	Adolescence	United States
Erbeli, Hart & Taylor (2018)[209]	The Florida Twin Project on Reading (FTP-R)	Childhood	United States
Friend et. al. (2009)[210]	Colorado Twin Register	Childhood	United States

Friend, DeFries, Wadsworth & Olson (2007)[211]	Colorado Learning Disabilities Research Center	Middle Childhood	United States
Garon-Carrier et. al. (2017)[212]	The Quebec Newborn Twin Study (QNTS)	Childhood	Canada
Gayan & Olson (2003)[213]	Colorado Learning Disabilities Research Center	Middle Childhood & Adolescence	United States
Gillis, DeFries & Fulker (1992)[214]	Colorado Reading Project	Childhood & Adolescence	United States
Grasby & Coventry (2016)[215]	Australian Twin Register	Middle Childhood	Australia
Harlaar, Dale & Plomin (2007)[216]	Twins Early Development Study (TEDS)	Childhood	United Kingdom
Hart et. al. (2013)[217]	The Florida Twin Project on Reading (FTP-R)	Childhood	United States
Hawke, Stallings, Wadsworth & DeFries (2008)[218]	Colorado Reading Project, Colorado Learning Disabilities Research Center	Middle Childhood & Adolescence	United States
Knopik et. al. (2002)[219]	Colorado Learning Disabilities Research Center	Middle Childhood & Adolescence	United States
Knopik, Alarcon & DeFries (1997)[220]	Colorado Learning Disabilities Research Center	Middle Childhood & Adolescence	United States
Kovas et. al. (2013)[221]	Twins Early Development Study (TEDS)	Childhood	United Kingdom
Kovas, Haworth, Harlaar, Petrill, Dale & Plomin (2007)[222]	Twins Early Development Study (TEDS)	Middle Childhood	United Kingdom
Lazaroo et. al. (2019)[223]	Brisbane Longitudinal Twin Study	Adolescence	Australia
Logan et. al. (2013)[224]	Western reserve twin project (WRTP)	Childhood & Adolescence	United States
Malanchini et. al. (2017)[225]	Twins Early Development Study (TEDS)	Middle Childhood	United Kingdom
Malanchini et. al. (2020)[226]	Twins Early Development Study (TEDS)	Adolescence	United Kingdom
Malanchini, Engelhardt, Grotzinger, Harden & Tucker-Drob (2019)[227]	Texas Twin Project	Middle Childhood & Adolescence	United States
Martin, Levy, Pieka & Hay (2006)[160]	The Australian Twin ADHD Project (ATAP)	Childhood & Adolescence	Australia
Oliver, Dale & Plomin (2007)[228]	Twins Early Development Study (TEDS)	Childhood & Middle Childhood	United Kingdom

Petrill et. al. (2010)[229]	Western reserve twin project (WRTP)	Childhood & Middle Childhood	United States
Rimfeld et. al. (2018)[230]	Twins Early Development Study (TEDS)	Childhood	United Kingdom
Rimfeld et. al. (2019)[231]	Twins Early Development Study (TEDS)	Childhood	United Kingdom
Rimfeld, Ayorech, Dale, Kovas & Plomin (2016)[232]	Twins Early Development Study (TEDS)	Adolescence	United Kingdom
Rimfeld, Kovas, Dale & Plomin (2015)[233]	Twins Early Development Study (TEDS)	Adolescence	United Kingdom
Shakeshaft et. al. (2013)[234]	Twins Early Development Study (TEDS)	Adolescence	United Kingdom
Swagerman et. al. (2017)[235]	Netherlands twin register (NTR)	Middle Childhood	Netherlands
Taylor & Schatschneider (2010)[236]	The Florida Twin Project on Reading (FTP-R)	Childhood	United States
Taylor, Erbeli, Hart & Johnson (2020)[237]	The Florida Twin Project on Reading (FTP-R)	Adolescence	United States
Tosto et. al. (2017)[32]	Twins Early Development Study (TEDS)	Childhood	United Kingdom
Tosto et. al. (2019)[238]	Western reserve twin project (WRTP)	Middle Childhood & Adolescence	United States
Tosto, Malykh, Voronin, Plomin & Kovas (2013)[239]	Twins Early Development Study (TEDS)	Adolescence	United Kingdom
Trzaskowski et. al. (2013)[19]	Twins Early Development Study (TEDS)	Adolescence	United Kingdom
Wadsworth, Olson, Willcutt & DeFries (2012)[240]	Colorado Reading Project	Middle Childhood & Adolescence	United States
Willcutt, Pennington, Olson & DeFries (2007)[168]	Colorado Learning Disabilities Research Center	Middle Childhood & Adolescence	United States
Wong, Chow, Ho, Waye & Bishop (2014)[241]	Chinese Twin Study of Reading Development	Childhood & Adolescence	China
Keenan et. al. (2006)[242]	Colorado Learning Disabilities Research Center	Middle Childhood & Adolescence	United States
**Heritability and environmental influences on motor disorders**			
Du Rietz et. al. (2021)[10]	Medical Birth Register, Multi-Generation Register	Childhood & Adolescence	Sweden

Lichtenstein, Carlstrom, Rastam, Gillberg & Anckarsater (2010)[1]	Swedish Twin Register	Middle Childhood	Sweden
Martin, Piek & Hay (2006)[114]	The Australian Twin ADHD Project (ATAP)	Childhood & Adolescence	Australia
Molenaar, Middeldorp, van Beijsterveldt & Boomsma (2015)[119]	Netherlands twin register (NTR)	Childhood	Netherlands
Taylor et. al. (2019)01/08/2025 16:56:00	The Child and Adolescent Twin Study in Sweden (CATSS)	Middle Childhood & Adolescence	Sweden
Bishop (2002)[21]	Twin study in the United Kingdom	Childhood & Adolescence	United Kingdom
Mataix-Cols et. al. (2015)[243]	Multi-Generation Register, National Patient Register	Childhood & Adolescence	Sweden
Fliers et. al. (2009)[244]	International Multicenter ADHD Genetics Study	Adolescence	Netherlands
**Genetic and environmental overlap between ASD & ADHD**			
Lichtenstein, Carlstrom, Rastam, Gillberg & Anckarsater (2010)[1]	Swedish Twin Register	Middle childhood & Adolescence	Sweden
Lundstrom et. al. (2011)[61]	The Child and Adolescent Twin Study in Sweden (CATSS)	Middle childhood & Adolescence	Sweden
Pinto, Rijsdijk, Ronald, Asherson & Kuntsi (2016)[44]	Twins Early Development Study (TEDS)	Middle childhood	United Kingdom
Ronald, Larsson, Anckarsater & Lichtenstein (2014)[50]	The Child and Adolescent Twin Study in Sweden (CATSS)	Middle childhood	Sweden
Taylor et. al. (2013)[245]	Twins Early Development Study (TEDS)	Middle childhood	United Kingdom
Taylor, Charman & Ronald (2015)[56]	Twins Early Development Study (TEDS)	Adolescence	United Kingdom
**Genetic and environmental overlap between ADHD & motor disorders**			
Lichtenstein, Carlstrom, Rastam, Gillberg & Anckarsater (2010)[1]	Swedish Twin Register	Middle childhood & Adolescence	Sweden
Martin, Piek & Hay (2006)[114]	The Australian Twin ADHD Project (ATAP)	Childhood & Adolescence	Australia
**Genetic and environmental overlap between ADHD & specific learning disorders**			
Cheung, Fazier-Wood, Asherson, Rijsdijk & Kuntsi (2014)[74]	Twins Early Development Study (TEDS)	Childhood & Middle Childhood	United Kingdom

Greven, Harlaar, Dale & Plomin (2011)[88]	Twins Early Development Study (TEDS)	Adolescence	United Kingdom
Greven, Kovas, Willcutt, Petrill & Plomin (2014)[89]	Twins Early Development Study (TEDS)	Adolescence	United Kingdom
Greven, Rijsdijk, Asherson & Plomin (2012)[90]	Twins Early Development Study (TEDS)	Middle childhood	United Kingdom
Lichtenstein, Carlstrom, Rastam, Gillberg & Anckarsater (2010)[1]	Swedish Twin Register	Middle childhood & Adolescence	Sweden
Light, Pennington, Gilger & DeFries (1995)[246]	Colorado Reading Project	Middle childhood & Adolescence	United States
Paloyelis, Rijsdijk, Wood, Asherson & Kuntsi (2010)[123]	Twins Early Development Study (TEDS)	Middle childhood	United Kingdom
Plourde et. al. (2015)[126]	The Quebec Newborn Twin Study (QNTS)	Childhood & Middle Childhood	Canada
Plourde, Boivin, Brendgen, Vitaro & Dionne (2017)[127]	The Quebec Newborn Twin Study (QNTS)	Adolescence	Canada
Polderman et. al. (2011)[128]	Netherlands twin register (NTR)	Childhood & Middle Childhood	Netherlands
Rosenberg, Pennington, Willcut & Olson (2012)[132]	Colorado Learning Disabilities Research Center	Middle childhood & Adolescence	United States
Stevenson, Pennington, Gilger, DeFries & Gillis (1993)[140]	Twin study in London	Adolescence	United Kingdom
Wadsworth, DeFries, Willcutt, Pennington & Olson (2015)[196]	Colorado Learning Disabilities Research Center	Childhood & Adolescence	United States
Wadsworth, DeFries, Willcutt, Pennington & Olson (2016)[197]	Longitudinal Twin Study of Early Reading Development	Middle childhood & Adolescence	United States
Willcutt et. al. (2010)[148]	Colorado Learning Disabilities Research Center	Middle childhood & Adolescence	United States
Willcutt, Pennington & DeFries (2000)[201]	Colorado Learning Disabilities Research Center	Middle childhood & Adolescence	United States
Willcutt, Pennington, Olson & DeFries (2007)[168]	Colorado Learning Disabilities Research Center	Middle childhood & Adolescence	United States

Martin, Levy, Pieka & Hay (2006)[160]	The Australian Twin ADHD Project (ATAP)	Childhood & Adolescence	Australia
**Genetic and environmental overlap between ASD & communication disorders***			
Dworzynski et. al. (2008)[37]	Twins Early Development Study (TEDS)	Childhood & Middle Childhood	United Kingdom
**Genetic and environmental overlap between ASD & motor disorders***			
Lichtenstein, Carlstrom, Rastam, Gillberg & Anckarsater (2010)[1]	Swedish Twin Register	Middle childhood & Adolescence	Sweden
**Genetic and environmental overlap between ASD & specific learning disorders***			
Lichtenstein, Carlstrom, Rastam, Gillberg & Anckarsater (2010)[1]	Swedish Twin Register	Middle childhood & Adolescence	Sweden
**Genetic and environmental overlap between motor disorders & specific learning disorders***			
Lichtenstein, Carlstrom, Rastam, Gillberg & Anckarsater (2010)[1]	Swedish Twin Register	Middle childhood & Adolescence	Sweden
**Genetic and environmental overlap between communication disorders & motor disorders**			
Bishop (2002)[21]	Twin study in the United Kingdom	Childhood & Adolescence	United Kingdom
Ooki (2005)[247]	Twin study in Japan	Childhood & Adolescence	Japan
**Genetic and environmental overlap between communication disorders & specific learning disorders**			
Bishop (2001)[176]	Local United Kingdom sample	Childhood & Adolescence	United Kingdom
Tosto et. al. (2017)[32]	Twins Early Development Study (TEDS)	Childhood	United Kingdom
**Genetic and environmental overlap between subtypes of specific learning disorders**			
Davis et. al. (2008)[178]	Twins Early Development Study (TEDS)	Middle childhood	United Kingdom
Davis et. al. (2014)[179]	Twins Early Development Study (TEDS), Avon Longitudinal Study of Parents and Children (ALSPAC)	Adolescence	United Kingdom
Greven, Kovas, Willcutt, Petrill & Plomin (2014)[89]	Twins Early Development Study (TEDS)	Adolescence	United Kingdom
Harlaar, Kovas, Dale, Petrill & Plomin (2012)[185]	Twins Early Development Study (TEDS)	Adolescence	United Kingdom

Willcutt et. al. (2019)[200]	Colorado Learning Disabilities Research Center	Middle childhood & Adolescence	United States
Gillis, DeFries & Fulker (1992)[214]	Colorado Reading Project	Childhood & Adolescence	United States
Knopik, Alarcon & DeFries (1997)[220]	Colorado Learning Disabilities Research Center	Middle childhood & Adolescence	United States
Kovas, Haworth, Harlaar, Petrill, Dale & Plomin (2007)[222]	Twins Early Development Study (TEDS)	Middle childhood	United Kingdom
Oliver, Dale & Plomin (2007)[228]	Twins Early Development Study (TEDS)	Childhood & Middle childhood	United Kingdom
**Genetic and environmental overlap between ADHD & conduct disorder**			
Burt, Krueger, McGue & Iacono (2001)[69]	The Minnesota Twin Family Study (MTFS)	Middle childhood & Adolescence	United States
Dick, Viken, Kaprio, Pulkkinen & Rose (2005)[81]	The Finnish Twin Cohort Study	Adolescence	Finland
Tuvblad, Zheng, Raine & Baker (2009)[143]	The Southern California Twin register	Middle childhood	United States
Hur (2015)[248]	The South Korean Twin Registry (SKTR)	Childhood & Adolescence	South Korea
Martin, Levy, Pieka & Hay (2006)[160]	The Australian Twin ADHD Project (ATAP)	Childhood & Adolescence	Australia
Coolidge, Thede & Toung (2000)[75]	Twin study in Colorado	Middle childhood	United States
**Genetic and environmental overlap between ADHD & oppositional defiant disorder**			
Burt, Krueger, McGue & Iacono (2001)[69]	The Minnesota Twin Family Study (MTFS)	Middle childhood & Adolescence	United States
Dick, Viken, Kaprio, Pulkkinen & Rose (2005)[81]	The Finnish Twin Cohort Study	Adolescence	Finland
Tuvblad, Zheng, Raine & Baker (2009)[143]	The Southern California Twin register	Middle childhood	United States
Wood, Rijsdijk, Asherson & Kuntsi (2009)[149]	Twins Early Development Study (TEDS)	Middle childhood	United Kingdom

Martin, Levy, Pieka & Hay (2006)[160]	The Australian Twin ADHD Project (ATAP)	Childhood & Adolescence	Australia
Coolidge, Thede & Toung (2000)[75]	Twin study in Colorado	Middle childhood	United States
**Genetic and environmental overlap between ASD & conduct disorder**			
Jones et. al. (2009)[42]	Twins Early Development Study (TEDS)	Middle childhood	United Kingdom
O'Nions et. al. (2015)[249]	Twins Early Development Study (TEDS)	Middle childhood	United Kingdom
**Genetic and environmental overlap between ASD & conduct disorder***			
Lundstrom et. al. (2011)[61]	The Child and Adolescent Twin Study in Sweden (CATSS)	Middle childhood & Adolescence	Sweden
**Genetic and environmental overlap between specific learning disorders & disruptive behaviour***			
Newsome, Boisvert & Wright (2014)[191]	The Early Childhood Longitudinal Study (ECLS)	Childhood	United States

Supplementary Table 32. Overview of family-based studies using male samples. Co-occurrences between disorders annotated with an asterisk (*) indicate pairs of disorders for which meta-analysis could not be performed.

Reference	Cohort	Age category	Country
**Heritability and environmental influences on communication disorders**			
Spinath, Price, Dale & Plomin (2004)[250]	Twins Early Development Study (TEDS)	Childhood	United Kingdom
Taylor et. al. (2014)[251]	Twins Early Development Study (TEDS)	Adolescence	United Kingdom
Ooki (2005)[247]	Twin study in Japan	Childhood & Adolescence	Japan
Viding et. al. (2004)[34]	Twins Early Development Study (TEDS)	Childhood	United Kingdom
**Heritability and environmental influences on ASD**			
Cheesman et. al. (2017)[13]	Twins Early Development Study (TEDS)	Adolescence	United Kingdom
Constantino & Todd (2003)[252]	Missouri Twin Study	Adolescence	United States
Frazier et. al. (2014)[39]	Interactive Autism Network (IAN)	Middle Childhood	United States
Hallett, Ronald, Rijsdijk & Happe (2012)[253]	Twins Early Development Study (TEDS)	Childhood & Middle Childhood	United Kingdom
Hoekstra, Happe, Baron-Cohen & Ronald (2010)[254]	Twins Early Development Study (TEDS)	Middle Childhood	United Kingdom
Holmboe et. al. (2014)[255]	Twins Early Development Study (TEDS)	Middle Childhood	United Kingdom
Robinson et. al. (2011)[46]	Twins Early Development Study (TEDS)	Adolescence	United Kingdom
Robinson et. al. (2012)[47]	Twins Early Development Study (TEDS)	Middle Childhood & Adolescence	United Kingdom
Ronald et. al. (2006)[48]	Twins Early Development Study (TEDS)	Middle Childhood	United Kingdom
Ronald, Larsson, Anckarsater & Lichtenstein (2014)[50]	The Child and Adolescent Twin Study in Sweden (CATSS)	Middle Childhood	Sweden

Ronald, Simonoff, Kuntsi, Asherson & Plomin (2008)[51]	Twins Early Development Study (TEDS)	Middle Childhood	United Kingdom
Scherff et. al. (2014)[52]	Twins Early Development Study (TEDS)	Adolescence	United Kingdom
Taylor et. al. (2013)[245]	Twins Early Development Study (TEDS)	Middle Childhood	United Kingdom
Taylor et. al. (2014)[251]	Twins Early Development Study (TEDS)	Adolescence	United Kingdom
Taylor et. al. (2018)[54]	The Child and Adolescent Twin Study in Sweden (CATSS)	Middle Childhood & Adolescence	Sweden
Taylor et. al. (2020)[55]	The Child and Adolescent Twin Study in Sweden (CATSS)	Middle Childhood & Adolescence	Sweden
Taylor, Gillberg, Lichtenstein & Lundstrom (2017)[256]	The Child and Adolescent Twin Study in Sweden (CATSS)	Middle Childhood & Adolescence	Sweden
Hallmayer et. al. (2011)[60]	California Autism Twins Study	Adolescence	United States
Mazefsky et. al. (2008)[257]	Autism Genetic Resource Exchange (AGRE)	Childhood & Adolescence	United States
Taniai et. al. (2008)[62]	Nagoya North District Care Center for Disabled Children, Nagoya Child Welfare Center, and Nagoya West District Care Center for Disabled Children	Childhood & Adolescence	Japan
Ronald, Happe & Plomin (2005)[65]	Twins Early Development Study (TEDS)	Childhood	United Kingdom
**Heritability and environmental influences on ADHD**			
Cheesman et. al. (2017)[13]	Twins Early Development Study (TEDS)	Adolescence	United Kingdom
Cole, Ball, Martin, Scourfield & McGuffin (2009)[258]	Cardiff Study of All Wales and North England Twins	Childhood & Adolescence	United Kingdom
Constantino, Hudziak & Todd (2003)[259]	Missouri Twin Study	Childhood & Adolescence	United States
de Zeuw, van Beijsterveldt, Lubke, Glasner & Boomsma (2015)[77]	Netherlands twin register (NTR)	Childhood	Netherlands
Dick, Viken, Kaprio, Pulkkinen & Rose (2005)[81]	The Finnish Twin Cohort Study	Adolescence	Finland

Eaves et. al. (1997)[260]	Virginia twin study of adolescent behavioral development (VTSABD)	Middle Childhood & Adolescence	United States
Eaves et. al. (2000)[261]	Virginia twin study of adolescent behavioral development (VTSABD)	Middle Childhood & Adolescence	United States
Gregory, Eley, O'Connor & Plomin (2004)[262]	Twins Early Development Study (TEDS)	Childhood	United Kingdom
Greven, Rijsdijk, Plomin (2011)[91]	Twins Early Development Study (TEDS)	Middle Childhood	United Kingdom
Hudziak, Rudiger, Neale, Heath & Todd (2000)[263]	Missouri Twin Study	Middle Childhood & Adolescence	United States
Jaffee, Hanscombe, Haworth, Davis & Plomin (2012)[96]	Twins Early Development Study (TEDS)	Middle Childhood	United Kingdom
Kuntsi, Rijsdijk, Ronald, Asherson & Plomin (2005)[104]	Twins Early Development Study (TEDS)	Middle Childhood	United Kingdom
Kuo, Lin, Yang, Soong & Chen (2004)[264]	Twin study in Taipei City	Adolescence	Taiwan
Larsson, Lichtenstein & Larsson (2006)[265]	Twin Study of Child and Adolescent Development (TCHAD)	Middle Childhood	Sweden
Lifford, Harold & Thapar (2009)[111]	The Cardiff Study of All Wales and Northwest of England Twins (CaStANET), South Wales Family Study (SWFS)	Adolescence	United Kingdom
Ronald, Larsson, Anckarsater & Lichtenstein (2014)[50]	The Child and Adolescent Twin Study in Sweden (CATSS)	Middle Childhood	Sweden
Ronald, Simonoff, Kuntsi, Asherson & Plomin (2008)[51]	Twins Early Development Study (TEDS)	Middle Childhood	United Kingdom
Rydell, Taylor & Larsson (2017)[133]	Preschool Twin Study in Sweden (PETSS)	Childhood	Sweden
Saudino & Plomin (2007)[134]	Twins Early Development Study (TEDS)	Childhood	United Kingdom
Taylor et. al. (2013)[245]	Twins Early Development Study (TEDS)	Middle Childhood	United Kingdom
van Beijsterveldt, Verhulst, Molenaar & Boomsma (2004)[266]	Netherlands twin register (NTR)	Childhood	Netherlands
Vierikko, Pulkkinen, Kaprio & Rose (2004)[267]	The Finnish Twin Cohort Study	Adolescence	Finland
Burt, McGue, Krueger & Iacono (2005)[154]	The Minnesota Twin Family Study (MTFS)	Middle Childhood & Adolescence	United States
de Zeeuw, van Beijsterveldt, Ehli, de Geus & Boomsma (2017)[268]	Netherlands twin register (NTR)	Childhood & Adolescence	Netherlands

Do et. al. (2019)[269]	Add Health	Childhood & Adolescence	United States
Larsson, Larsson & Lichtenstein (2004)[270]	Young Twins Study	Adolescence	Sweden
Nadder, Rutter, Silberg, Maes & Eaves (2002)[271]	Virginia twin study of adolescent behavioral development (VTSABD)	Middle Childhood & Adolescence	United States
Nadder, Silberg, Eaves, Maes & Meyer (1998)[163]	Virginia twin study of adolescent behavioral development (VTSABD)	Childhood & Adolescence	United States
Rietveld, Hudziak, Bartels, Van Beijsterveldt & Boomsma (2004)[272]	Netherlands twin register (NTR)	Childhood	Netherlands
Saudino, Ronald & Plomin (2005)[273]	Twins Early Development Study (TEDS)	Childhood	United Kingdom
Silberg et. al. (1996)[274]	Virginia twin study of adolescent behavioral development (VTSABD)	Middle Childhood & Adolescence	United States
Sherman, Iacono & McGue (1997)[275]	The Minnesota Twin Family Study (MTFS)	Adolescence	United States
Smith et. al. (2011)[172]	Center for Antisocial Drug Dependence (CADD)	Adolescence	United States
**Heritability and environmental influences on specific learning disorders**			
Alarcon, DeFries & Fulker (1995)[276]	Colorado Learning Disabilities Research Center	Middle Childhood & Adolescence	United States
Bates et. al. (2004)[277]	Study of melanocytic naevi (moles)	Adolescence	Australia
Eaves et. al. (1997)[260]	Virginia twin study of adolescent behavioral development (VTSABD)	Middle Childhood & Adolescence	United States
Harlaar, Spinath, Dale & Plomin (2005)[278]	Twins Early Development Study (TEDS)	Childhood	United Kingdom
Reynolds et. al. (1996)[279]	Virginia twin study of adolescent behavioral development (VTSABD)	Middle Childhood	United States
Tosto et. al. (2014)[195]	Twins Early Development Study (TEDS)	Adolescence	United Kingdom
Grasby & Coventry (2016)[215]	Australian Twin Register	Middle Childhood	Australia
Shakeshaft et. al. (2013)[234]	Twins Early Development Study (TEDS)	Adolescence	United Kingdom
Tosto et. al. (2019)[238]	Twins Early Development Study (TEDS)	Adolescence	United Kingdom

**Heritability and environmental influences on motor disorders**			
van Beijsterveldt, Verhulst, Molenaar & Boomsma (2004)[266]	Netherlands twin register (NTR)	Childhood	Netherlands
Ooki (2005)[247]	Twin study in Japan	Childhood & Adolescence	Japan
**Genetic and environmental overlap between ASD & ADHD**			
Constantino, Hudziak & Todd (2003)[259]	Missouri Twin Study	United States	BEST
Ronald, Simonoff, Kuntsi, Asherson & Plomin (2008)[51]	Twins Early Development Study (TEDS)	United Kingdom	BEST
**Genetic and environmental overlap between ADHD & conduct disorder***			
Silberg et. al. (1996)[274]	Virginia twin study of adolescent behavioral development (VTSABD)	Twin study	United States

Supplementary Table 33. Overview of family-based studies using female samples. Co-occurrences between disorders annotated with an asterisk (*) indicate pairs of disorders for which meta-analysis could not be performed.

Reference	Cohort	Age category	Country
**Heritability and environmental influences on communication disorders**			
Spinath, Price, Dale & Plomin (2004)[250]	Twins Early Development Study (TEDS)	Childhood	United Kingdom
Taylor et. al. (2014)[251]	Twins Early Development Study (TEDS)	Adolescence	United Kingdom
Ooki (2005)[247]	Twin study in Japan	Childhood & Adolescence	Japan
Viding et. al. (2004)[34]	Twins Early Development Study (TEDS)	Childhood	United Kingdom
**Heritability and environmental influences on ASD**			
Cheesman et. al. (2017)[13]	Twins Early Development Study (TEDS)	Adolescence	United Kingdom
Constantino & Todd (2003)[252]	Missouri Twin Study	Adolescence	United States
Constantino, Hudziak & Todd (2003)[259]	Missouri Twin Study	Childhood & Adolescence	United States
Frazier et. al. (2014)[39]	Interactive Autism Network (IAN)	Middle Childhood	United States
Hallett, Ronald, Rijsdijk & Happe (2012)[253]	Twins Early Development Study (TEDS)	Childhood & Middle Childhood	United Kingdom
Hoekstra, Happe, Baron-Cohen & Ronald (2010)[254]	Twins Early Development Study (TEDS)	Middle Childhood	United Kingdom
Holmboe et. al. (2014)[255]	Twins Early Development Study (TEDS)	Middle Childhood	United Kingdom
Lundstrom et. al. (2012)[43]	The Child and Adolescent Twin Study in Sweden (CATSS)	Middle Childhood & Adolescence	Sweden
Robinson et. al. (2011)[46]	Twins Early Development Study (TEDS)	Adolescence	United Kingdom

Robinson et. al. (2012)[47]	Twins Early Development Study (TEDS)	Middle Childhood & Adolescence	United Kingdom
Ronald et. al. (2006)[48]	Twins Early Development Study (TEDS)	Middle Childhood	United Kingdom
Ronald, Larsson, Anckarsater & Lichtenstein (2014)[50]	The Child and Adolescent Twin Study in Sweden (CATSS)	Middle Childhood	Sweden
Ronald, Simonoff, Kuntsi, Asherson & Plomin (2008)[51]	Twins Early Development Study (TEDS)	Middle Childhood	United Kingdom
Scherff et. al. (2014)[52]	Twins Early Development Study (TEDS)	Adolescence	United Kingdom
Taylor et. al. (2013)[245]	Twins Early Development Study (TEDS)	Middle Childhood	United Kingdom
Taylor et. al. (2014)[251]	Twins Early Development Study (TEDS)	Adolescence	United Kingdom
Taylor et. al. (2018)[54]	The Child and Adolescent Twin Study in Sweden (CATSS)	Middle Childhood & Adolescence	Sweden
Taylor et. al. (2020)[55]	The Child and Adolescent Twin Study in Sweden (CATSS)	Middle Childhood & Adolescence	Sweden
Taylor, Gillberg, Lichtenstein & Lundstrom (2017)[256]	The Child and Adolescent Twin Study in Sweden (CATSS)	Middle Childhood & Adolescence	Sweden
Hallmayer et. al. (2011)[60]	California Autism Twins Study	Adolescence	United States
Mazefsky et. al. (2008)[257]	Autism Genetic Resource Exchange (AGRE)	Childhood & Adolescence	United States
Taniai et. al. (2008)[62]	Nagoya North District Care Center for Disabled Children, Nagoya Child Welfare Center, and Nagoya West District Care Center for Disabled Children	Childhood & Adolescence	Japan
Ronald, Happe & Plomin (2005)[65]	Twins Early Development Study (TEDS)	Childhood	United Kingdom
**Heritability and environmental influences on ADHD**			

Cheesman et. al. (2017)[13]	Twins Early Development Study (TEDS)	Adolescence	United Kingdom
Cole, Ball, Martin, Scourfield & McGuffin (2009)[258]	Cardiff Study of All Wales and North England Twins	Childhood & Adolescence	United Kingdom
de Zeuw, van Beijsterveldt, Lubke, Glasner & Boomsma (2015)[77]	Netherlands twin register (NTR)	Childhood	Netherlands
Dick, Viken, Kaprio, Pulkkinen & Rose (2005)[81]	The Finnish Twin Cohort Study	Adolescence	Finland
Eaves et. al. (1997)[260]	Virginia twin study of adolescent behavioral development (VTSABD)	Middle Childhood & Adolescence	United States
Eaves et. al. (2000)[261]	Virginia twin study of adolescent behavioral development (VTSABD)	Middle Childhood & Adolescence	United States
Gregory, Eley, O'Connor & Plomin (2004)[262]	Twins Early Development Study (TEDS)	Childhood	United Kingdom
Greven, Rijsdijk, Plomin (2011)[91]	Twins Early Development Study (TEDS)	Middle Childhood	United Kingdom
Hudziak, Rudiger, Neale, Heath & Todd (2000)[263]	Missouri Twin Study	Middle Childhood & Adolescence	United States
Jaffee, Hanscombe, Haworth, Davis & Plomin (2012)[96]	Twins Early Development Study (TEDS)	Middle Childhood	United Kingdom
Kuntsi, Rijsdijk, Ronald, Asherson & Plomin (2005)[104]	Twins Early Development Study (TEDS)	Middle Childhood	United Kingdom
Kuo, Lin, Yang, Soong & Chen (2004)[264]	Twin study in Taipei City	Adolescence	Taiwan
Larsson, Lichtenstein & Larsson (2006)[265]	Twin Study of Child and Adolescent Development (TCHAD)	Middle Childhood	Sweden
Lifford, Harold & Thapar (2009)[111]	The Cardiff Study of All Wales and Northwest of England Twins (CaStANET), South Wales Family Study (SWFS)	Adolescence	United Kingdom
Ronald, Larsson, Anckarsater & Lichtenstein (2014)[50]	The Child and Adolescent Twin Study in Sweden (CATSS)	Middle Childhood	Sweden
Ronald, Simonoff, Kuntsi, Asherson & Plomin (2008)[51]	Twins Early Development Study (TEDS)	Middle Childhood	United Kingdom
Rydell, Taylor & Larsson (2017)[133]	Preschool Twin Study in Sweden (PETSS)	Childhood	Sweden

Saudino & Plomin (2007)[134]	Twins Early Development Study (TEDS)	Childhood	United Kingdom
Taylor et. al. (2013)[245]	Twins Early Development Study (TEDS)	Middle Childhood	United Kingdom
van Beijsterveldt, Verhulst, Molenaar & Boomsma (2004)[266]	Netherlands twin register (NTR)	Childhood	Netherlands
Vierikko, Pulkkinen, Kaprio & Rose (2004)[267]	The Finnish Twin Cohort Study	Adolescence	Finland
Burt, McGue, Krueger & Iacono (2005)[154]	The Minnesota Twin Family Study (MTFS)	Middle Childhood & Adolescence	United States
de Zeeuw, van Beijsterveldt, Ehli, de Geus & Boomsma (2017)[268]	Netherlands twin register (NTR)	Childhood & Adolescence	Netherlands
Do et. al. (2019)[269]	Add Health	Childhood & Adolescence	United States
Knopik, Heath, Bucholz, Madden & Waldron (2009)	Missouri Adolescent Female Twin Study cohort	Adolescence	United States
Larsson, Larsson & Lichtenstein (2004)[270]	Young Twins Study	Adolescence	Sweden
Nadder, Rutter, Silberg, Maes & Eaves (2002)[271]	Virginia twin study of adolescent behavioral development (VTSABD)	Middle Childhood & Adolescence	United States
Nadder, Silberg, Eaves, Maes & Meyer (1998)[163]	Virginia twin study of adolescent behavioral development (VTSABD)	Childhood & Adolescence	United States
Neuman et. al. (2001)[281]	Missouri Twin Study	Adolescence	United States
Rietveld, Hudziak, Bartels, Van Beijsterveldt & Boomsma (2004)[272]	Netherlands twin register (NTR)	Childhood	Netherlands
Saudino, Ronald & Plomin (2005)[273]	Twins Early Development Study (TEDS)	Childhood	United Kingdom
Silberg et. al. (1996)[274]	Virginia twin study of adolescent behavioral development (VTSABD)	Middle Childhood & Adolescence	United States
Smith et. al. (2011)[172]	Center for Antisocial Drug Dependence (CADD)	Adolescence	United States
**Heritability and environmental influences on specific learning disorders**			
Alarcon, DeFries & Fulker (1995)[276]	Colorado Learning Disabilities Research Center	Middle Childhood & Adolescence	United States

Bates et. al. (2004)[277]	Study of melanocytic naevi (moles)	Adolescence	Australia
Eaves et. al. (1997)[260]	Virginia twin study of adolescent behavioral development (VTSABD)	Middle Childhood & Adolescence	United States
Harlaar, Spinath, Dale & Plomin (2005)[278]	Twins Early Development Study (TEDS)	Childhood	United Kingdom
Reynolds et. al. (1996)[279]	Virginia twin study of adolescent behavioral development (VTSABD)	Middle Childhood	United States
Tosto et. al. (2014)[195]	Twins Early Development Study (TEDS)	Adolescence	United Kingdom
Grasby & Coventry (2016)[215]	Australian Twin Register	Middle Childhood	Australia
Shakeshaft et. al. (2013)[234]	Twins Early Development Study (TEDS)	Adolescence	United Kingdom
Tosto et. al. (2019)[238]	Twins Early Development Study (TEDS)	Adolescence	United Kingdom
**Heritability and environmental influences on motor disorders**			
van Beijsterveldt, Verhulst, Molenaar & Boomsma (2004)[266]	Netherlands twin register (NTR)	Childhood	Netherlands
Ooki (2005)[247]	Twin study in Japan	Childhood & Adolescence	Japan
**Genetic and environmental overlap between ASD & ADHD***			
Ronald, Simonoff, Kuntsi, Asherson & Plomin (2008)[51]	Twins Early Development Study (TEDS)	Middle childhood	United Kingdom
**Genetic and environmental overlap between ADHD & conduct disorder**			
Silberg et. al. (1996)[274]	Virginia twin study of adolescent behavioral development (VTSABD)	Middle childhood & Adolescence	United States
Knopik, Heath, Bucholz, Madden & Waldron (2009)[280]	Missouri Adolescent Female Twin Study cohort	Adolescence	United States

Supplementary Table 34. Overview of SNP-based studies using samples of males and females combined. Disorders annotated with an asterisk (*) indicate disorders for which meta-analysis could not be performed.

Reference	Cohort	Age category	Country
**Heritability and environmental influences on communication disorders**			
Cheesman et. al. (2017)[13]	Twins Early Development Study (TEDS)	Adolescence	United Kingdom
Trzaskowski, Dale & Plomin (2013)[19]	Twins Early Development Study (TEDS)	Adolescence	United Kingdom
Trzaskowski et. al. (2013)[33]	Twins Early Development Study (TEDS)	Adolescence	United Kingdom
Verhoef, Shapland, Fisher, Dale & St Pourcain (2020)[282]	Avon Longitudinal Study of Parents and Children (ALSPAC)	Middle Childhood	United Kingdom
**Heritability and environmental influences on ASD**			
Cheesman et. al. (2017)[13]	Twins Early Development Study (TEDS)	Adolescence	United Kingdom
Gandal et. al. (2018)[283]	Psychiatric Genomics Consortium (PGC), iPSYCH	Childhood & Adolescence	United Kingdom, Denmark
Grove et. al. (2019)[284]	Psychiatric Genomics Consortium (PGC), iPSYCH	Childhood & Adolescence	United Kingdom, Denmark
Hill et. al. (2016)[285]	Psychiatric Genomics Consortium (PGC)	Childhood & Adolescence	United Kingdom
Lee et. al. (2013)[286]	Psychiatric Genomics Consortium (PGC)	Childhood & Adolescence	United Kingdom
Serdarevic et. al. (2020)[287]	Generation R	Childhood	Netherlands
Solberg et. al. (2019)[288]	Psychiatric Genomics Consortium (PGC), iPSYCH	Childhood & Adolescence	United Kingdom, Denmark
St Pourcain et. al. (2014)[289]	Avon Longitudinal Study of Parents and Children (ALSPAC)	Middle Childhood	United Kingdom
St Pourcain et. al. (2018)[290]	Avon Longitudinal Study of Parents and Children (ALSPAC)	Middle Childhood	United Kingdom
St Pourcain et. al. (2018) [291]	Avon Longitudinal Study of Parents and Children (ALSPAC)	Middle Childhood	United Kingdom

Stergiakouli et. al. (2017)[292]	Avon Longitudinal Study of Parents and Children (ALSPAC)	Middle Childhood	United Kingdom
Trzaskowski, Dale & Plomin (2013)[19]	Avon Longitudinal Study of Parents and Children (ALSPAC)	Adolescence	United Kingdom
Warrier & Baron-Cohen (2018)[293]	Avon Longitudinal Study of Parents and Children (ALSPAC)	Adolescence	United Kingdom
The Autism Spectrum Disorders Working Group of The Psychiatric Genomics Consortium (2017)[294]	Psychiatric Genomics Consortium (PGC)	Childhood & Adolescence	United Kingdom
Pettersson et. al. (2019)[295]	Psychiatric Genomics Consortium (PGC), iPSYCH	Childhood & Adolescence	United Kingdom, Denmark
**Heritability and environmental influences on ADHD**			
Artigas et. al. (2020)[296]	Psychiatric Genomics Consortium (PGC), iPSYCH	Childhood & Adolescence	United Kingdom, Denmark
Cheesman et. al. (2017)[13]	Twins Early Development Study (TEDS)	Adolescence	United Kingdom
Demontis et. al. (2019)[297]	Psychiatric Genomics Consortium (PGC), iPSYCH	Childhood & Adolescence	United Kingdom, Denmark
Hill et. al. (2016)[285]	Psychiatric Genomics Consortium (PGC)	Childhood & Adolescence	United Kingdom
Lee et. al. (2013)[286]	Psychiatric Genomics Consortium (PGC)	Childhood & Adolescence	United Kingdom
Martin et. al. (2018)[298]	Psychiatric Genomics Consortium (PGC), iPSYCH	Childhood & Adolescence	United Kingdom, Denmark
Micalizzi et. al. (2021)[299]	Philadelphia Neurodevelopmental Cohort	Middle Childhood & Adolescence	United States
Middeldorp et. al. (2016)[300]	Avon Longitudinal Study of Parents and Children (ALSPAC)	Childhood	United Kingdom
Pappa et. al. (2015)[301]	Generation R, Netherlands twin register (NTR)	Childhood & Middle Childhood	Netherlands
Rovira et. al. (2020)[302]	Psychiatric Genomics Consortium (PGC), iPSYCH, IMpACT	Middle Childhood	United Kingdom, Denmark, United States
Solberg et. al. (2019)[288]	Psychiatric Genomics Consortium (PGC), iPSYCH	Childhood & Adolescence	United Kingdom, Denmark

Stergiakouli et. al. (2017)[292]	Avon Longitudinal Study of Parents and Children (ALSPAC)	Childhood	United Kingdom
Trzaskowski, Dale & Plomin (2013)[19]	Twins Early Development Study (TEDS)	Adolescence	United Kingdom
Pettersson et. al. (2019)[295]	Psychiatric Genomics Consortium (PGC), iPSYCH	Childhood & Adolescence	United Kingdom, Denmark
**Heritability and environmental influences on specific learning disorders**			
Cheesman et. al. (2017)[13]	Twins Early Development Study (TEDS)	Adolescence	United Kingdom
Davis et. al. (2014)[179]	Twins Early Development Study (TEDS), Avon Longitudinal Study of Parents and Children (ALSPAC)	Adolescence	United Kingdom
Gialluisi et. al. (2020)[303]	Study-specific multi-site cohort	Childhood & Adolescence	Multiple sites
Harlaar, Trzaskowski, Dale & Plomin (2014)[186]	Twins Early Development Study (TEDS)	Childhood	United Kingdom
Trzaskowski, Dale & Plomin (2013)[19]	Twins Early Development Study (TEDS)	Adolescence	United Kingdom
Rimfeld et. al. (2018)[230]	Twins Early Development Study (TEDS)	Childhood	United Kingdom
Rimfeld, Kovas, Dale & Plomin (2015)[233]	Twins Early Development Study (TEDS)	Adolescence	United Kingdom
Trzaskowski et. al. (2013)[33]	Twins Early Development Study (TEDS)	Adolescence	United Kingdom
Verhoef, Shapland, Fisher, Dale & St Pourcain (2020)[282]	Avon Longitudinal Study of Parents and Children (ALSPAC)	Childhood	United Kingdom
**Genetic and environmental overlap between ASD & ADHD**			
Demontis et. al. (2019)[297]	Psychiatric Genomics Consortium (PGC), iPSYCH	Childhood & Adolescence	United Kingdom, Denmark
Grove et. al. (2019)[284]	Psychiatric Genomics Consortium (PGC), iPSYCH	Childhood & Adolescence	United Kingdom, Denmark
Solberg et. al. (2019)[288]	Psychiatric Genomics Consortium (PGC)	Childhood & Adolescence	United Kingdom
Stergiakouli et. al. (2017)[292]	Avon Longitudinal Study of Parents and Children (ALSPAC)	Childhood & Middle Childhood	United Kingdom
Lee et. al. (2013)[286]	Psychiatric Genomics Consortium (PGC)	Childhood & Adolescence	United Kingdom

Supplementary Table 35. Overview of SNP-based studies using male samples. Disorders annotated with an asterisk (*) indicate disorders for which meta-analysis could not be performed.

Reference	Cohort	Age category	Country
**Heritability and environmental influences on ASD***			
Martin et. al. (2021)[304]	Psychiatric Genomics Consortium (PGC), iPSYCH	Childhood & Adolescence	United Kingdom, Denmark
**Heritability and environmental influences on ADHD**			
Martin et. al. (2018)[298]	Psychiatric Genomics Consortium (PGC), iPSYCH	Childhood & Adolescence	United Kingdom, Denmark
Martin et. al. (2021)[304]	Psychiatric Genomics Consortium (PGC), iPSYCH	Childhood & Adolescence	United Kingdom, Denmark

Supplementary Table 36. Overview of SNP-based studies using female samples. Disorders annotated with an asterisk (*) indicate disorders for which meta-analysis could not be performed.

Reference	Cohort	Age category	Country
**Heritability and environmental influences on ASD***			
Martin et. al. (2021)[304]	Psychiatric Genomics Consortium (PGC), iPSYCH	Childhood & Adolescence	United Kingdom, Denmark
**Heritability and environmental influences on ADHD**			
Martin et. al. (2018)[298]	Psychiatric Genomics Consortium (PGC), iPSYCH	Childhood & Adolescence	United Kingdom, Denmark
Martin et. al. (2021)[304]	Psychiatric Genomics Consortium (PGC), iPSYCH	Childhood & Adolescence	United Kingdom, Denmark

Supplementary Table 37. Heritability, shared and nonshared environmental influences on NDDs, stratified by designs.

Family-based designs							SNP-based designs		
**NDDs**	**Family h^2 (SE)**	**N**	**Family c^2 (SE)**	**N**	**Family e^2 (SE)**	**N**		**SNP h^2 (SE)**	**N**
**NDDs combined**									
Categorical threshold sibling study	0.67 (0.24)	3	-	-	0.2 (0.11)	2	GCTA (REML)	0.21 (0.05)	19
Categorical threshold twin and sibling study	0.85 (0.19)	2	-	-	0.37 (0.21)	3	LDSC	0.17 (0.04)	13
DF extremes twin and sibling study	0.83 (0.38)	4	0.17 (0.13)	4	-	-			
Classical twin and sibling study	0.57 (0.09)	8	0.08 (0.09)	2	0.45 (0.08)	8			
Categorical threshold twin study	0.74 (0.07)	23	0.25 (0.08)	11	0.27 (0.07)	21			
DF extremes twin study	0.7 (0.11)	57	0.19 (0.05)	22	0.27 (0.05)	20			
Classical twin study	0.65 (0.03)	157	0.15 (0.02)	95	0.27 (0.01)	151			
**Communication disorders**									
Categorical threshold twin study	0.47 (0.1)	5	0.47 (0.12)	4	0.13 (0.06)	5	GCTA (REML)	0.32 (0.14)	4
DF extremes twin study	0.78 (0.41)	8	0.31 (0.12)	5	0.22 (0.09)	5	LDSC	-	-
Classical twin study	0.56 (0.09)	11	0.29 (0.07)	7	0.25 (0.06)	8			
**ASD**									
Categorical threshold twin study	0.87 (0.11)	7	0.09 (0.15)	3	0.16 (0.07)	6	GCTA (REML)	0.17 (0.07)	9
DF extremes twin study	0.78 (0.36)	11	-	-	0.33 (0.07)	5	LDSC	0.13 (0.05)	8
Classical twin study	0.68 (0.04)	20	0.16 (0.07)	8	0.26 (0.03)	19			
**ADHD**									
Categorical threshold twin and sibling study	0.84 (0.21)	2	-	-	0.13 (0.09)	2	GCTA (REML)	0.17 (0.06)	8
DF extremes twin and sibling study	0.94 (0.46)	2	0.06 (0.25)	2	-	-	LDSC	0.22 (0.05)	7
Classical twin and sibling study	0.56 (0.1)	7	0.08 (0.09)	2	0.45 (0.08)	7			
Categorical threshold twin study	0.76 (0.1)	13	0.14 (0.09)	5	0.28 (0.08)	12			
DF extremes twin study	0.75 (0.18)	11	0.04 (0.08)	3	0.36 (0.14)	2			

Classical twin study	0.67 (0.03)	91	0.1 (0.03)	38	0.29 (0.02)	87			
**Specific learning disorders**									
DF extremes twin and sibling study	0.5 (0.13)	2	0.2 (0.15)	2	-	-	GCTA (REML)	0.31 (0.08)	8
DF extremes twin study	0.62 (0.06)	30	0.21 (0.06)	14	0.25 (0.06)	9	LDSC	-	-
Classical twin study	0.62 (0.05)	63	0.18 (0.02)	55	0.25 (0.02)	60			
**Motor disorders**									
Categorical threshold twin and sibling study	-	-	-	-	0.64 (0.18)	2			
Categorical threshold twin study	0.71 (0.1)	3	0.12 (0.12)	2	0.25 (0.12)	3			
Classical twin study	0.71 (0.23)	2	-	-	-	-			

Note. H^2= heritability; c^2= shared environmental influences; e^2= nonshared environmental influences; N= number of studies identified; SE= standard error; GCTA= genome-wide complex trait analysis; REML= restricted maximum likelihood; LDSC= linkage disequilibrium score regression.

Supplementary Table 38. Genetic, shared and nonshared environmental correlations between NDDs, stratified by designs.

**Family-based designs**							**SNP-based designs**		
**NDDs**	**Family rA (SE)**	**N**	**Family rC (SE)**	**N**	**Family rE (SE)**	**N**		**SNP rG (SE)**	**N**
**NDDs combined**									
Categorical threshold twin study	0.67 (0.48)	2	-	-	-	-	GCTA (REML)	0.5 (0.36)	3
DF extremes twin study	0.40 (0.09)	15	-	-	0.13 (0.12)	2	LDSC	0.26 (0.14)	3
Classical twin study	0.50 (0.08)	21	0.69 (0.37)	15	0.18 (0.05)	20			
**ASD & ADHD**									
Classical twin study	0.56 (0.34)	5	-	-	0.22 (0.13)	5	GCTA (REML)	0.36 (0.49)	2
							LDSC	0.26 (0.14)	3
**ADHD & motor disorders**									
Categorical threshold twin study	0.9 (0.82)	2	-	-	-	-		-	-
**ADHD & specific learning disorders**									
DF extremes twin study	0.47 (0.07)	9	-	-	-	-		-	-
Classical twin study	0.29 (0.05)	9	0.32 (0.14)	7	0.10 (0.05)	8		-	-

Note. rA/rG= genetic correlation; rC= shared environmental correlation; rE= nonshared environmental correlation; N= number of studies identified; SE= standard error; GCTA= genome-wide complex trait analysis; REML= restricted maximum likelihood; LDSC= linkage disequilibrium score regression.

Supplementary Table 39. Genetic, shared and nonshared environmental correlations between NDDs and DICCs, stratified by designs.

NDDs and DICCs	Family rA (SE)	N	Family rC (SE)	N	Family rE (SE)	N
**NDDs and DICCs combined**						
Classical twin study	0.62 (0.19)	15	0.88 (0.34)	11	0.38 (0.14)	13
**ADHD & conduct disorder**						
Classical twin study	0.66 (0.36)	6	0.94 (0.71)	3	0.11 (0.08)	5
**ADHD & oppositional defiant disorder**						
Classical twin study	0.66 (0.18)	6	0.96 (0.57)	4	0.54 (0.25)	5
**ASD & conduct disorder**						
Classical twin study	0.35 (0.10)	3	0.88 (0.57)	3	0.07 (0.08)	3
Note. rA/rG= genetic correlation; rC= shared environmental correlation; rE= nonshared environmental correlation; N= number of studies identified; SE= standard error; GCTA= genome-wide complex trait analysis; REML= restricted maximum likelihood; LDSC= linkage disequilibrium score regression.						

Supplementary Table 40. Heritability, shared and nonshared environmental influences on NDDs, stratified by models.

NDDs	Family h^2 (SE)	N	Family c^2 (SE)	N	Family e^2 (SE)	N
**NDDs combined**						
A only	0.74 (0.16)	11	-	-	-	-
Best fitting	0.7 (0.05)	82	-	-	0.34 (0.02)	81
Full ACE	0.61 (0.03)	104	0.16 (0.02)	104	0.22 (0.01)	104
DF extremes A only	0.77 (0.16)	31	-	-	-	-
DF extremes best fitting	0.72 (0.16)	18	0.24 (0.07)	12	0.33 (0.06)	7
DF extremes full ACE	0.6 (0.07)	15	0.17 (0.05)	15	0.24 (0.05)	15
Twin correlations	0.67 (0.07)	14	0.17 (0.07)	4	0.37 (0.06)	13
**Communication disorders**						
A only	0.55 (0.2)	3	-	-	-	-
Best fitting	0.57 (0.22)	4	-	-	0.51 (0.19)	4
Full ACE	0.47 (0.06)	11	0.37 (0.07)	11	0.19 (0.04)	11
DF extremes A only	0.94 (0.56)	4	-	-	-	-
DF extremes best fitting	0.55 (0.2)	3	0.45 (0.26)	2	-	-
DF extremes full ACE	0.47 (0.13)	5	0.3 (0.11)	5	0.23 (0.09)	5
**ASD**						
A only	0.83 (0.38)	4	-	-	-	-
Best fitting	0.71 (0.09)	13	-	-	0.28 (0.04)	13
Full ACE	0.72 (0.1)	11	0.11 (0.06)	11	0.21 (0.05)	11
DF extremes A only	0.86 (0.45)	3	-	-	-	-
DF extremes best fitting	0.67 (0.07)	6	-	-	0.33 (0.07)	5
Twin correlations	0.7 (0.07)	4	0.16 (0.08)	2	0.25 (0.11)	4
**ADHD**						

A only	0.7 (0.21)	4	-	-	-	-
Best fitting	0.7 (0.05)	61	-	-	0.33 (0.02)	59
Full ACE	0.65 (0.04)	43	0.1 (0.02)	43	0.24 (0.02)	43
DF extremes A only	0.79 (0.28)	9	-	-	-	-
DF extremes best fitting	0.88 (0.24)	4	0.08 (0.2)	3	-	-
Twin correlations	0.67 (0.1)	11	0.2 (0.13)	2	0.38 (0.07)	10
**Specific learning disorders**						
A only	0.58 (0.09)	4	-	-	-	-
Best fitting	0.73 (0.17)	9	-	-	0.34 (0.11)	9
Full ACE	0.6 (0.05)	54	0.18 (0.02)	54	0.24 (0.02)	54
DF extremes A only	0.64 (0.09)	16	-	-	-	-
DF extremes best fitting	0.55 (0.08)	7	0.22 (0.08)	7	-	-
DF extremes full ACE	0.63 (0.08)	9	0.18 (0.07)	9	0.24 (0.06)	9
**Motor disorders**						
Best fitting	0.77 (0.18)	3	-	-	0.39 (0.14)	4
Full ACE	0.69 (0.1)	3	0.13 (0.11)	3	0.24 (0.13)	3
Note. H^2= heritability; c^2= shared environmental influences; e^2= nonshared environmental influences; N= number of studies identified; SE= standard error.						

Supplementary Table 41. Genetic, shared and nonshared environmental correlations between NDDs, stratified by models.

NDDs	Family rA (SE)	N	Family rC (SE)	N	Family rE (SE)	N
**NDDs combined**						
A only	0.68 (0.48)	2	-	-	-	-
Best fitting	0.45 (0.19)	8	-	-	0.14 (0.05)	7
Full ACE	0.48 (0.08)	16	0.67 (0.39)	15	0.18 (0.06)	16
DF extremes A only	0.39 (0.1)	13	-	-	-	-
**ASD & ADHD**						
Best fitting	0.68 (0.49)	3	-	-	0.18 (0.09)	3
Full ACE	0.42 (0.17)	2	-	-	0.31 (0.21)	2
**ADHD & specific learning disorders**						
Best fitting	0.30 (0.08)	5	-	-	0.11 (0.08)	4
Full ACE	0.27 (0.06)	6	0.31 (0.15)	6	0.1 (0.05)	6
DF extremes A only	0.48 (0.08)	8	-	-	-	-
Note. rA/rG= genetic correlation; rC= shared environmental correlation; rE= nonshared environmental correlation; N= number of studies identified; SE= standard error.						

Supplementary Table 42. Genetic, shared and nonshared environmental correlations between NDDs and DICCs, stratified by models.

**NDDs and DICCs**	**Family rA (SE)**	**N**	**Family rC (SE)**	**N**	**Family rE (SE)**	**N**
**NDDs and DICCs combined**						
Best fitting	0.69 (0.3)	7	-	-	0.15 (0.07)	5
Full ACE	0.48 (0.14)	10	0.9 (0.35)	10	0.42 (0.18)	10
**ADHD & conduct disorder**						
Best fitting	0.78 (0.5)	4	-	-	0.14 (0.13)	3
Full ACE	0.33 (0.12)	3	0.94 (0.71)	3	0.07 (0.1)	3
**ADHD & oppositional defiant disorder**						
Best fitting	0.69 (0.24)	3	-	-	0.42 (0.13)	2
Full ACE	0.56 (0.24)	4	0.96 (0.57)	4	0.54 (0.3)	4
**ASD & conduct disorder**						
Full ACE	0.35 (0.11)	3	0.88 (0.57)	3	0.06 (0.08)	3
Note. rA/rG= genetic correlation; rC= shared environmental correlation; rE= nonshared environmental correlation; N= number of studies identified; SE= standard error.						

Supplementary Table 43. Heritability, shared and nonshared environmental influences on NDDs, stratified by raters.

NDDs	Family h^2 (SE)	N	Family c^2 (SE)	N	Family e^2 (SE)	N	SNP h^2 (SE)	N
**NDDs combined**								
Diagnosis	0.81 (0.15)	7	0.02 (0.09)	2	0.3 (0.11)	6	0.17 (0.04)	11
Parent	0.7 (0.04)	110	0.15 (0.03)	48	0.25 (0.02)	93	0.19 (0.07)	10
Parent & Self	0.72 (0.1)	8	0.09 (0.15)	2	0.31 (0.06)	8	-	-
Parent & Teacher	0.72 (0.06)	17	0.04 (0.08)	5	0.3 (0.04)	14	-	-
Researcher	0.71 (0.18)	2	0.02 (0.05)	2	0.18 (0.16)	2	-	-
Self-report	0.5 (0.07)	19	0.12 (0.11)	5	0.55 (0.05)	17	0.05 (0.18)	2
Teacher	0.65 (0.03)	29	0.18 (0.07)	12	0.34 (0.05)	28	0.3 (0.19)	5
Cognitive test	0.6 (0.04)	98	0.21 (0.02)	71	0.25 (0.02)	73	0.29 (0.07)	10
**Intellectual disabilities**								
Diagnosis	0.86 (0.44)	2	-	-	0.1 (0.16)	2	-	-
**Communication disorders**								
Parent	0.76 (0.22)	7	0.43 (0.14)	4	0.14 (0.06)	6	-	-
Teacher	0.62 (0.11)	2	-	-	0.17 (0.08)	2	-	-
Cognitive test	0.6 (0.21)	18	0.31 (0.06)	12	0.25 (0.05)	13	0.32 (0.14)	4
**ASD**								
Diagnosis	0.85 (0.15)	4	0.01 (0.1)	2	0.19 (0.11)	3	0.12 (0.05)	6
Parent	0.78 (0.21)	27	0.19 (0.07)	11	0.24 (0.03)	20	0.2 (0.07)	8
Parent & Teacher	0.63 (0.11)	3	-	-	0.41 (0.12)	3	-	-
Self-report	0.52 (0.12)	2	-	-	-	-	-	-
Teacher	0.58 (0.07)	6	0.04 (0.1)	2	0.42 (0.07)	5	0 (0.21)	2
**ADHD**								
Diagnosis	0.79 (0.24)	4	-	-	0.29 (0.16)	4	0.21 (0.05)	7

Parent	0.7 (0.04)	83	0.09 (0.03)	34	0.23 (0.02)	72	0.13 (0.1)	5
Parent & Self	0.72 (0.1)	8	0.09 (0.15)	2	0.31 (0.06)	8	-	-
Parent & Teacher	0.71 (0.05)	15	0.04 (0.08)	5	0.29 (0.05)	12	-	-
Self-report	0.5 (0.08)	18	0.12 (0.11)	5	0.56 (0.05)	16	0.02 (0.18)	2
Teacher	0.65 (0.05)	18	0.16 (0.11)	5	0.37 (0.04)	17	0.38 (0.23)	3
**Specific learning disorders**								
Parent	0.72 (0.25)	2	-	-	0.23 (0.08)	2	-	-
Teacher	0.67 (0.05)	5	0.16 (0.06)	4	0.22 (0.04)	5	-	-
Cognitive test	0.6 (0.04)	85	0.19 (0.02)	62	0.24 (0.02)	63	0.32 (0.09)	8
**Motor disorders**								
Diagnosis	0.73 (0.15)	3	-	-	0.32 (0.16)	3	-	-
Parent	0.71 (0.11)	4	0.12 (0.12)	2	0.39 (0.12)	4	-	-

Note. H^2= heritability; c^2= shared environmental influences; e^2= nonshared environmental influences; N= number of studies identified; SE= standard error.

Supplementary Table 44. Genetic, shared and nonshared environmental correlations between NDDs, stratified by raters.

NDDs	Family rA (SE)	N	Family rC (SE)	N	Family rE (SE)	N	SNP rG (SE)	N
**NDDs combined**								
Parent	0.45 (0.12)	15	0.64 (0.45)	5	0.17 (0.07)	9	-	-
Parent & Teacher	0.45 (0.07)	8	-	-	0.18 (0.1)	3	-	-
Teacher	0.62 (0.27)	3	0.88 (0.57)	3	0.18 (0.1)	3	-	-
Cognitive test	0.5 (0.09)	11	0.69 (0.42)	7	0.17 (0.07)	7	0.25 (0.14)	5
**ASD & ADHD**								
Parent	0.67 (0.3)	5	-	-	0.22 (0.12)	4	-	-
**ADHD & motor disorders**								
Parent	0.9 (0.82)	2	-	-	-	-	-	-
**ADHD & specific learning disorders**								
Parent	0.28 (0.07)	8	0.25 (0.12)	3	0.11 (0.06)	4	-	-
Parent & Teacher	0.47 (0.08)	7	-	-	0.26 (0.15)	2	-	-
Teacher	0.4 (0.23)	2	0.69 (0.2)	2	0.1 (0.08)	2	-	-
**Communication disorders & specific learning disorders**								
Cognitive test	0.66 (0.15)	2	-	-	-	-	-	-
Note. rA/rG= genetic correlation; rC= shared environmental correlation; rE= nonshared environmental correlation; N= number of studies identified; SE= standard error.								

Supplementary Table 45. Genetic, shared and nonshared environmental correlations between NDDs and DICCs, stratified by raters.

NDDs and DICCs	Family rA (SE)	N	Family rC (SE)	N	Family rE (SE)	N
**NDDs and DICCs combined**						
Parent	0.72 (0.34)	6	0.93 (0.57)	4	0.2 (0.09)	5
Parent & Self	0.63 (0.5)	2	0.97 (0.53)	2	0.7 (0.61)	2
Parent & Teacher	0.6 (0.28)	3	0.82 (0.68)	3	0.66 (0.6)	2
Self-report	0.51 (0.25)	2	-	-	0.11 (0.14)	2
**ADHD & conduct disorder**						
Parent	0.85 (0.61)	3	-	-	0.22 (0.15)	2
**ADHD & oppositional defiant disorder**						
Parent	0.73 (0.32)	2	-	-	-	-
Note. rA/rG= genetic correlation; rC= shared environmental correlation; rE= nonshared environmental correlation; N= number of studies identified; SE= standard error.						

Supplementary Table 46. Heritability, shared and nonshared environmental influences on NDDs, stratified by number of covariates included in analyses.

NDDs	Family h^2 (SE)	N	Family c^2 (SE)	N	Family e^2 (SE)	N	SNP h^2 (SE)	N
**NDDs combined**								
0	0.67 (0.04)	56	0.22 (0.05)	25	0.31 (0.03)	39	-	-
1	0.68 (0.06)	56	0.16 (0.04)	25	0.27 (0.03)	40	0.16 (0.07)	2
2	0.64 (0.03)	113	0.15 (0.02)	69	0.3 (0.03)	104	0.17 (0.16)	3
3	0.61 (0.11)	9	0.18 (0.07)	5	0.31 (0.08)	9	0.26 (0.06)	14
4	0.73 (0.18)	5	0.17 (0.08)	3	0.23 (0.07)	4	-	-
**Intellectual disabilities**								
1	0.86 (0.44)	2	-	-	0.1 (0.16)	2	-	-
**Communication disorders**								
0	0.47 (0.1)	5	0.52 (0.11)	3	0.15 (0.07)	4	-	-
1	0.77 (0.24)	7	0.29 (0.15)	3	0.21 (0.1)	5	-	-
2	0.5 (0.06)	10	0.28 (0.07)	8	0.26 (0.09)	8	-	-
**ASD**								
0	0.8 (0.19)	11	0.03 (0.05)	4	0.3 (0.1)	6	-	-
1	0.76 (0.09)	3	-	-	0.25 (0.09)	3	-	-
2	0.68 (0.04)	20	0.17 (0.08)	8	0.26 (0.03)	17	-	-
**ADHD**								
0	0.68 (0.05)	31	0.17 (0.07)	12	0.36 (0.04)	26	-	-
1	0.71 (0.09)	25	0.08 (0.05)	10	0.29 (0.05)	21	0.17 (0.07)	2
2	0.65 (0.04)	58	0.09 (0.03)	24	0.33 (0.04)	54	-	-
3	0.66 (0.22)	4	-	-	0.34 (0.17)	4	0.15 (0.11)	5
4	0.83 (0.16)	3	-	-	0.11 (0.09)	2	-	-
**Specific learning disorders**								

0	0.58 (0.06)	13	0.22 (0.07)	8	0.21 (0.06)	6	-	-
1	0.66 (0.07)	26	0.21 (0.06)	14	0.18 (0.03)	15	-	-
2	0.59 (0.03)	46	0.18 (0.03)	39	0.26 (0.02)	41	-	-
3	0.56 (0.06)	6	0.17 (0.08)	4	0.32 (0.06)	6	0.31 (0.09)	7
**Motor disorders**								
1	0.7 (0.09)	4	0.21 (0.15)	2	0.43 (0.17)	4	-	-
2	0.8 (0.05)	2	-	-	-	-	-	-
Note. H^2= heritability; c^2= shared environmental influences; e^2= nonshared environmental influences; N= number of studies identified; SE= standard error.								

Supplementary Table 47. Genetic, shared and nonshared environmental correlations between NDDs, stratified by number of covariates included in analyses.

NDDs	Family rA (SE)	N	Family rC (SE)	N	Family rE (SE)	N
**NDDs combined**						
0	0.37 (0.08)	8	-	-	-	-
1	0.52 (0.21)	7	0.1 (0.09)	2	0.02 (0.08)	2
2	0.55 (0.13)	20	0.8 (0.35)	13	0.17 (0.03)	17
3	0.53 (0.12)	2	-	-	0.44 (0.14)	2
**ASD & ADHD**						
2	0.68 (0.49)	4	-	-	0.18 (0.09)	4
**ADHD & motor disorders**						
1	0.9 (0.82)	2	-	-	-	-
**ADHD & specific learning disorders**						
0	0.41 (0.12)	4	-	-	-	-
1	0.28 (0.1)	4	-	-	-	-
2	0.32 (0.05)	9	0.4 (0.14)	6	0.12 (0.05)	7
**Communication disorders & specific learning disorders**						
2	0.66 (0.15)	2	-	-	-	-
Note. rA/rG= genetic correlation; rC= shared environmental correlation; rE= nonshared environmental correlation; N= number of studies identified; SE= standard error.						

Supplementary Table 48. Genetic, shared and nonshared environmental correlations between NDDs and DICCs, stratified by number of covariates included in analyses.

NDDs and DICCs	Family rA (SE)	N	Family rC (SE)	N	Family rE (SE)	N
**NDDs and DICCs combined**						
0	0.39 (0.13)	6	0.71 (0.6)	4	0.2 (0.09)	5
1	0.58 (0.19)	5	0.94 (0.55)	4	0.44 (0.34)	4
2	0.93 (0.74)	3	0.93 (0.77)	2	0.58 (0.41)	3
**ADHD & conduct disorder**						
0	0.43 (0.24)	2	-	-	0.12 (0.16)	2
1	0.37 (0.1)	3	0.87 (0.86)	2	0.05 (0.1)	2
**ADHD & oppositional defiant disorder**						
0	0.62 (0.25)	2	-	-	0.35 (0.17)	2
1	0.56 (0.29)	3	0.87 (0.86)	2	0.32 (0.1)	2
Note. rA/rG= genetic correlation; rC= shared environmental correlation; rE= nonshared environmental correlation; N= number of studies identified; SE= standard error.						

Supplementary Table 49. Heritability, shared and nonshared environmental influences on NDDs, stratified by measurement instruments.

**Measures from family-based studies**							**Measures from SNP-based studies**		
**NDDs**	**Family h^2 (SE)**	**N**	**Family c^2 (SE)**	**N**	**Family e^2 (SE)**	**N**		**SNP h^2 (SE)**	**N**
**Intellectual disabilities**									
ICD-9/ICD-10	0.86 (0.44)	2	-	-	0.1 (0.16)	2			
**Communication disorders**									
Clinical evaluation	0.75 (0.13)	3	-	-	0.27 (0.15)	2	TOAL	0.32 (0.16)	3
Goldman-Fristoe Test of Articulation	0.58 (0.2)	2	0.27 (0.2)	2	0.16 (0.12)	2		-	-
MCDI	0.46 (0.13)	3	0.53 (0.11)	3	0.05 (0.05)	3		-	-
TEGI	0.74 (0.32)	2	0.12 (0.2)	2	0.19 (0.2)	2		-	-
**ASD**									
A-TAC	0.73 (0.06)	8	0.14 (0.08)	3	0.29 (0.04)	7	AQ	-	-
ADI-R & ADOS	0.81 (0.62)	2	0.28 (0.3)	2	-	-	CAST	0.03 (0.18)	2
AQ	0.51 (0.1)	3	-	-	0.2 (0.17)	2	ICD-9/ICD-10	0.12 (0.05)	7
ADI-R	0.81 (0.45)	3	0.3 (0.22)	2	0.14 (0.22)	2	SCDC	0.24 (0.1)	4
CAST	0.7 (0.04)	14	0.09 (0.06)	4	0.27 (0.03)	11		-	-
DAWBA	0.75 (0.15)	3	-	-	0.22 (0.17)	2		-	-
DSM-4/DSM-5	0.69 (0.08)	2	-	-	0.31 (0.09)	2		-	-
ICD-9/ICD-10	0.8 (0.12)	3	0.01 (0.1)	2	0.19 (0.11)	3		-	-
**ADHD**									
A-TAC	0.78 (0.1)	5	0.03 (0.07)	2	0.25 (0.05)	5	CBRS	0.13 (0.13)	3
ATBRS	0.82 (0.07)	3	0.23 (0.14)	3	0.12 (0.08)	3	ICD-9/ICD-10	0.21 (0.21)	7
CBCL	0.61 (0.09)	14	0.05 (0.06)	5	0.25 (0.04)	11	SDQ	0.09 (0.09)	4
CBCL & YSR	0.78 (0.06)	3	-	-	0.25 (0.09)	3	TRF	0.53 (0.53)	2
CBRS	0.72 (0.03)	29	0.18 (0.16)	11	0.24 (0.03)	28		-	-

DBD	0.69 (0.25)	3	0.16 (0.13)	3	0.19 (0.07)	3		-	-
DBRS	0.76 (0.11)	4	0.03 (0.1)	3	0.25 (0.08)	3		-	-
DCB	0.67 (0.07)	2	-	-	-	-		-	-
DICA	0.69 (0.21)	3	-	-	-	-		-	-
DISC	0.51 (0.1)	5	0.03 (0.16)	2	0.54 (0.11)	4		-	-
DSM-4/DSM-5	0.77 (0.29)	9	0.11 (0.11)	4	0.35 (0.08)	7		-	-
DuPaul ADHD Rating Scale	0.75 (0.05)	4	0.29 (0.12)	2	0.25 (0.07)	4		-	-
ECRS	0.77 (0.23)	2	-	-	0.28 (0.1)	2		-	-
ICD-9/ICD-10	0.87 (0.11)	3	-	-	0.12 (0.05)	3		-	-
Rutter Scales	0.75 (0.15)	4	-	-	0.26 (0.13)	2		-	-
SBQ	0.61 (0.26)	2	-	-	0.38 (0.19)	2		-	-
SDQ	0.65 (0.1)	15	0.07 (0.12)	4	0.43 (0.07)	14		-	-
SWAN	0.73 (0.16)	8	0.35 (0.09)	5	0.14 (0.05)	7		-	-
TRF	0.6 (0.12)	4	-	-	0.46 (0.08)	3		-	-
**Specific learning disorders**									
Comprehensive Test of Phonological Processing	0.55 (0.17)	3	0.22 (0.16)	3	0.27 (0.1)	3	GCSE	0.34 (0.2)	2
FCAT	0.46 (0.13)	4	0.31 (0.14)	4	0.23 (0.07)	4	NFER	0.31 (0.16)	3
GCSE	0.61 (0.07)	5	0.22 (0.07)	5	0.18 (0.04)	5	PIAT	0.24 (0.22)	2
National Curriculum	0.64 (0.08)	7	0.15 (0.05)	7	0.23 (0.03)	7	TOWRE	0.36 (0.2)	2
NFER	0.49 (0.06)	9	0.17 (0.07)	7	0.33 (0.05)	7	National Curriculum	0.33 (0.18)	2
PIAT	0.56 (0.07)	21	0.22 (0.06)	14	0.25 (0.07)	13		-	-
PIAT & GOAL	0.59 (0.09)	5	0.21 (0.07)	4	0.35 (0.1)	4		-	-
PIAT & TOWRE	0.66 (0.15)	2	-	-	-	-		-	-
PIAT & WISC	0.59 (0.15)	5	0.23 (0.19)	3	0.11 (0.18)	2		-	-
PIAT & WRAT	0.51 (0.2)	3	-	-	-	-		-	-
TOWRE	0.7 (0.07)	8	0.13 (0.06)	8	0.17 (0.04)	8		-	-

WISC	0.41 (0.27)	2	-	-	-	-		-	-
The Woodcock–Johnson Tests of Cognitive Abilities	0.57 (0.11)	8	0.19 (0.1)	7	0.24 (0.06)	7		-	-
TOWRE & The Woodcock–Johnson Tests of Cognitive Abilities	0.77 (0.16)	2	-	-	-	-		-	-
WRAT	0.48 (0.19)	2	0.33 (0.18)	2	0.2 (0.12)	2		-	-
**Motor disorders**									
A-TAC	0.58 (0.12)	2	-	-	0.42 (0.12)	2	-	-	-

Note. H^2= heritability; c^2= shared environmental influences; e^2= nonshared environmental influences; N= number of studies identified; SE= standard error; TOAL= Test of Adolescent and Adult Language; MCDI= MacArthur-Bates Communicative Development Inventories; TEGI= Test of Early Grammatical Impairment; A-TAC= Autism-Tics, AD/HD, and other Comorbidities Inventory; ADI-R= The Autism Diagnostic Interview-Revised; ADOS= Autism Diagnostic Observation Schedule; AQ= Autism Spectrum Quotient; CAST= Childhood Autism Spectrum Test; SCDC= Social and Communication Disorders Checklist; DAWBA= Developmental and Well-Being Assessment; DSM= Diagnostic Statistical Manual; ICD= International Classification of Diseases; ATBRS= Australian Twin Behaviour Rating Scale; CBRS= Conners Comprehensive Behaviour Rating Scale; CBCL= Child Behavior Checklist; YSR= Youth Self-Report; DBD= Disruptive Behavior Disorder Rating Scale; DBRS= The Disruptive Behavior Rating Scale; DCB= Devereux Child Behavior Rating Scale; DICA= Diagnostic Interview for Children and Adolescents; DISC= Diagnostic Interview Schedule for Children; ECRS= Emory Combined Rating Scale; SBQ= Social Behavior Questionnaire; SDQ= Strengths and Difficulties Questionnaire; SWAN= Strengths and Weaknesses of Attention-Deficit/Hyperactivity-symptoms and Normal-behaviors; TRF= Teacher Report Form; FCAT= The Florida Comprehensive Assessment Test; GCSE= General Certificate of Secondary Education; NFER= National Foundation for Educational Research; PIAT= The Peabody Individual Achievement Test; GOAL= Greater Opportunities for Adult Learning Success; TOWRE= Test of Word Reading Efficiency; WISC= Wechsler Intelligence Scale for Children; WRAT= Wide Range Achievement Test.

Supplementary Table 50. Genetic, shared and nonshared environmental correlations between NDDs, stratified by measurement instruments.

NDDs	Family rA (SE)	N	Family rC (SE)	N	Family rE (SE)	N
**ASD & ADHD**						
A-TAC	0.8 (0.25)	3	-	-	0.36 (0.12)	2
CAST & CBRS	0.26 (0.1)	2	-	-	0.1 (0.08)	2
**ADHD & specific learning disorders**						
CBRS & PIAT	0.29 (0.1)	2	0.23 (0.13)	2	0.1 (0.08)	2
CBRS & RDQ	0.48 (0.13)	2	-	-	0.26 (0.15)	2
DBRS & PIAT	0.50 (0.12)	3	-	-	-	-
DICA & PIAT	0.35 (0.18)	2	-	-	-	-

Note. rA/rG= genetic correlation; rC= shared environmental correlation; rE= nonshared environmental correlation; N= number of studies identified; SE= standard error; A-TAC= Autism-Tics, AD/HD, and other Comorbidities Inventory; CAST= Childhood Autism Spectrum Test; CBRS= Conners Comprehensive Behaviour Rating Scale; PIAT= The Peabody Individual Achievement Test; DBRS= The Disruptive Behavior Rating Scale; DICA= Diagnostic Interview for Children and Adolescents; RDQ= Reading Difficulties Questionnaire.

Supplementary Figures

Supplementary Figure 1. Distribution of estimates. 121
Supplementary Figure 2. Heritability (h2), shared (c2) and nonshared (e2) environmental influences on specific phenotypes within neurodevelopmental disorders (NDDs) categories. 122
Supplementary Figure 3. Genetic (rA), shared (rC) and nonshared (rE) environmental overlap between specific phenotypes within the neurodevelopmental disorders (NDDs) category and between specific phenotypes within the NDDs and disruptive, impulse control and conduct disorders (DICCs) category.....123
Supplementary Figure 4. Variance in heritability (h2), shared (c2) and nonshared (e2) environmental influences on neurodevelopmental disorders (NDDs) (top panel), variance in genetic (rA/rG), shared (rC) and nonshared (rE) environmental correlations between NDDs (middle panel) and variance in genetic and environmental correlations between NDDs and disruptive, impulse control and conduct disorders (DICCs) that can be attributed to heterogeneity (the I2 statistic). 124
Supplementary Figure 5. Results of the influential cases identification analysis. The baujat plots present studies determined to have a significant impact on the grand estimates of heritability (h2), shared (c2) and nonshared (e2) environmental influences on neurodevelopmental disorders (NDDs) and/or heterogeneity of estimates. 125
Supplementary Figure 6. Results of the influential cases identification analysis. The baujat plots present studies determined to have a significant impact on the grand estimates of genetic (rA), shared (rC) and nonshared (rE) environmental overlap between neurodevelopmental disorders (NDDs) and/or heterogeneity of estimates. 126
Supplementary Figure 7. Results of the influential cases identification analysis. The baujat plots present studies determined to have a significant impact on the grand estimates of genetic (rA), shared (rC) and nonshared (rE) environmental overlap between neurodevelopmental disorders (NDDs) and disruptive, impulse control and conduct disorders (DICCs) and/or heterogeneity of estimates. 127
Supplementary Figure 8. Funnel plots involving all studies addressing heritability (h2), shared (c2) and nonshared (e2) environmental influences on neurodevelopmental disorders (NDDs). 128
Supplementary Figure 9. Funnel plots involving all studies addressing heritability (h2) and nonshared (e2) environmental influences on intellectual disabilities. 129
Supplementary Figure 10. Funnel plots involving all studies addressing heritability (h2), shared (c2) and nonshared (e2) environmental influences on communication disorders....130
Supplementary Figure 11. Funnel plots involving all studies addressing heritability (h2), shared (c2) and nonshared (e2) environmental influences on ASD. 131
Supplementary Figure 12. Funnel plots involving all studies addressing heritability (h2), shared (c2) and nonshared (e2) environmental influences on ADHD....132
Supplementary Figure 13. Funnel plots involving all studies addressing heritability (h2), shared (c2) and nonshared (e2) environmental influences on specific learning disorders. 133
Supplementary Figure 14. Funnel plots involving all studies addressing heritability (h2), shared (c2) and nonshared (e2) environmental influences on motor disorders. 134
Supplementary Figure 15. Funnel plots involving all studies addressing genetic (rA), shared (rC) and nonshared (rE) environmental overlap between neurodevelopmental disorders (NDDs). 135
Supplementary Figure 16. Funnel plots involving all studies addressing genetic (rA), and nonshared (rE) environmental overlap between ASD & ADHD. 136
Supplementary Figure 17. Funnel plots involving all studies addressing genetic overlap (rA) between ADHD & motor disorders....137
Supplementary Figure 18. Funnel plots involving all studies addressing genetic (rA), shared (rC) and nonshared (rE) environmental overlap between ADHD & specific learning disorders. 138
Supplementary Figure 19. Funnel plots involving all studies addressing genetic overlap (rA) between communication disorders & motor disorders....139
Supplementary Figure 20. Funnel plots involving all studies addressing genetic overlap (rA) between communication disorders & specific learning disorders....140

Supplementary Figure 21. Funnel plots involving all studies addressing genetic (rA), shared (rC) and nonshared (rE) environmental overlap between neurodevelopmental disorders (NDDs) and disruptive, impulse control and conduct disorders (DICCs)....141
Supplementary Figure 22. Funnel plots involving all studies addressing genetic (rA), shared (rC) and nonshared (rE) environmental overlap between ADHD & conduct disorder....142
Supplementary Figure 23. Funnel plots involving all studies addressing genetic (rA), shared (rC) and nonshared (rE) environmental overlap between ADHD & oppositional defiant disorder....143
Supplementary Figure 24. Funnel plots involving all studies addressing genetic (rA), shared (rC) and nonshared (rE) environmental overlap between ASD & conduct disorder. 144
Supplementary Figure 25. Results of the study quality assessment, illustrated as the percentage of studies showing low, moderate and high risk of bias. 145
Supplementary Figure 26. Heritability (h2), shared (c2) and nonshared (e2) environmental influences on neurodevelopmental disorders (NDDs) (top panel), genetic (rA/rG), shared (rC) and nonshared (rE) environmental overlap between NDDs (middle panel) and genetic and environmental overlap between NDDs and disruptive, impulse control and conduct disorders (DICCs) (bottom panel), stratified by measurement scales, i.e., categorical versus continuous measurement. 146
Supplementary Figure 27. Changes in family-based heritability (h2), shared (c2) and nonshared (e2) environmental influences on neurodevelopmental disorders (NDDs), as a function of sample ancestral composition....147
Supplementary Figure 28. Geographical differences in rA, rC and rE between NDDs and disruptive, impulse control and conduct disorders (DICCs)....148
Supplementary Figure 29. Diagram of searches and screening....149
Supplementary Figure 30. Grand heritability (h2), shared (c2) and nonshared (e2) environmental influences across all neurodevelopmental disorders (NDDs) (panel **A**), grand genetic (rA), shared (rC) and nonshared (rE) environmental correlations across all NDDs (panel **B**) and grand genetic and environmental correlations across NDDs and disruptive, impulse control and conduct disorders (DICCs) (panel **C**) obtained using different aggregation techniques, i.e., aggregating by study, cohort, and country, using correlation thresholds of r= 0.3, r= 0.5 and r= 0.9. 150

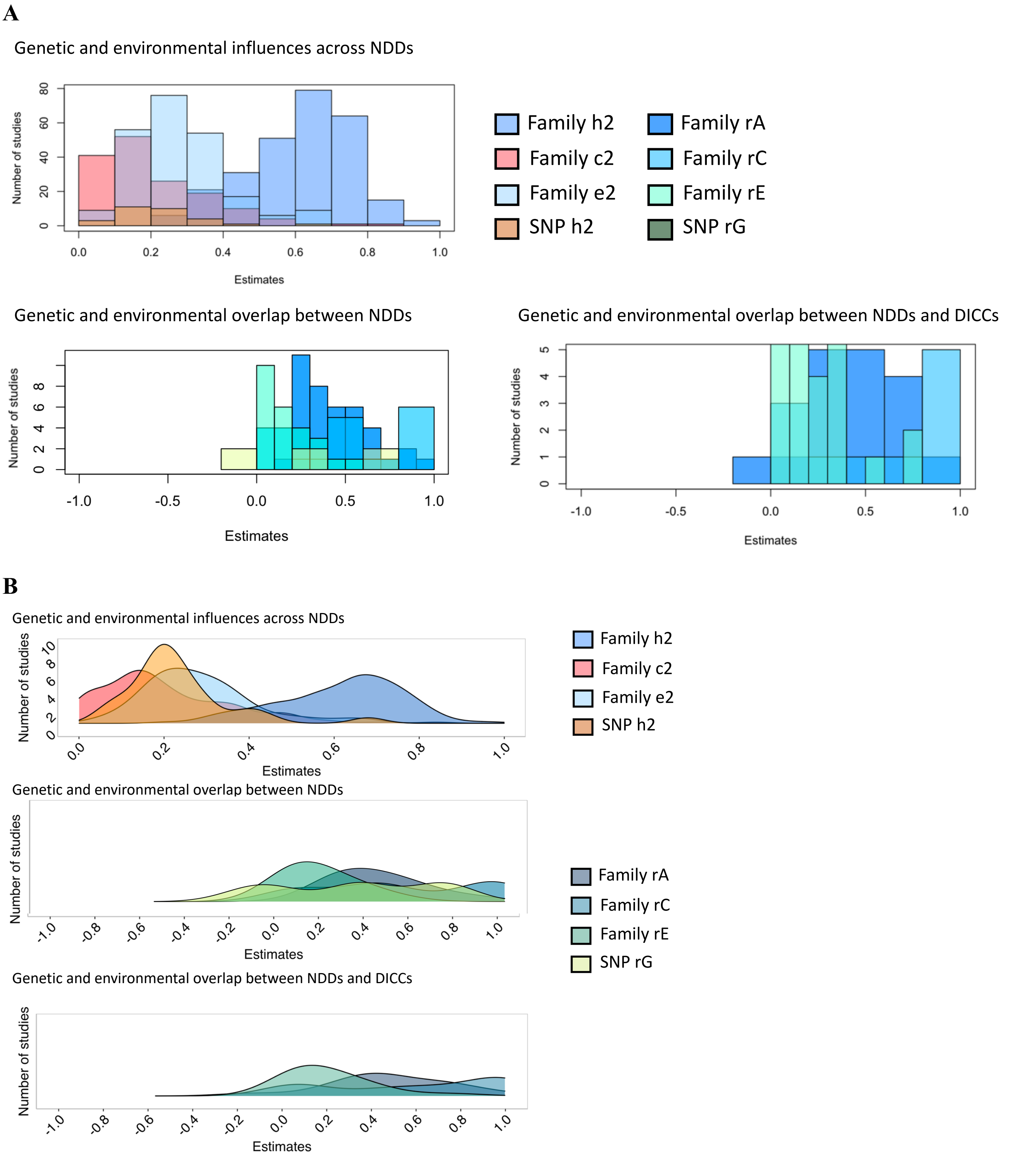

Supplementary Figure 1. Distribution of estimates. Panel **A** presents distribution of heritability (h2), shared (c2) and nonshared (e2) environmental influences on neurodevelopmental disorders (NDDs (top panel), as well as genetic (rA/rG), shared (rC) and nonshared (rE) environmental correlations between NDDs (right bottom panel) and between NDDs and disruptive, impulse control and conduct disorders (DICCs) (left bottom panel). Panel **B** presents density plot of heritability and environmental influences on NDDs (top panel), as well as genetic and environmental correlations between NDDs (middle panel) and between NDDs and DICCs (bottom panel).

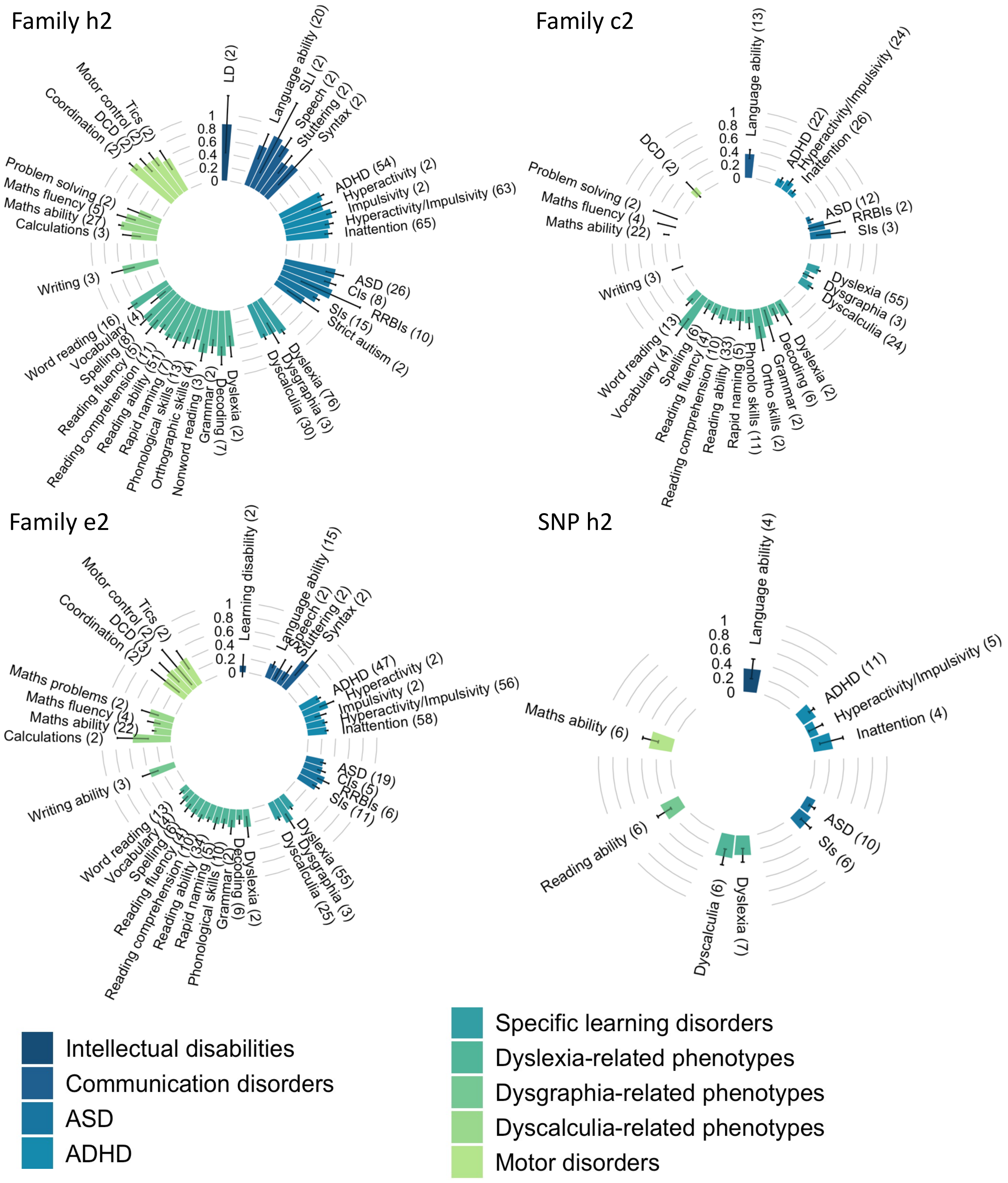

Supplementary Figure 2. Heritability (h2), shared (c2) and nonshared (e2) environmental influences on specific phenotypes within neurodevelopmental disorders (NDDs) categories. Error bars signify standard errors of the grand estimates of heritability and environmental influences. Numbers in brackets denote the number of studies identified that provided estimates for specific phenotypes.

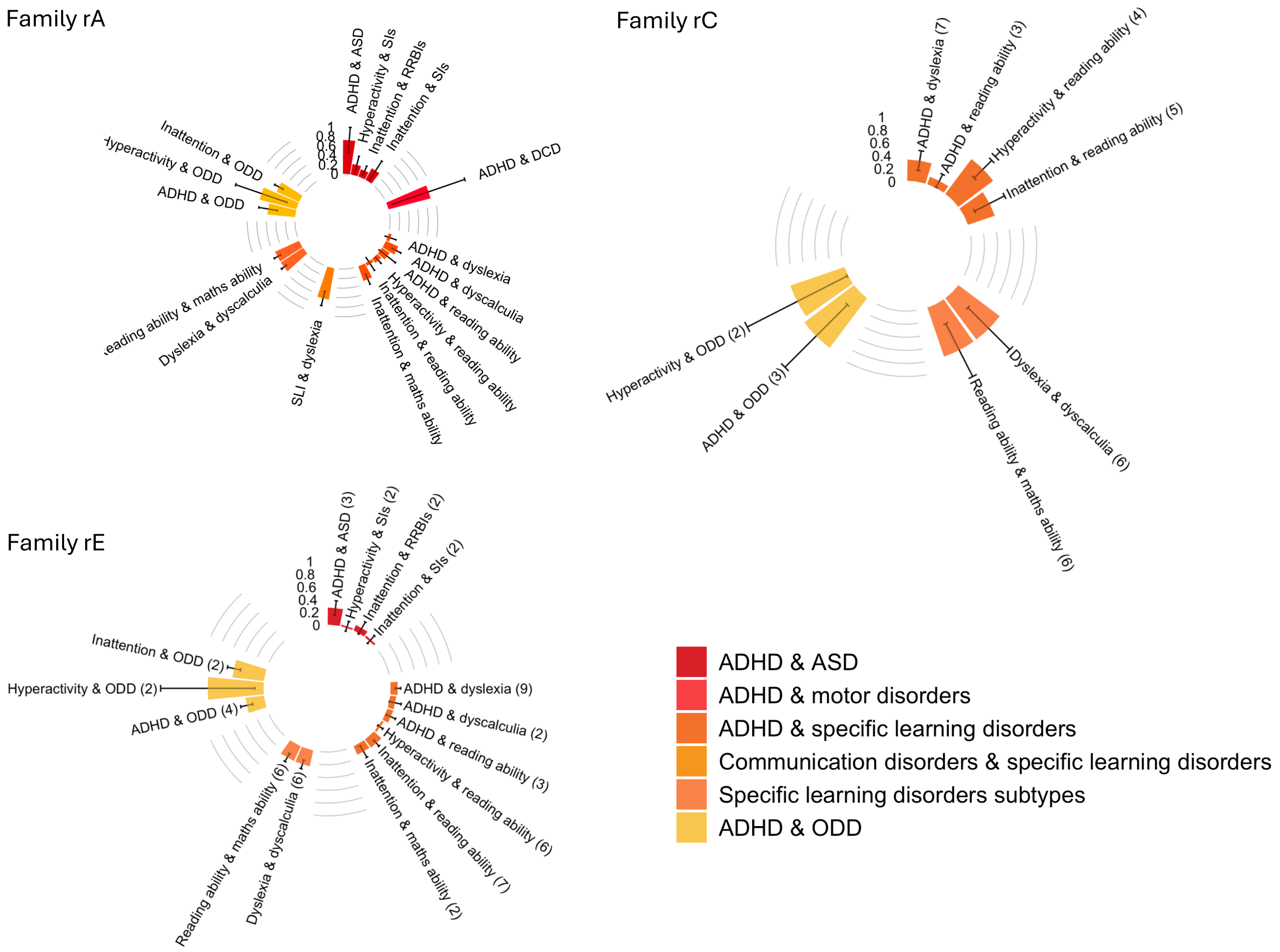

Supplementary Figure 3. Genetic (rA), shared (rC) and nonshared (rE) environmental overlap between specific phenotypes within the neurodevelopmental disorders (NDDs) category and between specific phenotypes within the NDDs and disruptive, impulse control and conduct disorders (DICCs) category. Error bars signify standard errors of the grand estimates of genetic and environmental correlation. Numbers in brackets denote the number of studies identified that provided estimates for specific phenotypes.

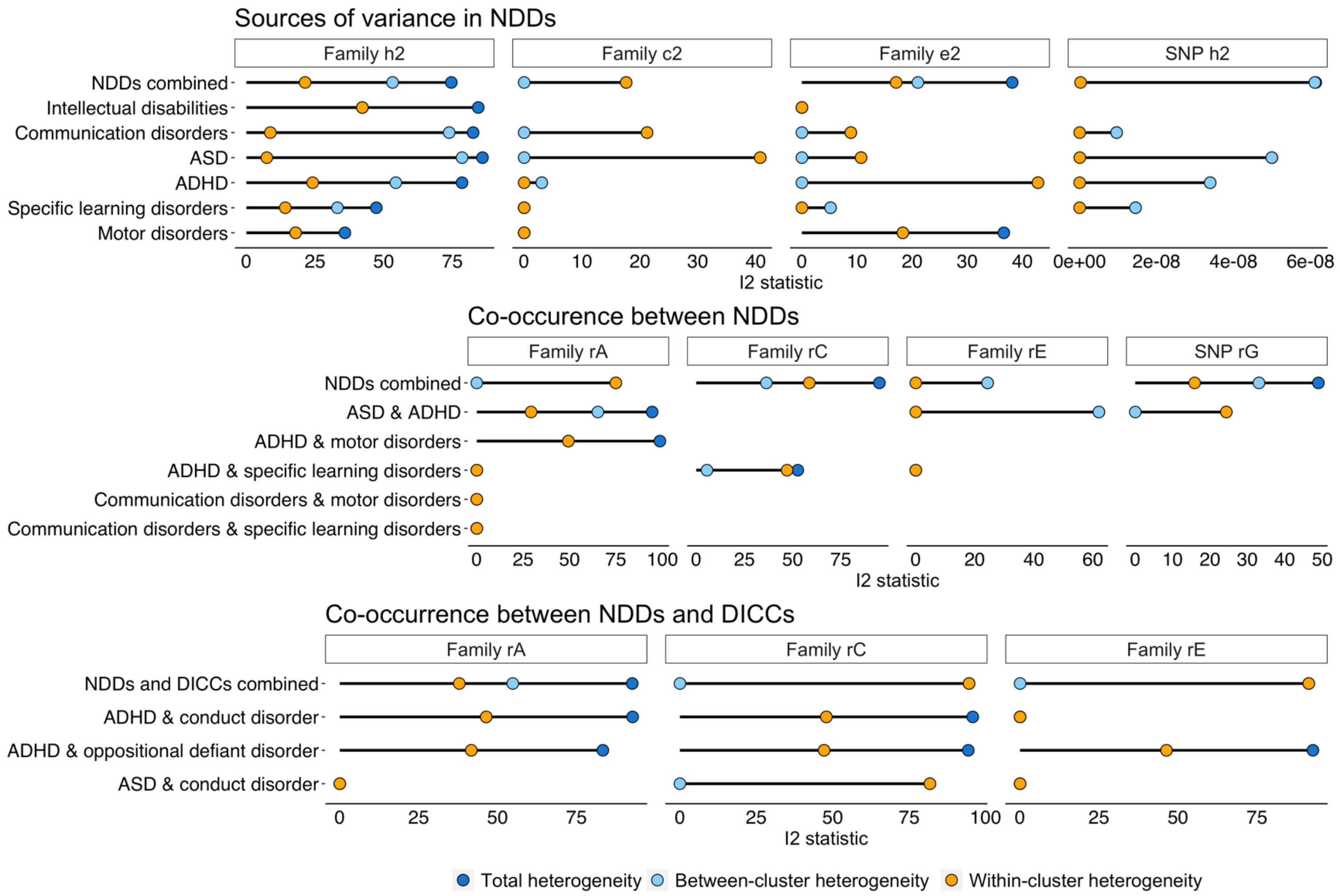

Supplementary Figure 4. Variance in heritability (h2), shared (c2) and nonshared (e2) environmental influences on neurodevelopmental disorders (NDDs) (top panel), variance in genetic (rA/rG), shared (rC) and nonshared (rE) environmental correlations between NDDs (middle panel) and variance in genetic and environmental correlations between NDDs and disruptive, impulse control and conduct disorders (DICCs) that can be attributed to heterogeneity (the I2 statistic).

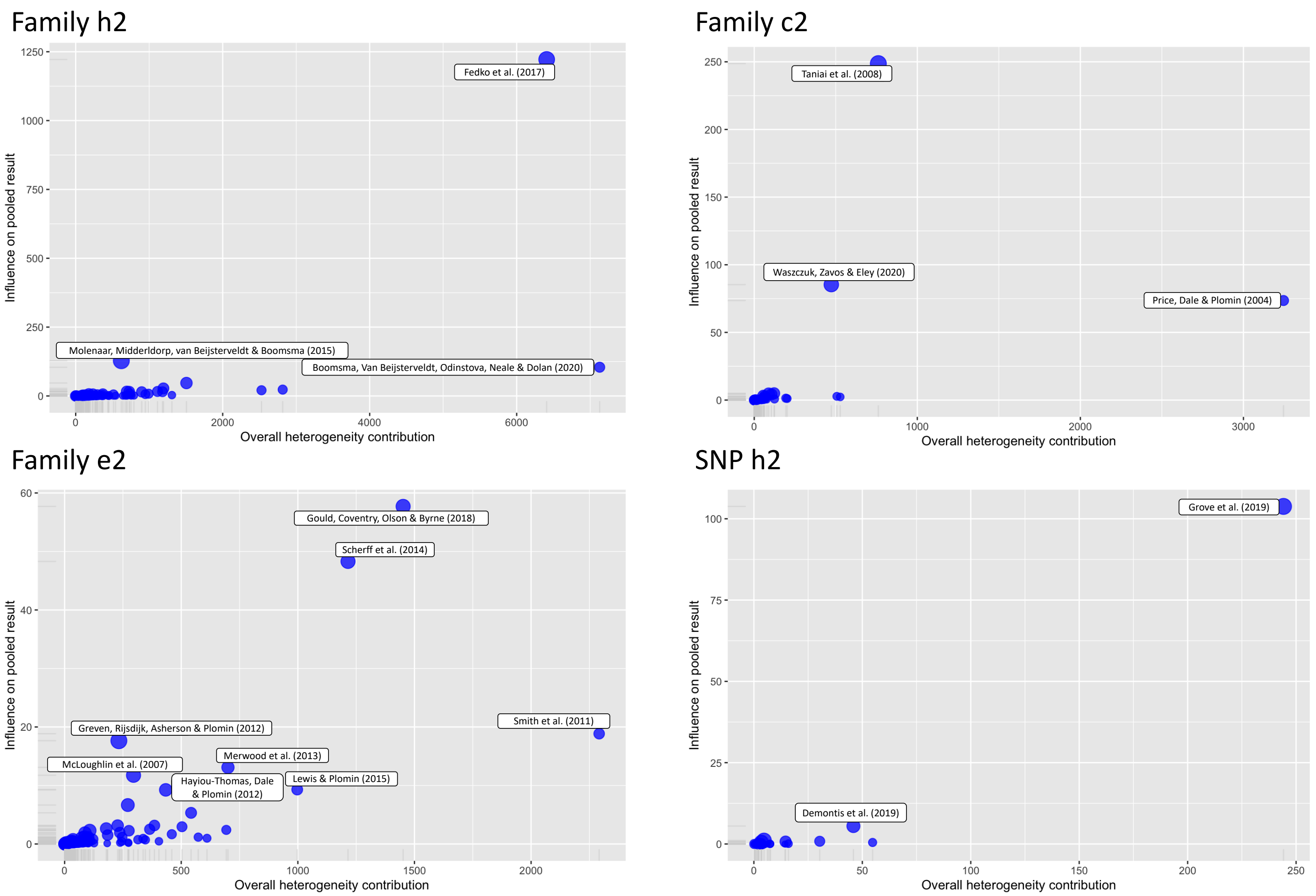

Supplementary Figure 5. Results of the influential cases identification analysis. The baujat plots present studies determined to have a significant impact on the grand estimates of heritability (h2), shared (c2) and nonshared (e2) environmental influences on neurodevelopmental disorders (NDDs) and/or heterogeneity of estimates.

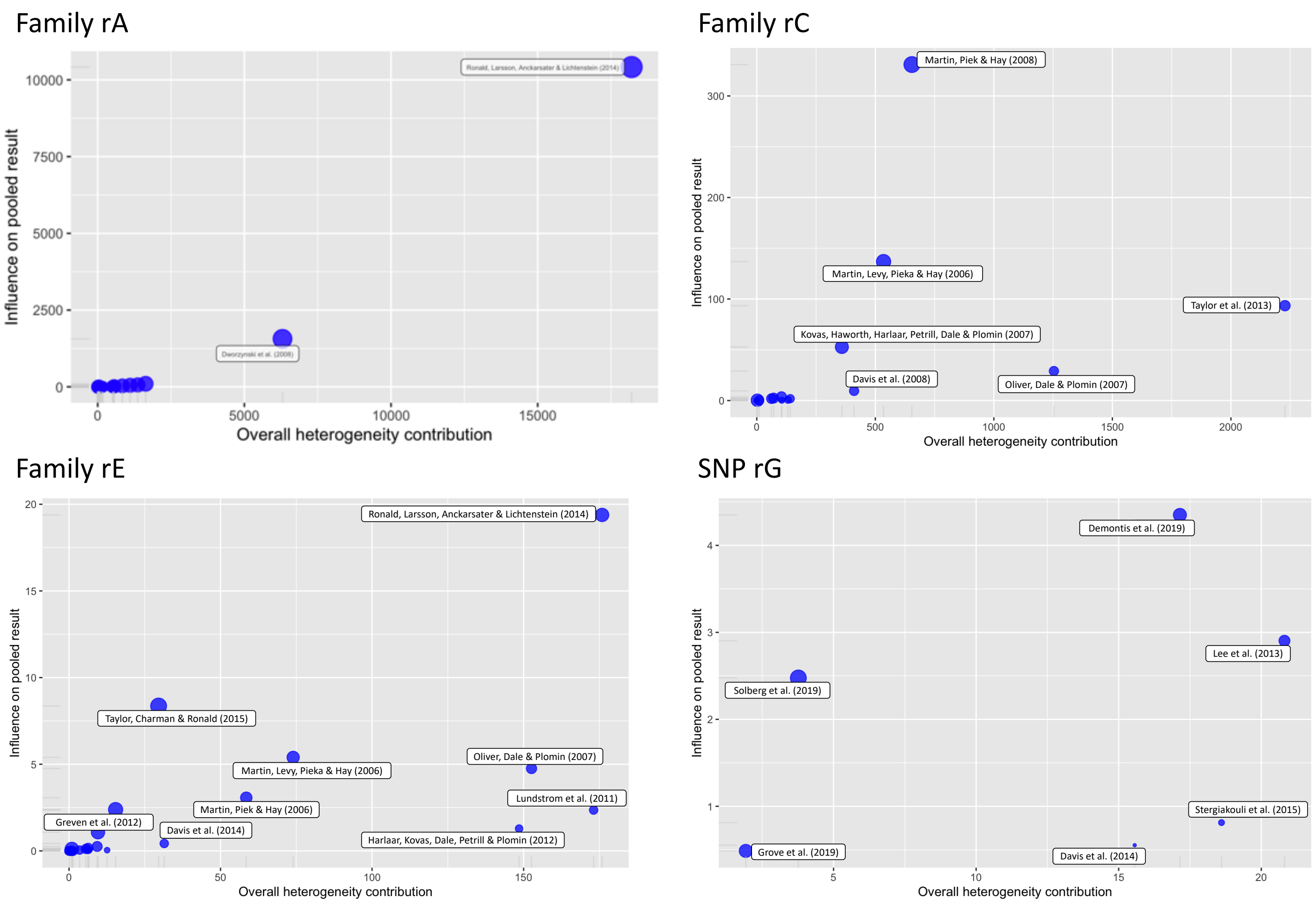

Supplementary Figure 6. Results of the influential cases identification analysis. The baujat plots present studies determined to have a significant impact on the grand estimates of genetic (rA), shared (rC) and nonshared (rE) environmental overlap between neurodevelopmental disorders (NDDs) and/or heterogeneity of estimates.

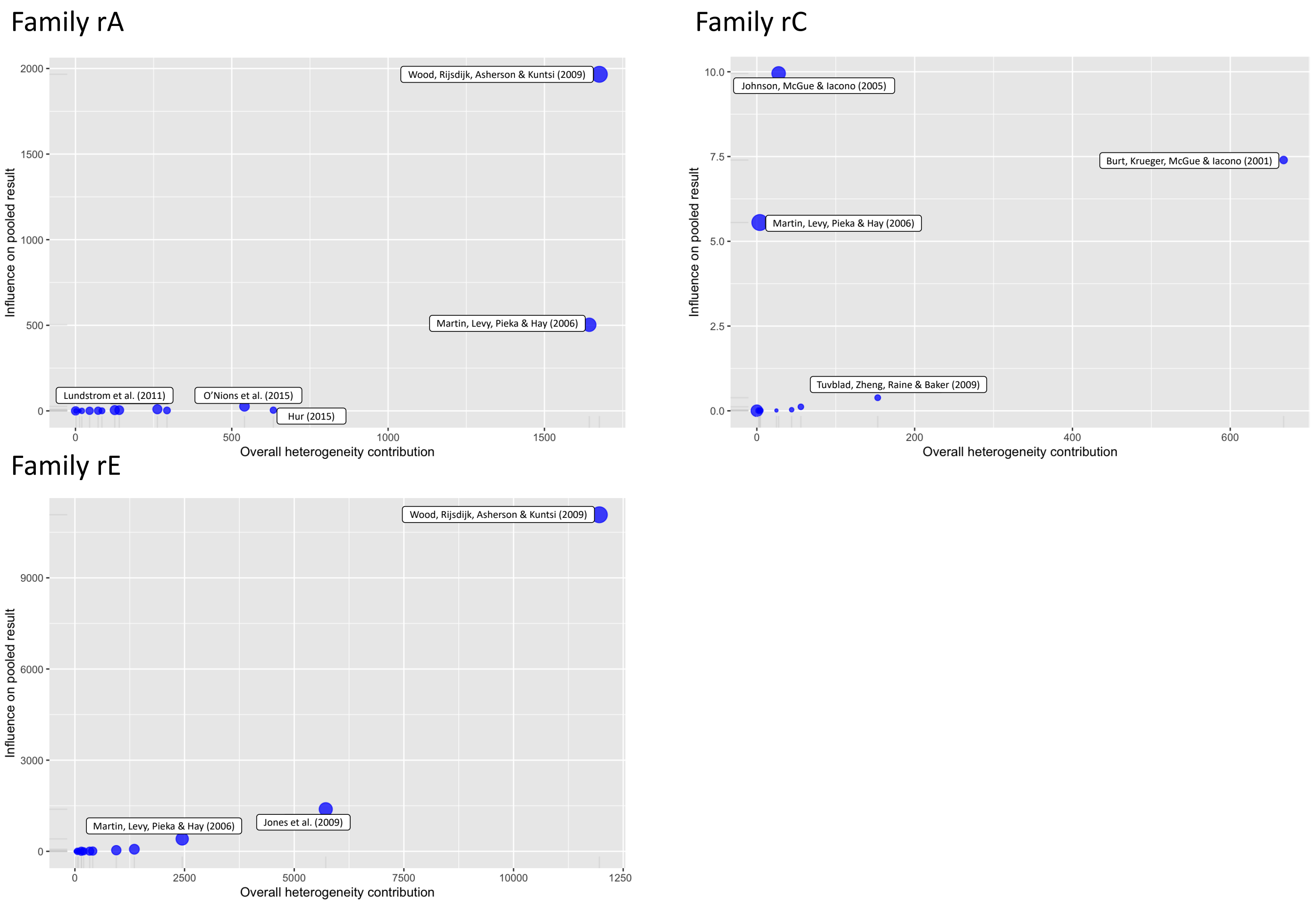

Supplementary Figure 7. Results of the influential cases identification analysis. The baujat plots present studies determined to have a significant impact on the grand estimates of genetic (rA), shared (rC) and nonshared (rE) environmental overlap between neurodevelopmental disorders (NDDs) and disruptive, impulse control and conduct disorders (DICCs) and/or heterogeneity of estimates.

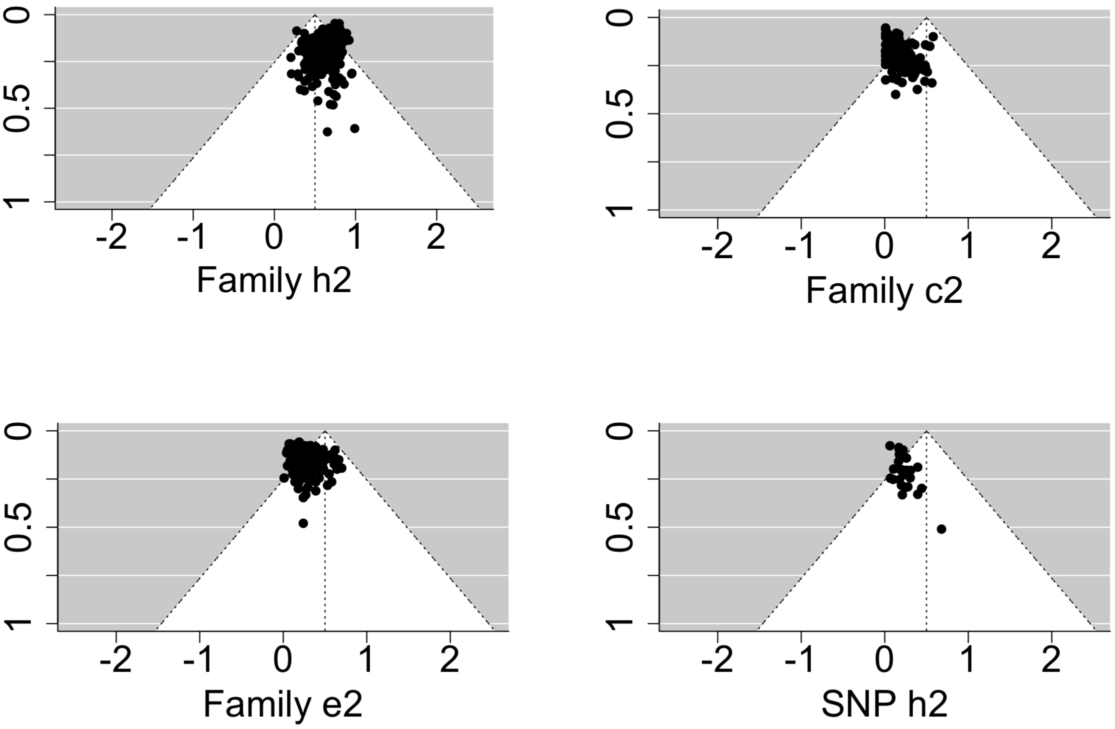

Supplementary Figure 8. Funnel plots involving all studies addressing heritability (h2), shared (c2) and nonshared (e2) environmental influences on neurodevelopmental disorders (NDDs).

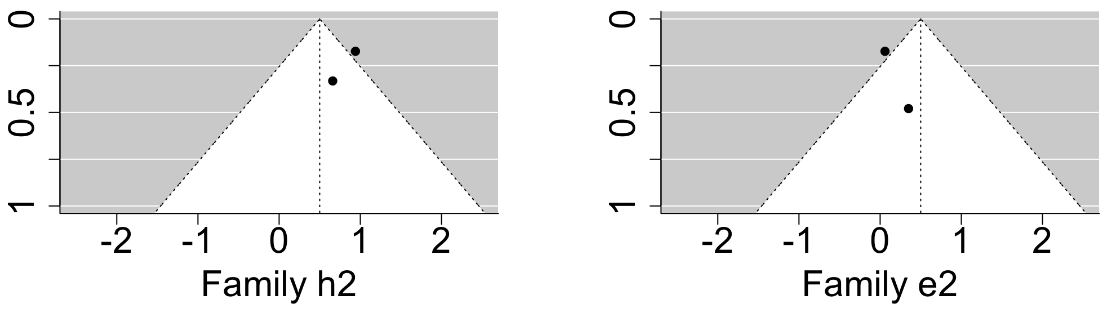

Supplementary Figure 9. Funnel plots involving all studies addressing heritability (h2) and nonshared (e2) environmental influences on intellectual disabilities.

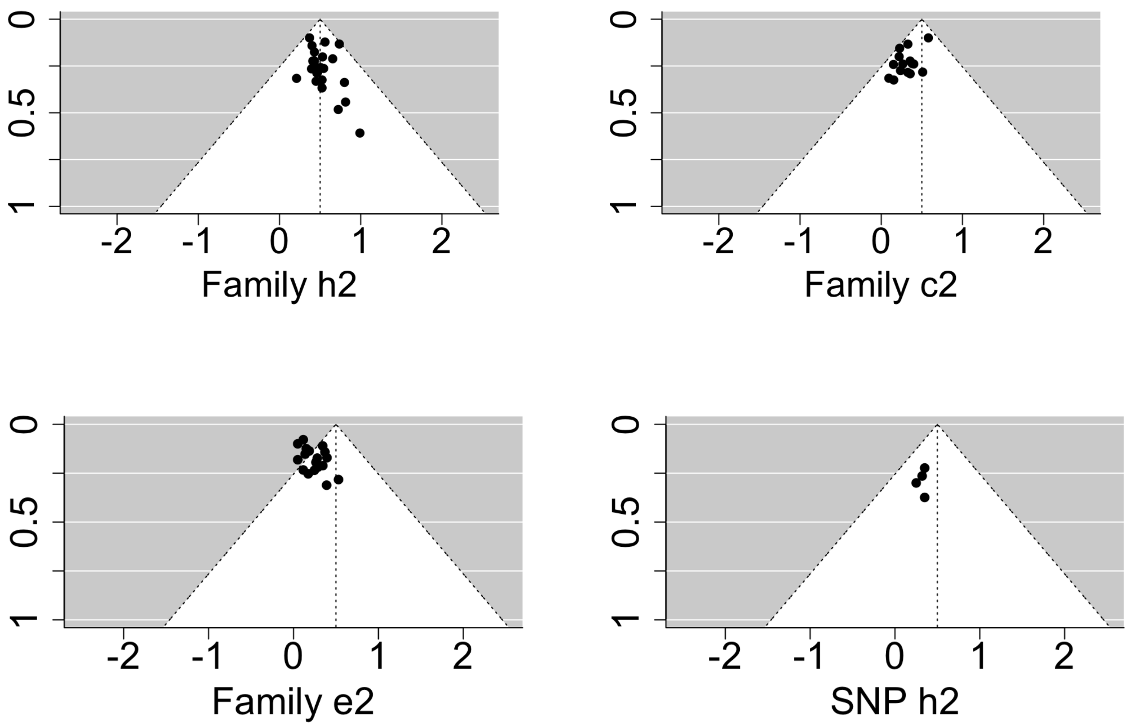

Supplementary Figure 10. Funnel plots involving all studies addressing heritability (h2), shared (c2) and nonshared (e2) environmental influences on communication disorders.

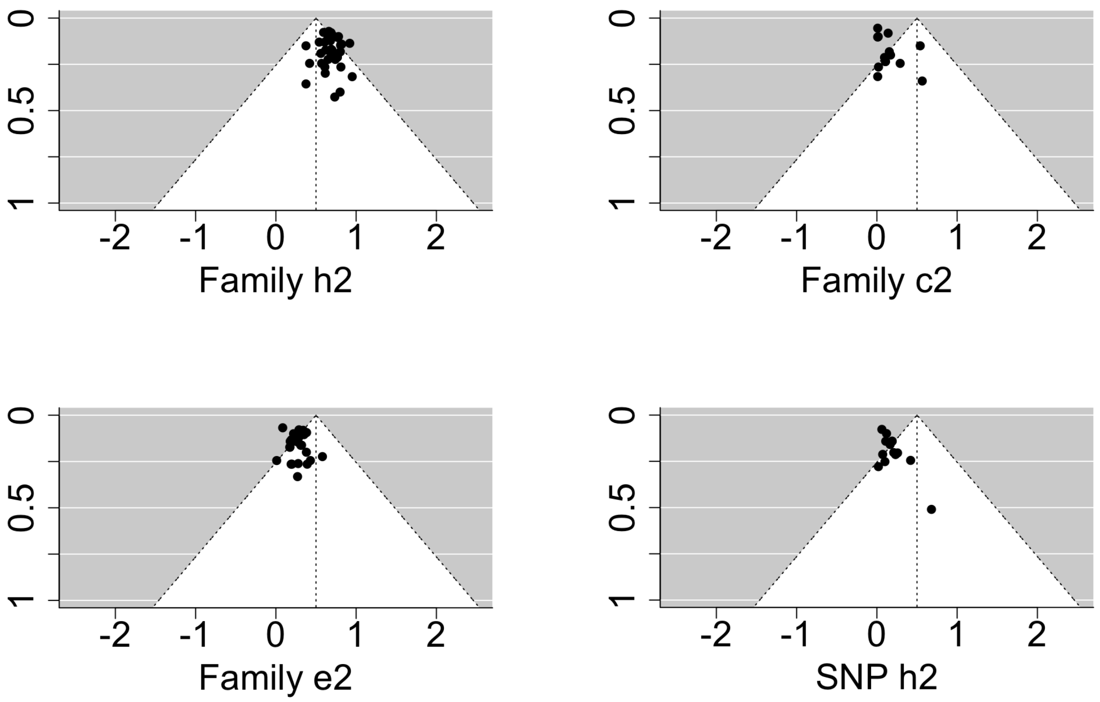

Supplementary Figure 11. Funnel plots involving all studies addressing heritability (h2), shared (c2) and nonshared (e2) environmental influences on ASD.

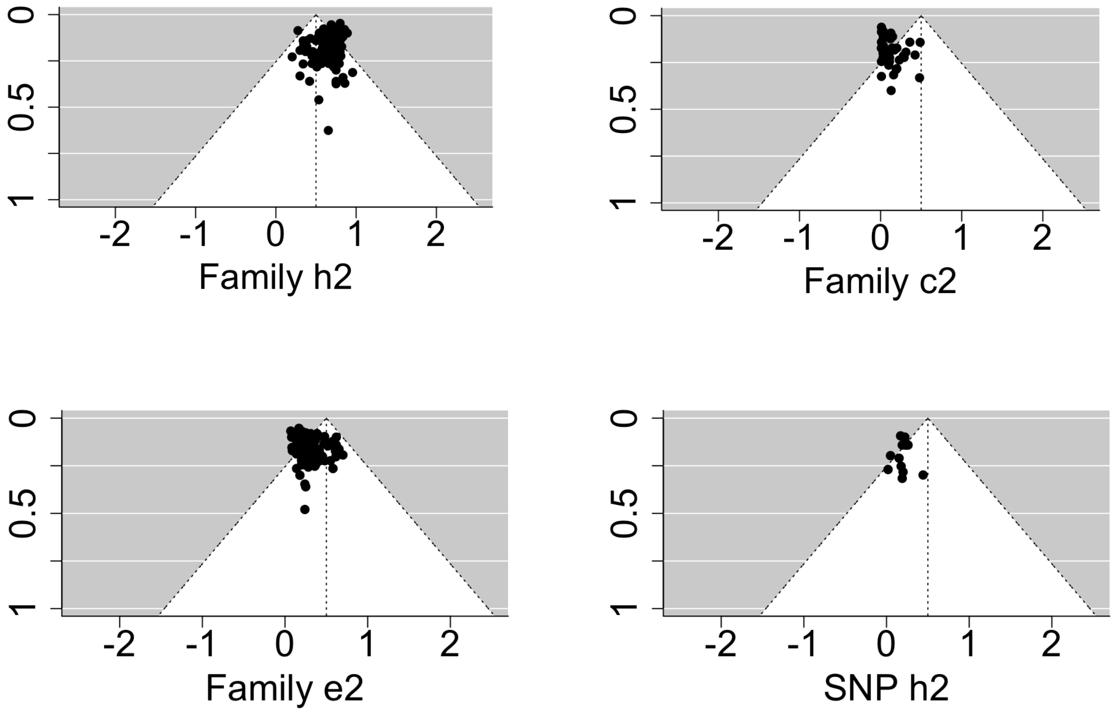

Supplementary Figure 12. Funnel plots involving all studies addressing heritability (h2), shared (c2) and nonshared (e2) environmental influences on ADHD.

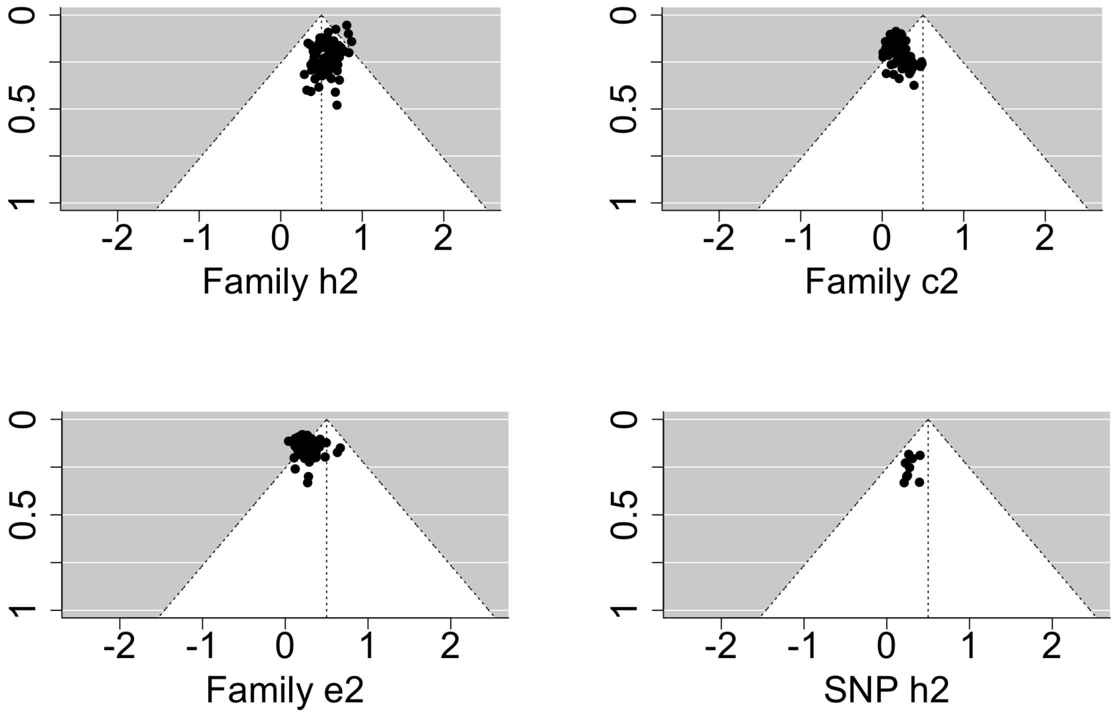

Supplementary Figure 13. Funnel plots involving all studies addressing heritability (h2), shared (c2) and nonshared (e2) environmental influences on specific learning disorders.

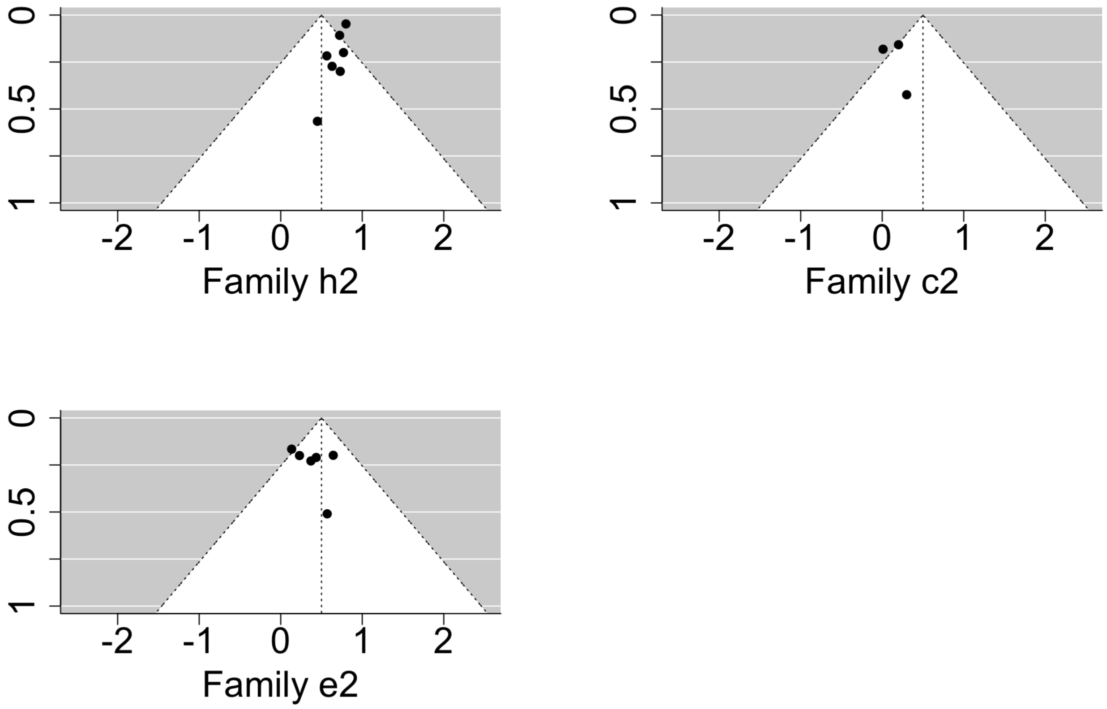

Supplementary Figure 14. Funnel plots involving all studies addressing heritability (h2), shared (c2) and nonshared (e2) environmental influences on motor disorders.

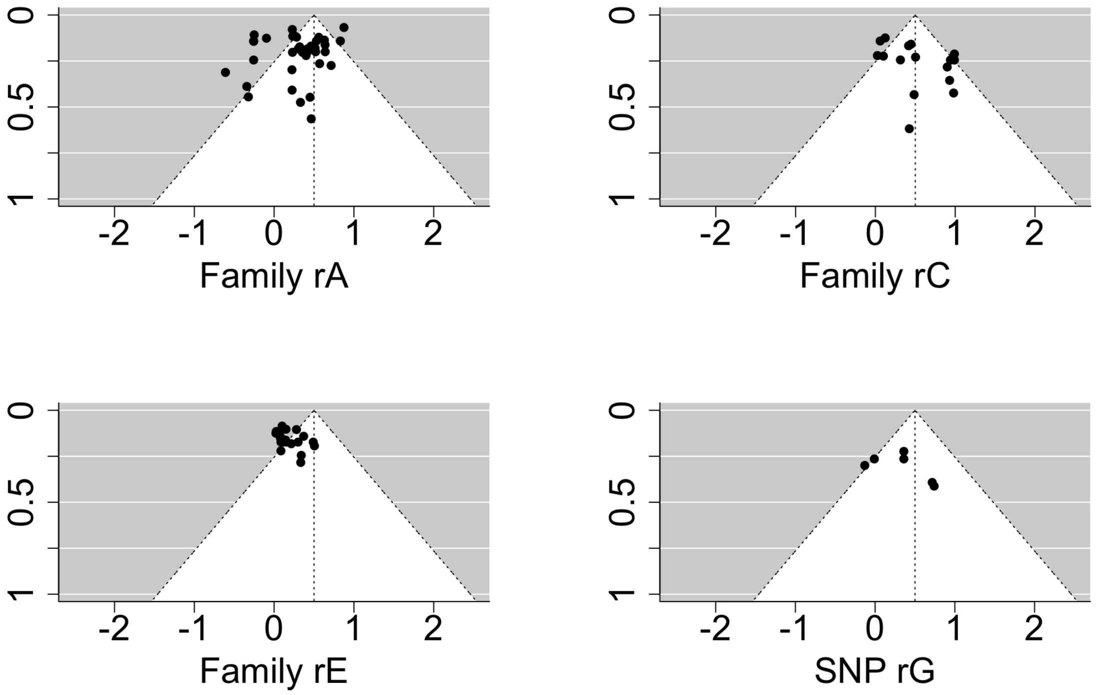

Supplementary Figure 15. Funnel plots involving all studies addressing genetic (rA), shared (rC) and nonshared (rE) environmental overlap between neurodevelopmental disorders (NDDs).

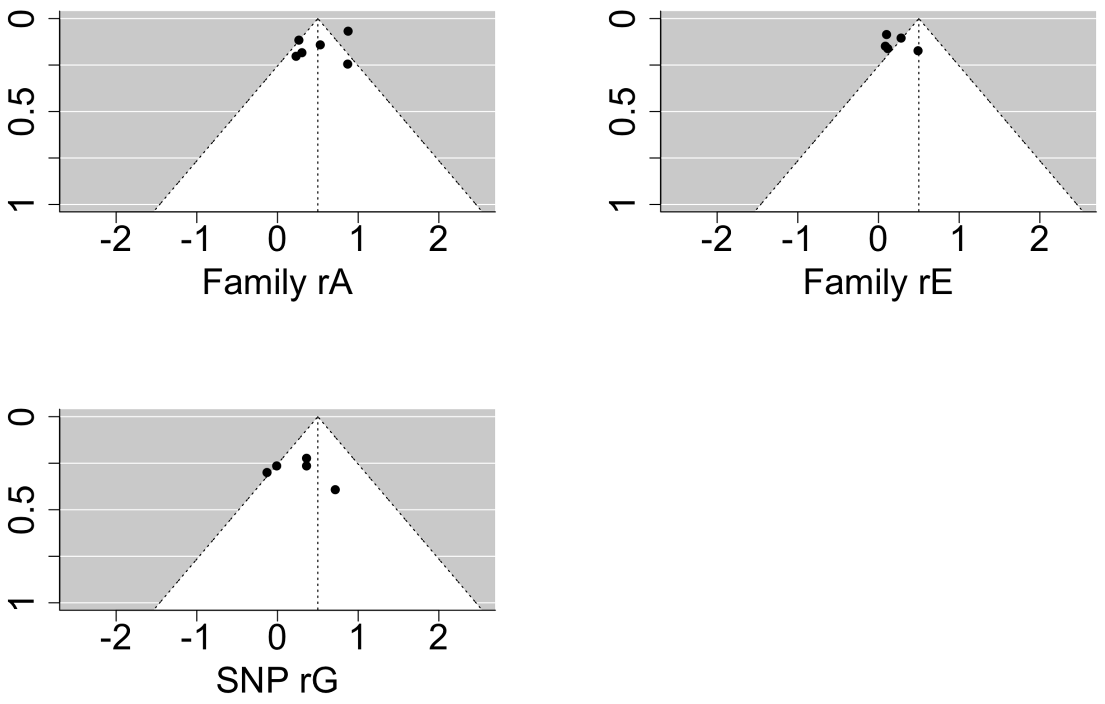

Supplementary Figure 16. Funnel plots involving all studies addressing genetic (rA), and nonshared (rE) environmental overlap between ASD & ADHD.

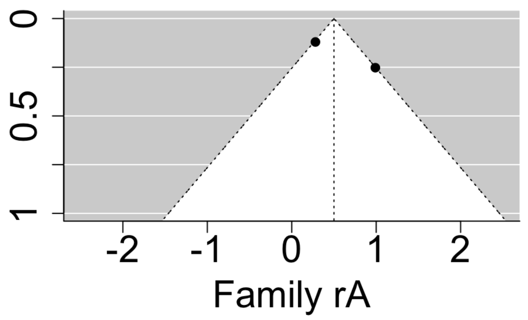

Supplementary Figure 17. Funnel plots involving all studies addressing genetic overlap (rA) between ADHD & motor disorders.

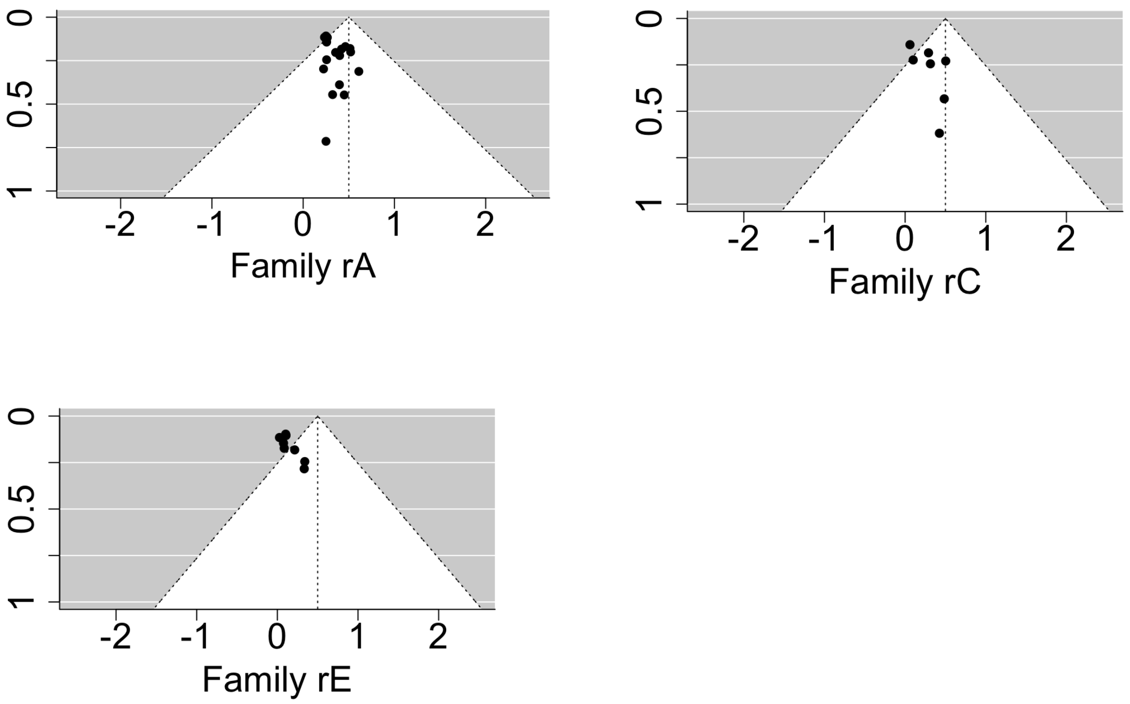

Supplementary Figure 18. Funnel plots involving all studies addressing genetic (rA), shared (rC) and nonshared (rE) environmental overlap between ADHD & specific learning disorders.

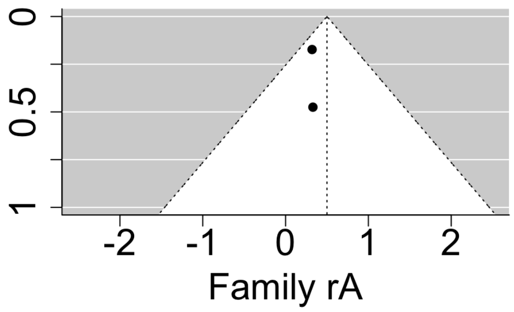

Supplementary Figure 19. Funnel plots involving all studies addressing genetic overlap (rA) between communication disorders & motor disorders.

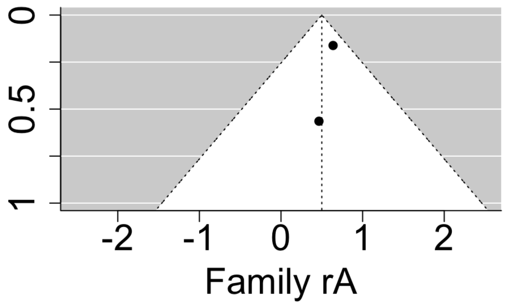

Supplementary Figure 20. Funnel plots involving all studies addressing genetic overlap (rA) between communication disorders & specific learning disorders.

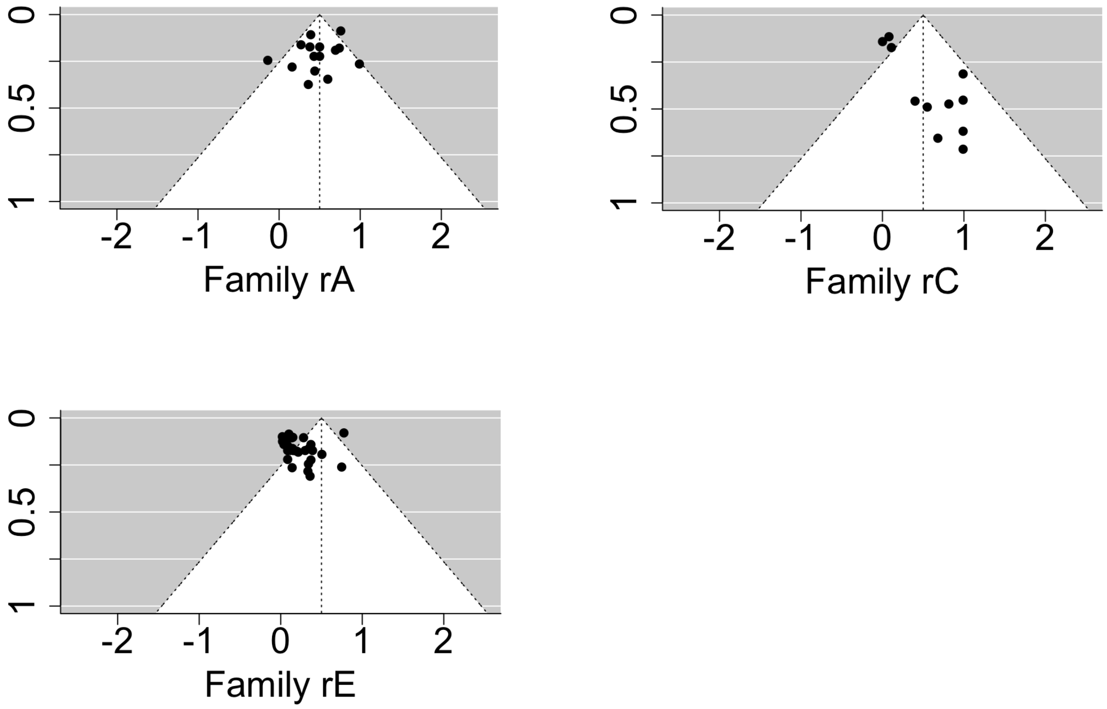

Supplementary Figure 21. Funnel plots involving all studies addressing genetic (rA), shared (rC) and nonshared (rE) environmental overlap between neurodevelopmental disorders (NDDs) and disruptive, impulse control and conduct disorders (DICCs).

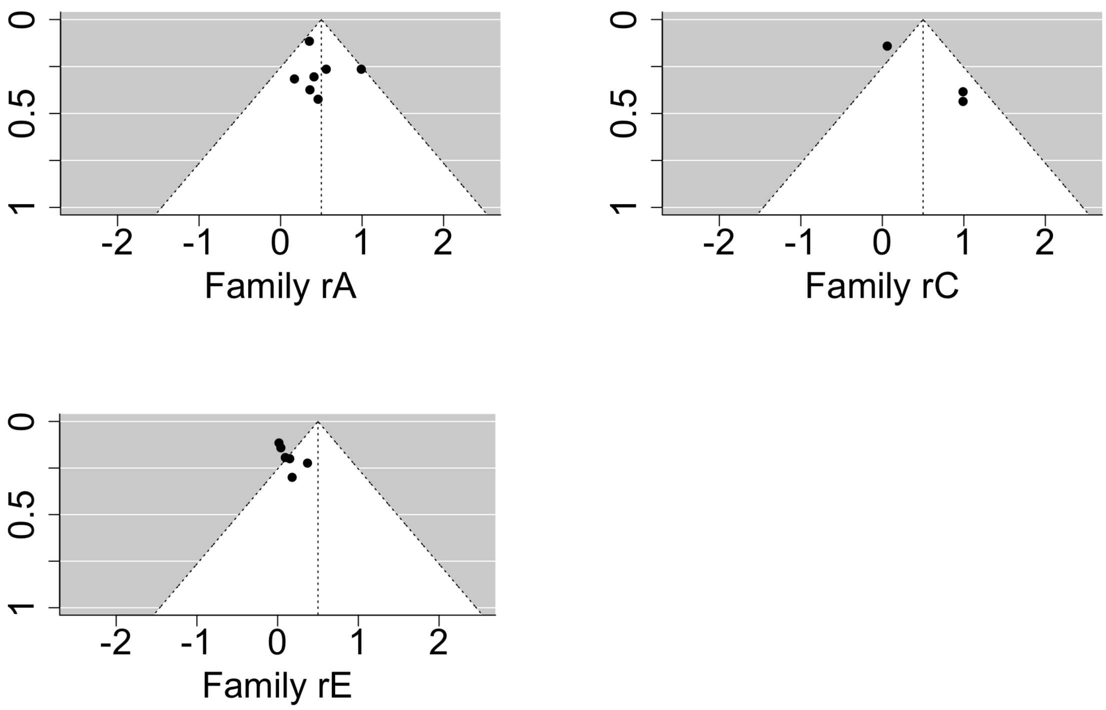

Supplementary Figure 22. Funnel plots involving all studies addressing genetic (rA), shared (rC) and nonshared (rE) environmental overlap between ADHD & conduct disorder.

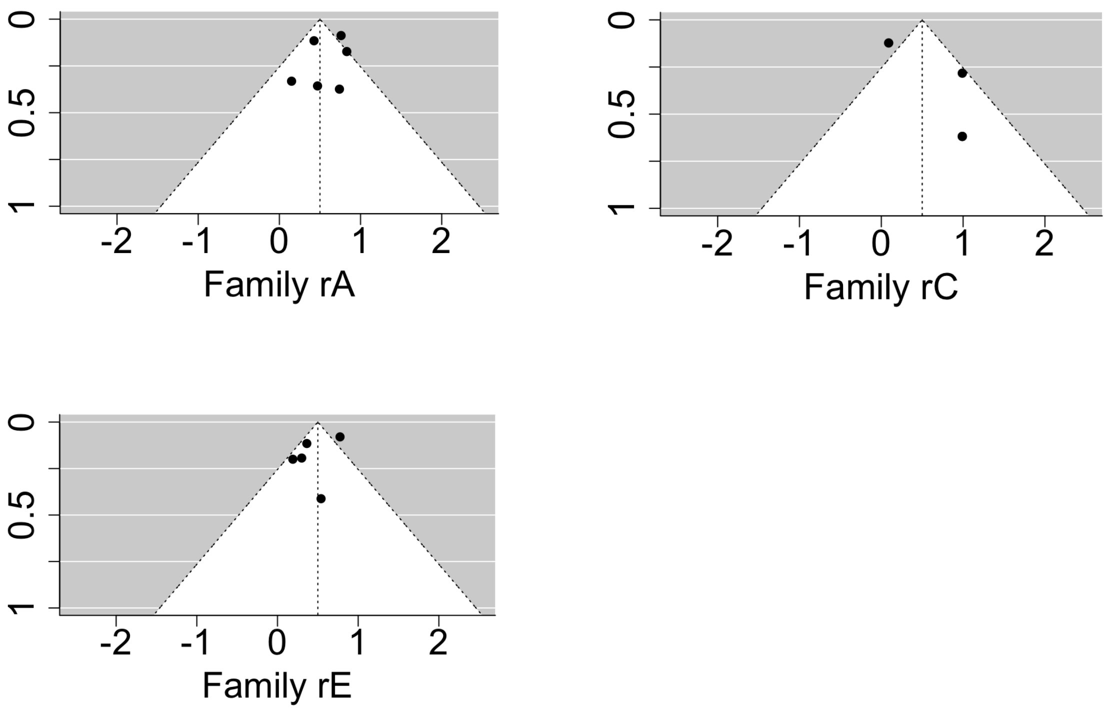

Supplementary Figure 23. Funnel plots involving all studies addressing genetic (rA), shared (rC) and nonshared (rE) environmental overlap between ADHD & oppositional defiant disorder.

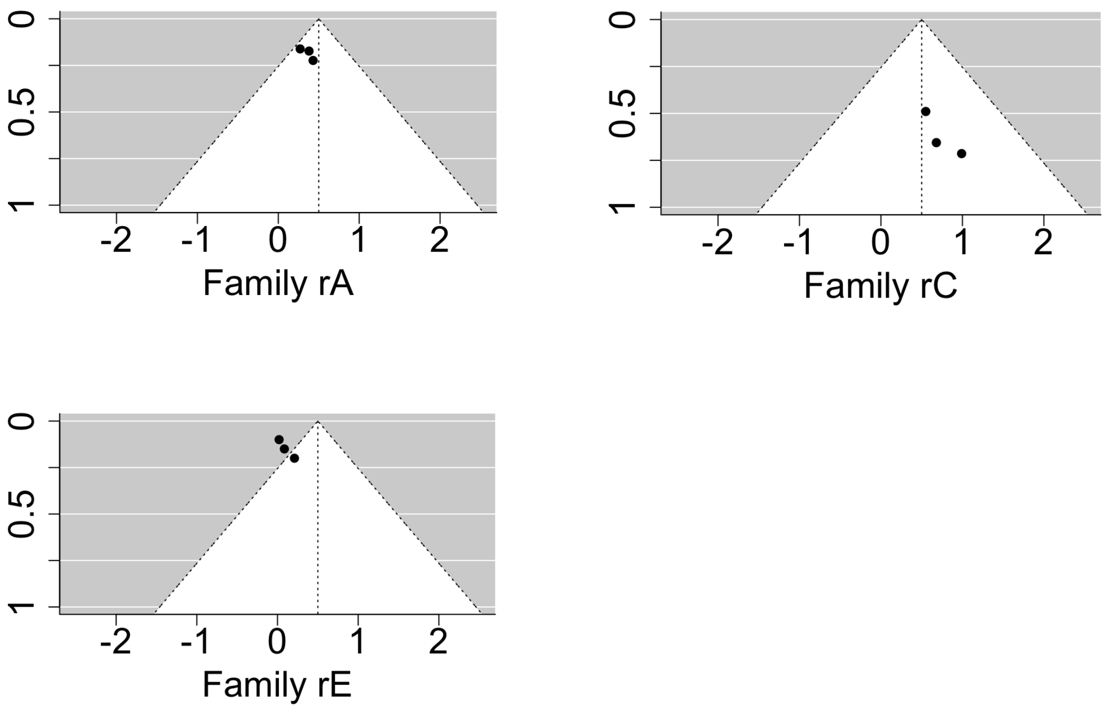

Supplementary Figure 24. Funnel plots involving all studies addressing genetic (rA), shared (rC) and nonshared (rE) environmental overlap between ASD & conduct disorder.

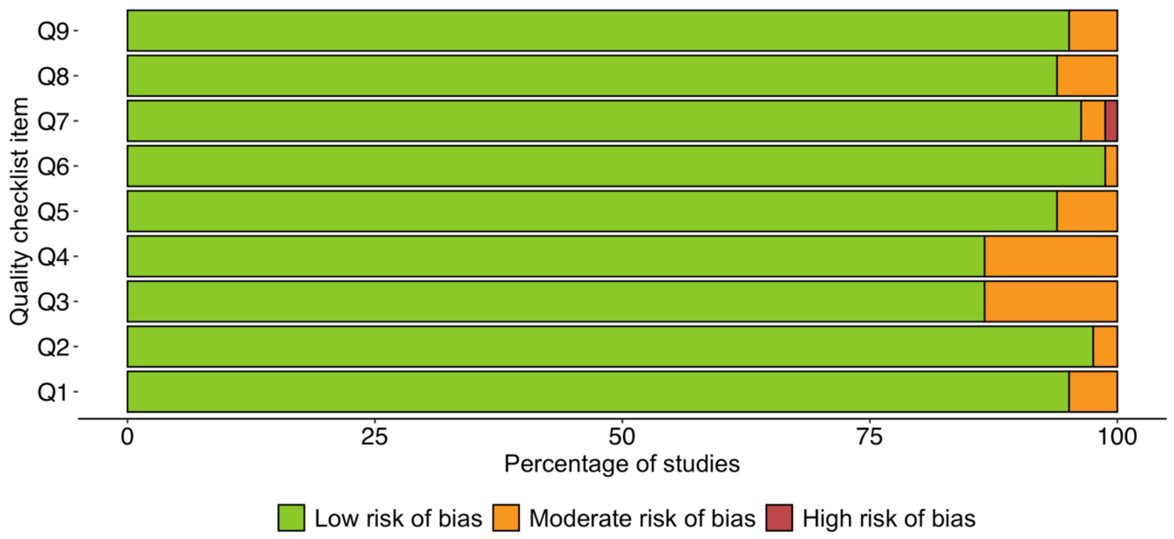

Supplementary Figure 25. Results of the study quality assessment, illustrated as the percentage of studies showing low, moderate and high risk of bias.

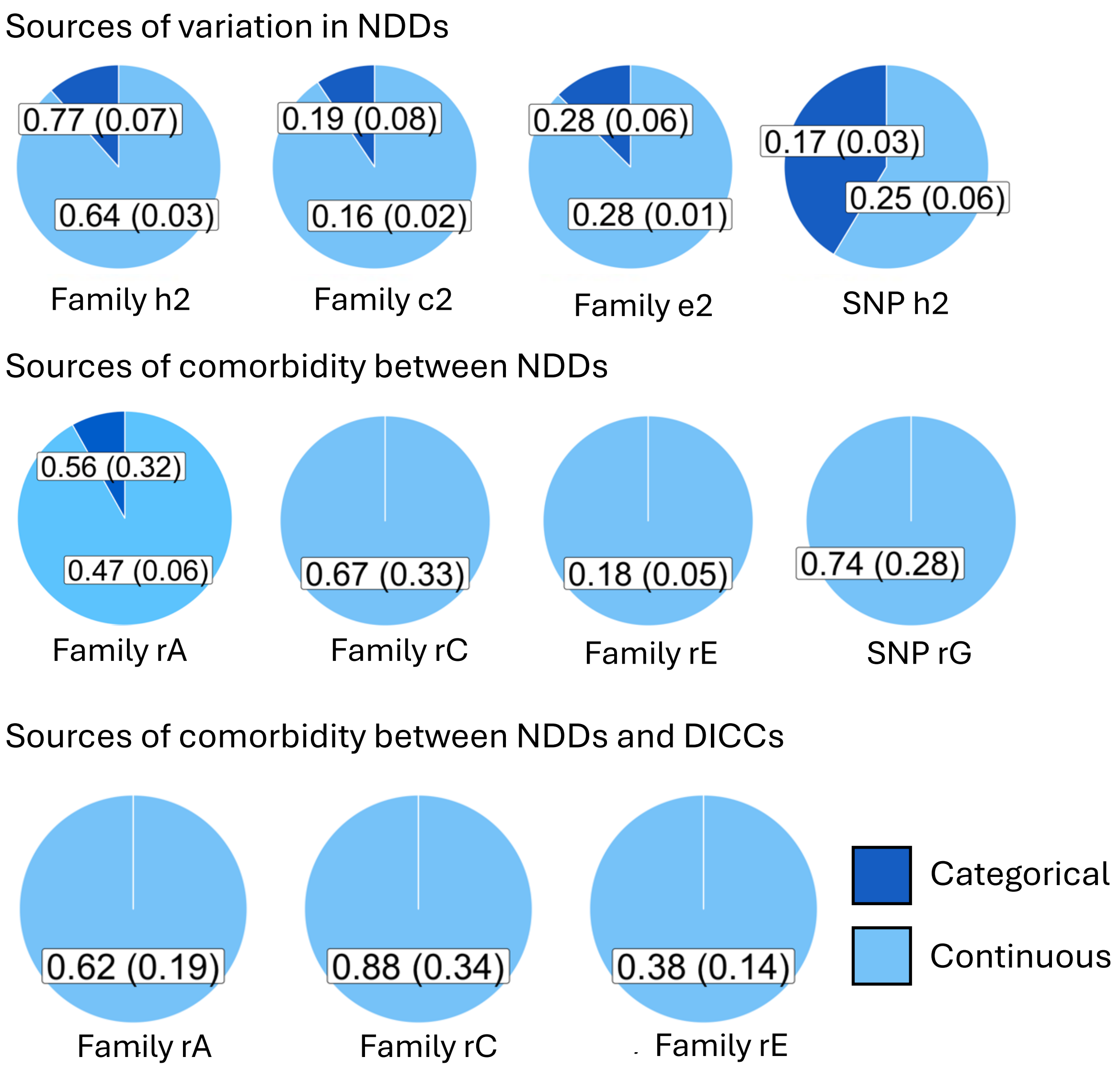

Supplementary Figure 26. Heritability (h2), shared (c2) and nonshared (e2) environmental influences on neurodevelopmental disorders (NDDs) (top panel), genetic (rA/rG), shared (rC) and nonshared (rE) environmental overlap between NDDs (middle panel) and genetic and environmental overlap between NDDs and disruptive, impulse control and conduct disorders (DICCs) (bottom panel), stratified by measurement scales, i.e., categorical versus continuous measurement.

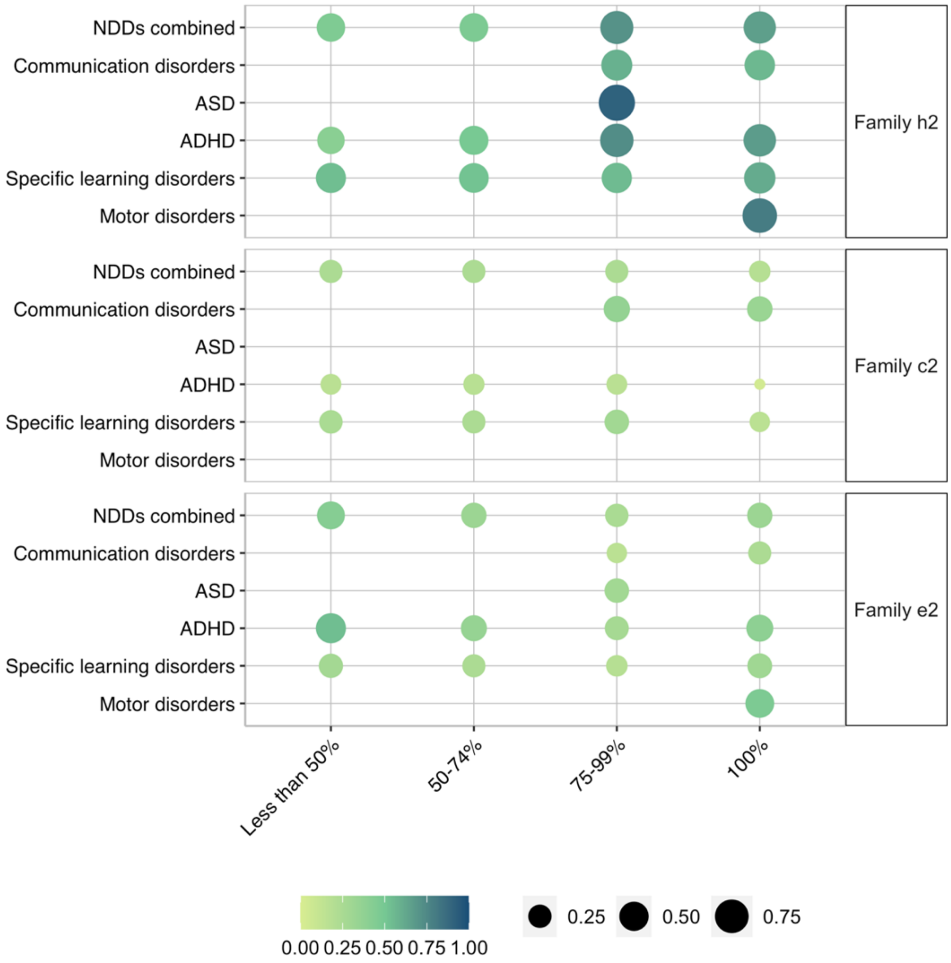

Supplementary Figure 27. Changes in family-based heritability (h2), shared (c2) and nonshared (e2) environmental influences on neurodevelopmental disorders (NDDs), as a function of sample ancestral composition. Given the general lack of diversity, ancestral composition could only be quantified, and consequently meta-analysed, as percentage of the sample being of European ancestry, different categories based on these percentages are depicted on the x-axis. Grand estimates of h2, c2 and e2 are reflected in the size and colour intensity of each circle, the larger and darker the circle, the higher the grand estimate.

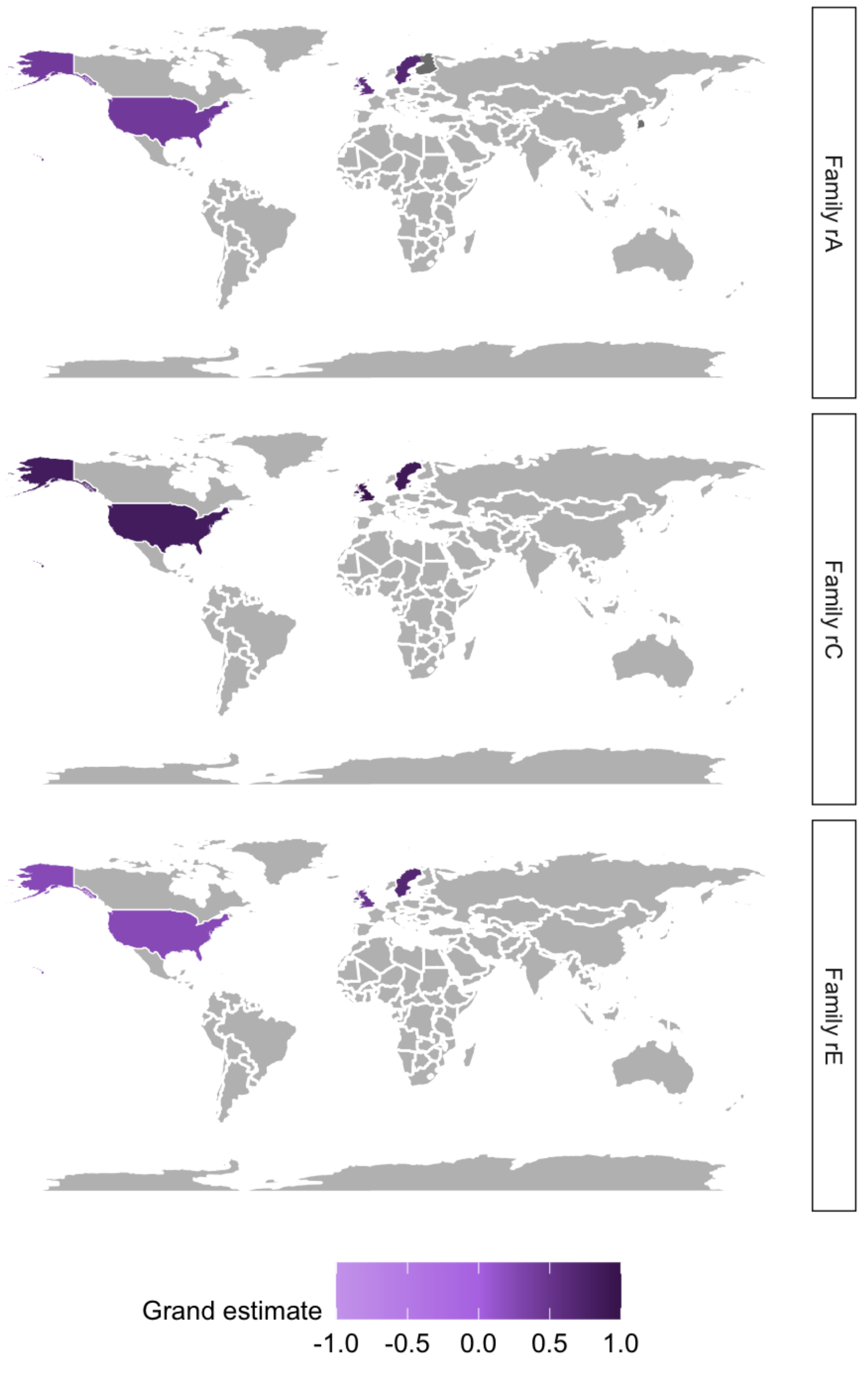

Supplementary Figure 28. Geographical differences in rA, rC and rE between NDDs and disruptive, impulse control and conduct disorders (DICCs). The areas shaded in grey are regions for which not enough relevant studies were identified (<2 studies). The results for c^2 and e^2 as well as rC and rE are discussed in Supplementary Note 1.

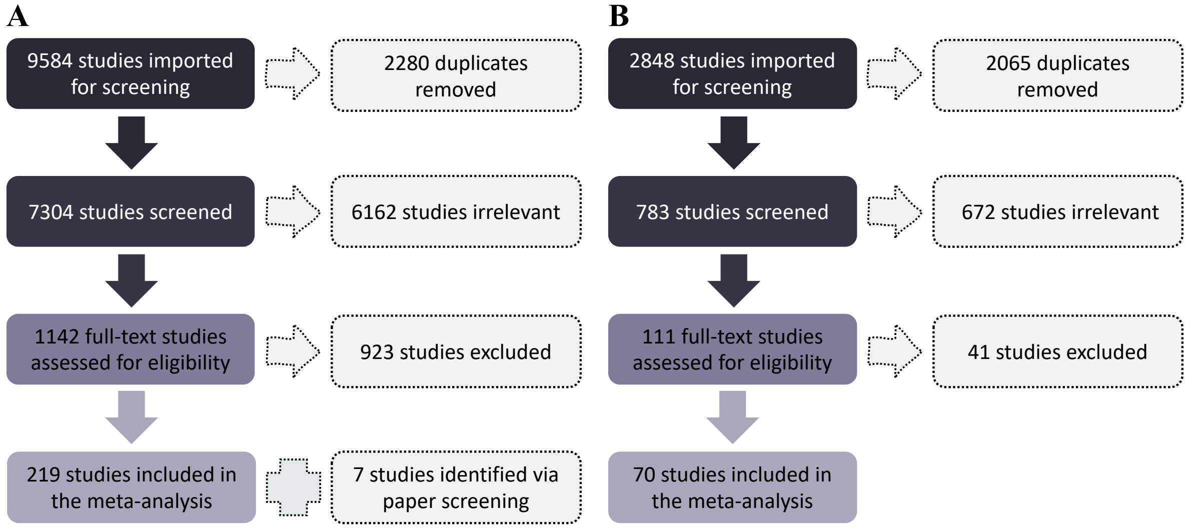

Supplementary Figure 29. Diagram of searches and screening. Panel **A** shows study selection workflow of the primary search and Panel **B** shows workflow of the confirmatory search.

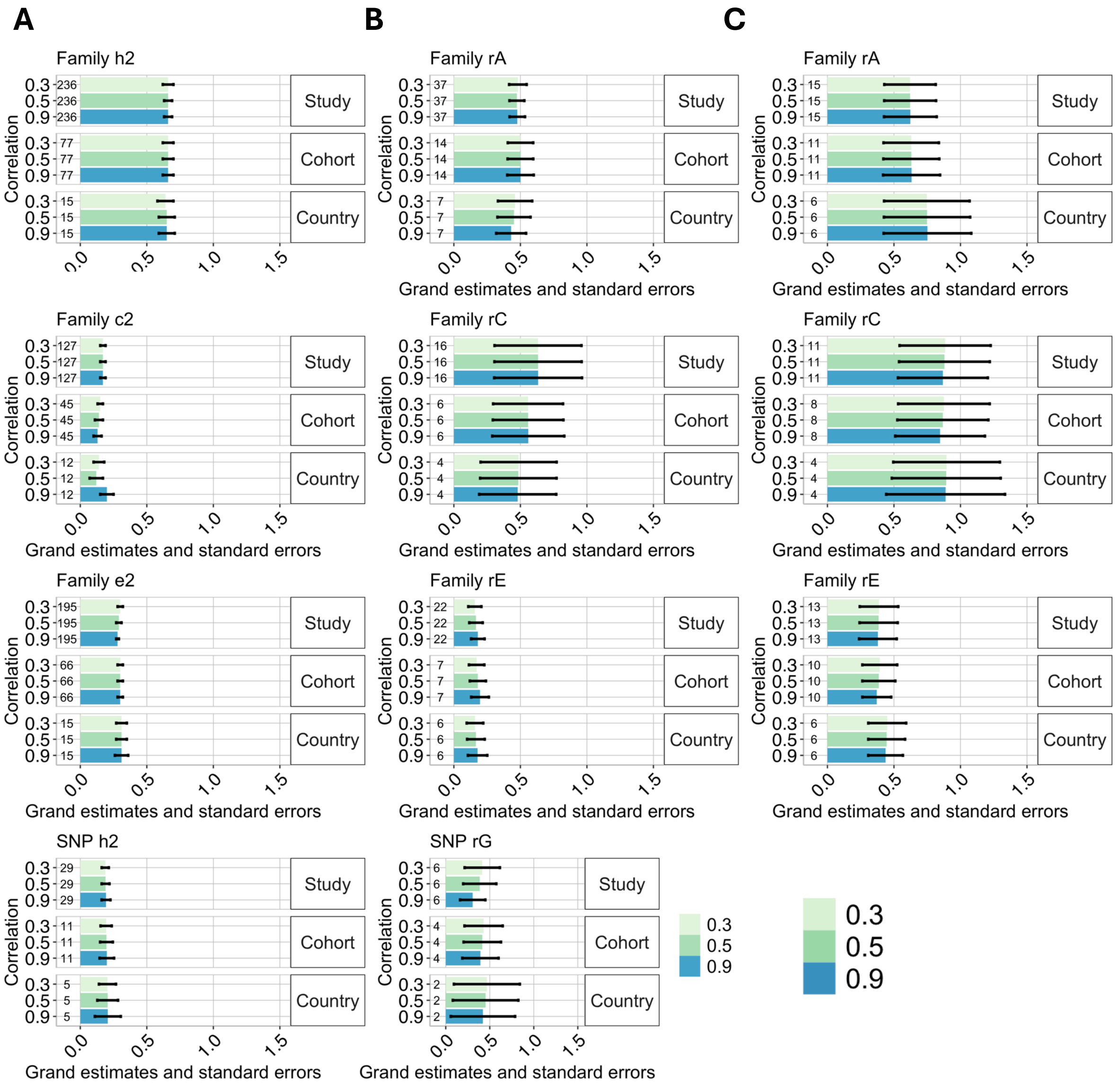

Supplementary Figure 30. Grand heritability (h2), shared (c2) and nonshared (e2) environmental influences across all neurodevelopmental disorders (NDDs) (panel **A**), grand genetic (rA), shared (rC) and nonshared (rE) environmental correlations across all NDDs (panel **B**) and grand genetic and environmental correlations across NDDs and disruptive, impulse control and conduct disorders (DICCs) (panel **C**) obtained using different aggregation techniques, i.e., aggregating by study, cohort, and country, using correlation thresholds of r= 0.3, r= 0.5 and r= 0.9. Error bars signify standard errors of the grand estimates of heritability/environmental influences or genetic/environmental correlation. Numbers preceding bars on the y-axis denote the number of aggregated items.

Supplementary references

1. Lichtenstein, P., Carlström, E., Råstam, M., Gillberg, C. & Anckarsäter, H. The genetics of autism spectrum disorders and related neuropsychiatric disorders in childhood. *Am. J. Psychiatry* **167**, 1357–1363 (2010).
2. McGue, M. & Bouchard, T. J. Adjustment of twin data for the effects of age and sex. *Behav. Genet.* **14**, 325–343 (1984).
3. Abdellaoui, A. *et al.* Genetic correlates of social stratification in Great Britain. *Nat. Hum. Behav.* **3**, 1332–1342 (2019).
4. Lin, L. & Chu, H. Quantifying publication bias in meta-analysis. *Biometrics* **74**, 785–794 (2018).
5. Yang, J., Lee, S. H., Goddard, M. E. & Visscher, P. M. GCTA: A tool for genome-wide complex trait analysis. *Am. J. Hum. Genet.* **88**, 76–82 (2011).
6. Bulik-Sullivan, B. K. *et al.* LD Score regression distinguishes confounding from polygenicity in genome-wide association studies. *Nat. Genet.* **47**, 291–295 (2015).
7. Zeng, J. *et al.* Signatures of negative selection in the genetic architecture of human complex traits. *Nat. Genet.* **50**, 746–753 (2018).
8. Kmet, L. M., Cook, L. S. & Lee, R. C. Standard quality assessment criteria for evaluating primary research papers from a variety of fields. (2004).
9. Davis, L. K. *et al.* Partitioning the heritability of Tourette syndrome and obsessive compulsive disorder reveals differences in genetic architecture. *PLoS Genet.* **9**, e1003864 (2013).
10. Du Rietz, E. *et al.* Overlap between attention-deficit hyperactivity disorder and neurodevelopmental, externalising and internalising disorders: separating unique from general psychopathology effects. *Br. J. Psychiatry* **218**, 35–42 (2021).
11. Taylor, M. J. *et al.* Association of genetic risk factors for psychiatric disorders and traits of these disorders in a Swedish population twin sample. *JAMA Psychiatry* **76**, 280–289 (2019).
12. Bishop, D. V. M. & Hayiou-Thomas, M. E. Heritability of specific language impairment depends on diagnostic criteria. *Genes Brain Behav.* **7**, 365–372 (2008).
13. Cheesman, R. *et al.* Childhood behaviour problems show the greatest gap between DNA-based and twin heritability. *Transl. Psychiatry* **7**, 1–9 (2017).
14. DeThorne, L. S. *et al.* Children's history of speech-language difficulties: Genetic influences and associations with reading-related measures. (2006).
15. Hayiou-Thomas, M. E., Dale, P. S. & Plomin, R. The etiology of variation in language skills changes with development: a longitudinal twin study of language from 2 to 12 years. *Dev. Sci.* **15**, 233–249 (2012).
16. Hayiou-Thomas, M. E., Dale, P. S. & Plomin, R. Language impairment from 4 to 12 years: Prediction and etiology. *J. Speech Lang. Hear. Res.* **57**, 850–864 (2014).
17. Hohnen, B. & Stevenson, J. The structure of genetic influences on general cognitive, language, phonological, and reading abilities. *Dev. Psychol.* **35**, 590 (1999).
18. Tomblin, J. B. & Buckwalter, P. R. Heritability of poor language achievement among twins. *J. Speech Lang. Hear. Res.* **41**, 188–199 (1998).

19. Trzaskowski, M., Dale, P. S. & Plomin, R. No genetic influence for childhood behavior problems from DNA analysis. *J. Am. Acad. Child Adolesc. Psychiatry* **52**, 1048-1056. e3 (2013).
20. van Beijsterveldt, C. E. M., Felsenfeld, S. & Boomsma, D. I. Bivariate genetic analyses of stuttering and nonfluency in a large sample of 5-year-old twins. (2010).
21. Bishop, D. V. Motor immaturity and specific speech and language impairment: Evidence for a common genetic basis. *Am. J. Med. Genet.* **114**, 56–63 (2002).
22. Bishop, D. V. M. DeFries–Fulker analysis of twin data with skewed distributions: Cautions and recommendations from a study of children's use of verb inflections. *Behav. Genet.* **35**, 479–490 (2005).
23. Bishop, D. V., Adams, C. V. & Norbury, C. F. Distinct genetic influences on grammar and phonological short-term memory deficits: evidence from 6-year-old twins. *Genes Brain Behav.* **5**, 158–169 (2006).
24. Bishop, D. V., Laws, G., Adams, C. & Norbury, C. F. High heritability of speech and language impairments in 6-year-old twins demonstrated using parent and teacher report. *Behav. Genet.* **36**, 173–184 (2006).
25. Bishop, D. V., North, T. & Donlan, C. Nonword repetition as a behavioural marker for inherited language impairment: Evidence from a twin study. *J. Child Psychol. Psychiatry* **37**, 391–403 (1996).
26. Dale, P. S., Rice, M. L., Rimfeld, K. & Hayiou-Thomas, M. E. Grammar clinical marker yields substantial heritability for language impairments in 16-year-old twins. *J. Speech Lang. Hear. Res.* **61**, 66–78 (2018).
27. Dionne, G. *et al.* Associations between sleep-wake consolidation and language development in early childhood: a longitudinal twin study. *Sleep* **34**, 987–995 (2011).
28. Dworzynski, K., Remington, A., Rijsdijk, F., Howell, P. & Plomin, R. Genetic etiology in cases of recovered and persistent stuttering in an unselected, longitudinal sample of young twins. (2007).
29. Hoekstra, R. A., Bartels, M., Van Leeuwen, M. & Boomsma, D. I. Genetic architecture of verbal abilities in children and adolescents. *Dev. Sci.* **12**, 1041–1053 (2009).
30. Mimeau, C. *et al.* The genetic and environmental etiology of the association between vocabulary and syntax in first grade. *Lang. Learn. Dev.* **14**, 149–166 (2018).
31. Price, T. S., Dale, P. S. & Plomin, R. A longitudinal genetic analysis of low verbal and nonverbal cognitive abilities in early childhood. *Twin Res. Hum. Genet.* **7**, 139–148 (2004).
32. Tosto, M. G. *et al.* The genetic architecture of oral language, reading fluency, and reading comprehension: A twin study from 7 to 16 years. *Dev. Psychol.* **53**, 1115 (2017).
33. Trzaskowski, M. *et al.* DNA evidence for strong genome-wide pleiotropy of cognitive and learning abilities. *Behav. Genet.* **43**, 267–273 (2013).
34. Viding, E. *et al.* Genetic and environmental influence on language impairment in 4-year-old same-sex and opposite-sex twins. *J. Child Psychol. Psychiatry* **45**, 315–325 (2004).
35. Bailey, A. *et al.* Autism as a strongly genetic disorder: evidence from a British twin study. *Psychol. Med.* **25**, 63–77 (1995).

36. Deng, W. *et al.* The relationship among genetic heritability, environmental effects, and autism spectrum disorders: 37 pairs of ascertained twin study. *J. Child Neurol.* **30**, 1794–1799 (2015).
37. Dworzynski, K. *et al.* Developmental path between language and autistic-like impairments: A twin study. *Infant Child Dev. Int. J. Res. Pract.* **17**, 121–136 (2008).
38. Dworzynski, K., Happe, F., Bolton, P. & Ronald, A. Relationship Between Symptom Domains in Autism Spectrum Disorders: A Population Based Twin Study. *J. Autism Dev. Disord.* **39**, 1197–1210 (2009).
39. Frazier, T. W. *et al.* A twin study of heritable and shared environmental contributions to autism. *J. Autism Dev. Disord.* **44**, 2013–2025 (2014).
40. Hallett, V., Ronald, A. & Happé, F. Investigating the association between autistic-like and internalizing traits in a community-based twin sample. *J. Am. Acad. Child Adolesc. Psychiatry* **48**, 618–627 (2009).
41. Hoekstra, R., Bartels, M., Verweij, C. & Boomsma, D. Heritability of Autistic Traits in the General Population. *Arch. Pediatr. Adolesc. Med.* **161**, 372–377 (2007).
42. Jones, A. P. *et al.* Phenotypic and aetiological associations between psychopathic tendencies, autistic traits, and emotion attribution. *Crim. Justice Behav.* **36**, 1198–1212 (2009).
43. Lundstrom, S. *et al.* Autism Spectrum Disorders and Autisticlike Traits Similar Etiology in the Extreme End and the Normal Variation. *Arch. Gen. Psychiatry* **69**, 46–52 (2012).
44. Pinto, R., Rijsdijk, F., Ronald, A., Asherson, P. & Kuntsi, J. The genetic overlap of attention-deficit/hyperactivity disorder and autistic-like traits: an investigation of individual symptom scales and cognitive markers. *J. Abnorm. Child Psychol.* **44**, 335–345 (2016).
45. Polderman, T. J., Posthuma, D., De Sonneville, L. M., Verhulst, F. C. & Boomsma, D. I. Genetic analyses of teacher ratings of problem behavior in 5-year-old twins. *Twin Res. Hum. Genet.* **9**, 122–130 (2006).
46. Robinson, E. *et al.* Evidence That Autistic Traits Show the Same Etiology in the General Population and at the Quantitative Extremes (5%, 2.5%, and 1%). *Arch. Gen. Psychiatry* **68**, 1113–1121 (2011).
47. Robinson, E. B. *et al.* A multivariate twin study of autistic traits in 12-year-olds: testing the fractionable autism triad hypothesis. *Behav Genet* **42**, 245–255 (2012).
48. Ronald, A. *et al.* Genetic Heterogeneity Between the Three Components of the Autism Spectrum: A Twin Study. *J. Am. Acad. Child Adolesc. Psychiatry* **45**, 691–699 (2006).
49. Ronald, A., Happe, F., Price, T., Baron-Cohen, S. & Plomin, R. Phenotypic and Genetic Overlap Between Autistic Traits at the Extremes of the General Population. *J. Am. Acad. Child Adolesc. Psychiatry* **45**, 1206–1214 (2006).
50. Ronald, A., Larsson, H., Anckarsäter, H. & Lichtenstein, P. Symptoms of autism and ADHD: a Swedish twin study examining their overlap. *J. Abnorm. Psychol.* **123**, 440 (2014).
51. Ronald, A., Simonoff, E., Kuntsi, J., Asherson, P. & Plomin, R. Evidence for overlapping genetic influences on autistic and ADHD behaviours in a community twin sample. *J. Child Psychol. Psychiatry* **49**, 535–542 (2008).

52. Scherff, A. *et al.* What causes internalising traits and autistic traits to co-occur in adolescence? A community-based twin study. *J. Abnorm. Child Psychol.* **42**, 601–610 (2014).
53. Scourfield, J., Martin, N., Eley, T. C. & McGuffin, P. The Genetic Relationship Between Social Cognition and Conduct Problems. *Behav Genet* **34**, 377–383 (2004).
54. Taylor, M. J. *et al.* Examining the association between autistic traits and atypical sensory reactivity: A twin study. *J. Am. Acad. Child Adolesc. Psychiatry* **57**, 96–102 (2018).
55. Taylor, M. J. *et al.* Etiology of Autism Spectrum Disorders and Autistic Traits Over Time. *JAMA Psychiatry* **77**, 936–943 (2020).
56. Taylor, M. J., Charman, T. & Ronald, A. Where are the strongest associations between autistic traits and traits of ADHD? Evidence from a community-based twin study. *Eur. Child Adolesc. Psychiatry* **24**, 1129–1138 (2015).
57. Tick, B. *et al.* Autism Spectrum Disorders and other mental health problems: Exploring etiological overlaps and phenotypic causal associations. *J. Am. Acad. Child Adolesc. Psychiatry* **55**, 106-113. e4 (2016).
58. Towers, H. *et al.* Genetic and environmental influences on teacher ratings of the Child Behavior Checklist. *Int. J. Behav. Dev.* **24**, 373–381 (2000).
59. Yip, B. H. K. *et al.* Heritable variation, with little or no maternal effect, accounts for recurrence risk to autism spectrum disorder in Sweden. *Biol. Psychiatry* **83**, 589–597 (2018).
60. Hallmayer, J. *et al.* Genetic heritability and shared environmental factors among twin pairs with autism. *Arch. Gen. Psychiatry* **68**, 1095–1102 (2011).
61. Lundstrom, S. *et al.* Autistic-like traits and their association with mental health problems in two nationwide twin cohorts of children and adults. *Psychol Med* **41**, 2423–2433 (2011).
62. Taniai, H., Nishiyama, T., Miyachi, T., Imaeda, M. & Sumi, S. Genetic influences on the broad spectrum of autism: Study of proband-ascertained twins. *Am. J. Med. Genet. B Neuropsychiatr. Genet.* **147B**, 844–849 (2008).
63. Lundstrom, S. *et al.* Trajectories leading to autism spectrum disorders are affected by paternal age: findings from two nationally representative twin studies. *J. Child Psychol. Psychiatry* **51**, 850–856 (2010).
64. Colvert, E. *et al.* Heritability of autism spectrum disorder in a UK population-based twin sample. *JAMA Psychiatry* **72**, 415–423 (2015).
65. Ronald, A., Happé, F. & Plomin, R. The genetic relationship between individual differences in social and nonsocial behaviours characteristic of autism. *Dev. Sci.* **8**, 444–458 (2005).
66. Boomsma, D. I., Van Beijsterveldt, T. C., Odintsova, V. V., Neale, M. C. & Dolan, C. V. Genetically Informed Regression Analysis: Application to Aggression Prediction by Inattention and Hyperactivity in Children and Adults. *Behav. Genet.* 1–14 (2020).
67. Brikell, I. *et al.* Relative immaturity in childhood and attention-deficit/hyperactivity disorder symptoms from childhood to early adulthood: exploring genetic and environmental overlap across development. *J. Am. Acad. Child Adolesc. Psychiatry* **55**, 886–895 (2016).

68. Brooker, R. J. *et al.* Attentional Control Explains Covariation Between Symptoms of Attention-Deficit/Hyperactivity Disorder and Anxiety During Adolescence. *J. Res. Adolesc.* **30**, 126–141 (2020).
69. Burt, S. A., Krueger, R. F., McGue, M. & Iacono, W. G. Sources of covariation among attention-deficit/hyperactivity disorder, oppositional defiant disorder, and conduct disorder: the importance of shared environment. *J. Abnorm. Psychol.* **110**, 516 (2001).
70. Burt, S. A., Larsson, H., Lichtenstein, P. & Klump, K. L. Additional evidence against shared environmental contributions to attention-deficit/hyperactivity problems. *Behav. Genet.* **42**, 711–721 (2012).
71. Chang, Z., Lichtenstein, P. & Larsson, H. The effects of childhood ADHD symptoms on early-onset substance use: A Swedish twin study. *J. Abnorm. Child Psychol.* **40**, 425–435 (2012).
72. Chang, Z., Lichtenstein, P., Asherson, P. J. & Larsson, H. Developmental twin study of attention problems: high heritabilities throughout development. *JAMA Psychiatry* **70**, 311–318 (2013).
73. Chen, T.-J. *et al.* Genetic and environmental influences on the relationship between ADHD symptoms and internalizing problems: A Chinese twin study. *Am. J. Med. Genet. Part B Neuropsychiatr. Genet. Off. Publ. Int. Soc. Psychiatr. Genet.* **171**, 931–7 (2016).
74. Cheung, C. H., Fazier-Wood, A. C., Asherson, P., Rijsdijk, F. & Kuntsi, J. Shared cognitive impairments and aetiology in ADHD symptoms and reading difficulties. *PloS One* **9**, e98590 (2014).
75. Coolidge, F. L., Thede, L. L. & Young, S. E. Heritability and the comorbidity of attention deficit hyperactivity disorder with behavioral disorders and executive function deficits: A preliminary investigation. *Dev. Neuropsychol.* **17**, 273–287 (2000).
76. Curran, S. *et al.* CHIP: Defining a dimension of the vulnerability to attention deficit hyperactivity disorder (ADHD) using sibling and individual data of children in a community-based sample. *Am. J. Med. Genet. B Neuropsychiatr. Genet.* **119**, 86–97 (2003).
77. de Zeeuw, E. L., van Beijsterveldt, C. E., Lubke, G. H., Glasner, T. J. & Boomsma, D. I. Childhood ODD and ADHD behavior: The effect of classroom sharing, gender, teacher gender and their interactions. *Behav. Genet.* **45**, 394–408 (2015).
78. Derks, E. *et al.* Genetic and Environmental Influences on the Relation Between Attention Problems and Attention Deficit Hyperactivity Disorder. *Behav Genet* **38**, 11–23 (2008).
79. Derks, E. M., Dolan, C. V., Hudziak, J. J., Neale, M. C. & Boomsma, D. I. Assessment and etiology of attention deficit hyperactivity disorder and oppositional defiant disorder in boys and girls. *Behav. Genet.* **37**, 559–566 (2007).
80. Derks, E. M., Hudziak, J. J., Van Beijsterveldt, C. E. M., Dolan, C. V. & Boomsma, D. I. Genetic analyses of maternal and teacher ratings on attention problems in 7-year-old Dutch twins. *Behav. Genet.* **36**, 833–844 (2006).
81. Dick, D., Viken, R., Kaprio, J., Pulkkinen, L. & Rose, R. Understanding the Covariation Among Childhood Externalizing Symptoms: Genetic and Environmental Influences on Conduct Disorder, Attention Deficit Hyperactivity Disorder, and Oppositional Defiant Disorder Symptoms. *J Abnorm Child Psychol* **33**, 219–229 (2005).

82. Dolan, C. V., De Zeeuw, E. L., Zayats, T., Van Beijsterveldt, C. E. M. & Boomsma, D. I. The (broad-sense) genetic correlations among four measures of inattention and hyperactivity in 12 year olds. *Behav. Genet.* **50**, 273–288 (2020).
83. Ebejer, J. L. *et al.* Genetic and environmental influences on inattention, hyperactivity-impulsivity, and reading: Kindergarten to grade 2. *Sci. Stud. Read.* **14**, 293–316 (2010).
84. Ebejer, J. L. *et al.* Contrast effects and sex influence maternal and self-report dimensional measures of Attention-Deficit Hyperactivity Disorder. *Behav. Genet.* **45**, 35–50 (2015).
85. Edelbrock, C., Rende, R., Plomin, R. & Thompson, L. A. A twin study of competence and problem behavior in childhood and early adolescence. *J. Child Psychol. Psychiatry* **36**, 775–785 (1995).
86. Gould, K. L., Coventry, W. L., Olson, R. K. & Byrne, B. Gene-environment interactions in ADHD: the roles of SES and chaos. *J. Abnorm. Child Psychol.* **46**, 251–263 (2018).
87. Greven, C. U., Asherson, P., Rijsdijk, F. V. & Plomin, R. A longitudinal twin study on the association between inattentive and hyperactive-impulsive ADHD symptoms. *J. Abnorm. Child Psychol.* **39**, 623–632 (2011).
88. Greven, C. U., Harlaar, N., Dale, P. S. & Plomin, R. Genetic overlap between ADHD symptoms and reading is largely driven by inattentiveness rather than hyperactivity-impulsivity. *J. Can. Acad. Child Adolesc. Psychiatry* **20**, 6 (2011).
89. Greven, C. U., Kovas, Y., Willcutt, E. G., Petrill, S. A. & Plomin, R. Evidence for shared genetic risk between ADHD symptoms and reduced mathematics ability: a twin study. *J. Child Psychol. Psychiatry* **55**, 39–48 (2014).
90. Greven, C., Rijsdijk, F., Asherson, P. & Plomin, R. A longitudinal twin study on the association between ADHD symptoms and reading. *J Child Psychol Psychiatry Allied Discipl* **53**, 234–242 (2012).
91. Greven, C., Rijsdijk, F. & Plomin, R. A Twin Study of ADHD Symptoms in Early Adolescence: Hyperactivity-impulsivity and Inattentiveness Show Substantial Genetic Overlap but Also Genetic Specificity. *J Abnorm Child Psychol* **39**, 265–275 (2011).
92. Hay, D. A., Bennett, K. S., Levy, F., Sergeant, J. & Swanson, J. A twin study of attention-deficit/hyperactivity disorder dimensions rated by the strengths and weaknesses of ADHD-symptoms and normal-behavior (SWAN) scale. *Biol. Psychiatry* **61**, 700–705 (2007).
93. Heutink, P., Verhuls, F. C. & Boomsma, D. I. A longitudinal twin study on IQ, executive functioning, and attention problems during childhood and early adolescence. *Acta Neurol Belg* **106**, 191–207 (2006).
94. Hudziak, J. J., Derks, E. M., Althoff, R. R., Rettew, D. C. & Boomsma, D. I. The Genetic and Environmental Contributions to Attention Deficit Hyperactivity Disorder as Measured by the Conners' Rating Scales—Revised. *Am. J. Psychiatry* **162**, 1614–1620 (2005).
95. Hur, Y. M. Increasing Phenotypic and Genetic variations in Hyperactivity/Inattention Problems from Age 3 to 13 Years: A Cross-Sectional Twin Study. *Twin Res. Hum. Genet.* **17**, 545–552 (2014).
96. Jaffee, S. R., Hanscombe, K. B., Haworth, C. M., Davis, O. S. & Plomin, R. Chaotic homes and children's disruptive behavior: A longitudinal cross-lagged twin study. *Psychol. Sci.* **23**, 643–650 (2012).

97. Johnson, W., McGue, M. & Iacono, W. Disruptive Behavior and School Grades: Genetic and Environmental Relations in 11-Year-Olds. *J Educ Psychol* **97**, 391–405 (2005).
98. Kan, K.-J. *et al.* Genetic and environmental stability in attention problems across the lifespan: evidence from the Netherlands twin register. *J. Am. Acad. Child Adolesc. Psychiatry* **52**, 12–25 (2013).
99. Kan, K.-J., van Beijsterveldt, C. E., Bartels, M. & Boomsma, D. I. Assessing genetic influences on behavior: informant and context dependency as illustrated by the analysis of attention problems. *Behav. Genet.* **44**, 326–336 (2014).
100. Kuja-Halkola, R., Lichtenstein, P., D'Onofrio, B. & Larsson, H. Codevelopment of ADHD and externalizing behavior from childhood to adulthood. *J Child Psychol Psychiatry Allied Discipl* **56**, 640–647 (2015).
101. Kuntsi, J. & Stevenson, J. Psychological Mechanisms in Hyperactivity: II The Role of Genetic Factors. *J. Child Psychol. Psychiatry* **42**, 211–219 (2001).
102. Kuntsi, J. *et al.* The Separation of ADHD Inattention and Hyperactivity-Impulsivity Symptoms: Pathways from Genetic Effects to Cognitive Impairments and Symptoms. *J Abnorm Child Psychol* **42**, 127–136 (2014).
103. Kuntsi, J., Gayán, J. & Stevenson, J. Parents' and teachers' ratings of problem behaviours in children: Genetic and contrast effects. *Twin Res. Hum. Genet.* **3**, 251–258 (2000).
104. Kuntsi, J., Rijsdijk, F., Ronald, A., Asherson, P. & Plomin, R. Genetic influences on the stability of attention-deficit/hyperactivity disorder symptoms from early to middle childhood. *Biol. Psychiatry* **57**, 647–654 (2005).
105. Larsson, H., Anckarsater, H., Råstam, M., Chang, Z. & Lichtenstein, P. Childhood attention-deficit hyperactivity disorder as an extreme of a continuous trait: A quantitative genetic study of 8,500 twin pairs. *J. Child Psychol. Psychiatry* **53**, 73–80 (2012).
106. Larsson, H., Dilshad, R., Lichtenstein, P. & Barker, E. Developmental trajectories of DSM-IV symptoms of attention-deficit/hyperactivity disorder: genetic effects, family risk and associated psychopathology. *J Child Psychol Psychiatry Allied Discipl* **52**, 954–963 (2011).
107. Lemery-Chalfant, K., Doelger, L. & Goldsmith, H. H. Genetic relations between effortful and attentional control and symptoms of psychopathology in middle childhood. *Infant Child Dev.* **17**, 365–385 (2008).
108. Levy, F., Hay, D. A., McStephen, M., Wood, C. & Waldman, I. Attention-Deficit Hyperactivity Disorder: A Category or a Continuum? Genetic Analysis of a Large-Scale Twin Study. *J. Am. Acad. Child Adolesc. Psychiatry* **36**, 737–744 (1997).
109. Lewis, G. J. & Plomin, R. Heritable influences on behavioural problems from early childhood to mid-adolescence: evidence for genetic stability and innovation. *Psychol. Med.* **45**, 2171–2179 (2015).
110. Lewis, G., Haworth, C. & Plomin, R. Identical genetic influences underpin behavior problems in adolescence and basic traits of personality. *J Child Psychol Psychiatry Allied Discipl* **55**, 865–875 (2014).

111. Lifford, K. J., Harold, G. T. & Thapar, A. Parent–child hostility and child ADHD symptoms: A genetically sensitive and longitudinal analysis. *J. Child Psychol. Psychiatry* **50**, 1468–1476 (2009).
112. Little, C. W., Hart, S. A., Schatschneider, C. & Taylor, J. Examining associations among ADHD, homework behavior, and reading comprehension: A twin study. *J. Learn. Disabil.* **49**, 410–423 (2016).
113. LoParo, D. & Waldman, I. Twins' rearing environment similarity and childhood externalizing disorders: A test of the equal environments assumption. *Behav. Genet.* **44**, 606–613 (2014).
114. Martin, N. C., Piek, J. P. & Hay, D. DCD and ADHD: a genetic study of their shared aetiology. *Hum. Mov. Sci.* **25**, 110–124 (2006).
115. McLoughlin, G., Ronald, A., Kuntsi, J., Asherson, P. & Plomin, R. Genetic Support for the Dual Nature of Attention Deficit Hyperactivity Disorder: Substantial Genetic Overlap Between the Inattentive and Hyperactive–impulsive Components. *J Abnorm Child Psychol* **35**, 999–1008 (2007).
116. Merwood, A. *et al.* Different heritabilities but shared etiological influences for parent, teacher and self-ratings of ADHD symptoms: an adolescent twin study. *Psychol. Med.* **43**, (2013).
117. Michelini, G., Eley, T. C., Gregory, A. M. & McAdams, T. A. Aetiological overlap between anxiety and attention deficit hyperactivity symptom dimensions in adolescence. *J. Child Psychol. Psychiatry* **56**, 423–431 (2015).
118. Mikolajewski, A. J., Allan, N. P., Hart, S. A., Lonigan, C. J. & Taylor, J. Negative affect shares genetic and environmental influences with symptoms of childhood internalizing and externalizing disorders. *J. Abnorm. Child Psychol.* **41**, 411–423 (2013).
119. Molenaar, D., Middeldorp, C., van Beijsterveldt, T. & Boomsma, D. I. Analysis of behavioral and emotional problems in children highlights the role of genotype× environment interaction. *Child Dev.* **86**, 1999–2016 (2015).
120. Moruzzi, S., Rijsdijk, F. & Battaglia, M. A twin study of the relationships among inattention, hyperactivity/impulsivity and sluggish cognitive tempo problems. *J. Abnorm. Child Psychol.* **42**, 63–75 (2014).
121. Nikolas, M. A., Klump, K. L. & Burt, S. A. Parental involvement moderates etiological influences on attention deficit hyperactivity disorder behaviors in child twins. *Child Dev.* **86**, 224–240 (2015).
122. Niv, S., Tuvblad, C., Raine, A., Wang, P. & Baker, L. A. Heritability and longitudinal stability of impulsivity in adolescence. *Behav Genet* **42**, 378–392 (2012).
123. Paloyelis, Y., Rijsdijk, F., Wood, A. C., Asherson, P. & Kuntsi, J. The genetic association between ADHD symptoms and reading difficulties: the role of inattentiveness and IQ. *J. Abnorm. Child Psychol.* **38**, 1083–1095 (2010).
124. Peng, C.-Z. *et al.* Familial influences on the full range of variability in attention and activity levels during adolescence: A longitudinal twin study. *Dev. Psychopathol.* **28**, 517 (2016).

125. Pingault, J. B., Rijsdijk, F., Zheng, Y., Plomin, R. & Viding, E. Developmentally dynamic genome: Evidence of genetic influences on increases and decreases in conduct problems from early childhood to adolescence. *Sci. Rep.* **5**, 9 (2015).
126. Plourde, V. *et al.* Phenotypic and genetic associations between reading comprehension, decoding skills, and ADHD dimensions: evidence from two population-based studies. *J. Child Psychol. Psychiatry* **56**, 1074–1082 (2015).
127. Plourde, V., Boivin, M., Brendgen, M., Vitaro, F. & Dionne, G. Phenotypic and genetic associations between reading and attention-deficit/hyperactivity disorder dimensions in adolescence. *Dev. Psychopathol.* **29**, 1215–1226 (2017).
128. Polderman, T. J. *et al.* A genetic study on attention problems and academic skills: results of a longitudinal study in twins. *J. Can. Acad. Child Adolesc. PsychiatryJournal Académie Can. Psychiatr. Enfant Adolesc.* (2011).
129. Polderman, T. J., van Dongen, J. & Boomsma, D. I. The relation between ADHD symptoms and fine motor control: a genetic study. *Child Neuropsychol.* **17**, 138–150 (2011).
130. Price, T. *et al.* Continuity and Change in Preschool ADHD Symptoms: Longitudinal Genetic Analysis with Contrast Effects. *Behav Genet* **35**, 121–132 (2005).
131. Quinn, P. D. *et al.* Childhood attention-deficit/hyperactivity disorder symptoms and the development of adolescent alcohol problems: A prospective, population-based study of Swedish twins. *Am. J. Med. Genet. B Neuropsychiatr. Genet.* **171**, 958–970 (2016).
132. Rosenberg, J., Pennington, B. F., Willcutt, E. G. & Olson, R. K. Gene by environment interactions influencing reading disability and the inattentive symptom dimension of attention deficit/hyperactivity disorder. *J. Child Psychol. Psychiatry* **53**, 243–251 (2012).
133. Rydell, M., Taylor, M. & Larsson, H. Genetic and environmental contributions to the association between ADHD and affective problems in early childhood-A Swedish population-based twin study. *Am J Med Genet Part B Neuropsychiatr Genet* **174**, 538–546 (2017).
134. Saudino, K. J. & Plomin, R. Why are hyperactivity and academic achievement related? *Child Dev.* **78**, 972–986 (2007).
135. Saunders, M. C. *et al.* The associations between callous-unemotional traits and symptoms of conduct problems, hyperactivity and emotional problems: A study of adolescent twins screened for neurodevelopmental problems. *J. Abnorm. Child Psychol.* **47**, 447–457 (2019).
136. Siebelink, N. M. *et al.* Genetic and environmental aetiologies of associations between dispositional mindfulness and ADHD traits: a population-based twin study. *Eur. Child Adolesc. Psychiatry* **28**, 1241–1251 (2019).
137. Simonoff, E. *et al.* Genetic influences on childhood hyperactivity: Contrast effects imply parental rating bias, not sibling interaction. *Psychol. Med.* **28**, 825–837 (1998).
138. Stern, A. *et al.* Associations between ADHD and emotional problems from childhood to young adulthood: a longitudinal genetically sensitive study. *J Child Psychol Psychiatry Allied Discipl* **61**, 1234–1242 (2020).
139. Stevenson, J. Evidence for a genetic etiology in hyperactivity in children. *Behav Genet* **22**, 337–344 (1992).

140. Stevenson, J., Pennington, B. F., Gilger, J. W., DeFries, J. C. & Gillis, J. J. Hyperactivity and Spelling Disability: Testing for Shared Genetic Aetiology. *J. Child Psychol. Psychiatry* **34**, 1137–1152 (1993).
141. Taylor, J., Allan, N., Mikolajewski, A. & Hart, S. Common genetic and nonshared environmental factors contribute to the association between socioemotional dispositions and the externalizing factor in children. *J Child Psychol Psychiatry Allied Discipl* **54**, 67–76 (2013).
142. Thapar, A., Hervas, A. & McGuffin, P. Childhood hyperactivity scores are highly heritable and show sibling competition effects: Twin study evidence. *Behav Genet* **25**, 537–544 (1995).
143. Tuvblad, C., Zheng, M., Raine, A. & Baker, L. A Common Genetic Factor Explains the Covariation Among ADHD ODD and CD Symptoms in 9-10 Year Old Boys and Girls. *J Abnorm Child Psychol* **37**, 153–167 (2009).
144. Tye, C. *et al.* Shared genetic influences on ADHD symptoms and very low-frequency EEG activity: a twin study. *J Child Psychol Psychiatry Allied Discipl* **53**, 706–715 (2012).
145. Vendlinski, M. K. *et al.* Relative influence of genetics and shared environment on child mental health symptoms depends on comorbidity. *PloS One* **9**, e103080 (2014).
146. Waszczuk, M. A., Zavos, H. M. & Eley, T. C. Why do depression, conduct, and hyperactivity symptoms co-occur across adolescence? The role of stable and dynamic genetic and environmental influences. *Eur. Child Adolesc. Psychiatry* 1–13 (2020).
147. Willcutt, E. G. *et al.* Preschool twin study of the relation between attention-deficit/hyperactivity disorder and prereading skills. *Read. Writ.* **20**, 103–125 (2007).
148. Willcutt, E. G. *et al.* Etiology and neuropsychology of comorbidity between RD and ADHD: The case for multiple-deficit models. *Cortex* **46**, 1345–1361 (2010).
149. Wood, A. C., Rijsdijk, F., Asherson, P. & Kuntsi, J. Hyperactive-impulsive symptom scores and oppositional behaviours reflect alternate manifestations of a single liability. *Behav. Genet.* **39**, 447–460 (2009).
150. Wood, A. C., Rijsdijk, F., Asherson, P. & Kuntsi, J. Inferring causation from cross-sectional data: examination of the causal relationship between hyperactivity–impulsivity and novelty seeking. *Front. Genet.* **2**, 6 (2011).
151. Wood, A. C., Kuntsi, J., Asherson, P. & Saudino, K. J. Actigraph data are, reliable, with functional reliability increasing with aggregation. *Behav. Res. Methods* **40**, 873–878 (2008).
152. Zheng, Y., Pingault, J.-B., Unger, J. B. & Rijsdijk, F. Genetic and environmental influences on attention-deficit/hyperactivity disorder symptoms in Chinese adolescents: a longitudinal twin study. *Eur. Child Adolesc. Psychiatry* **29**, 205–216 (2020).
153. Zumberge, A., Baker, L. A. & Manis, F. R. Focus on words: a twin study of reading and inattention. *Behav. Genet.* **37**, 284–293 (2007).
154. Burt, S. A., McGUE, M., Krueger, R. F. & Iacono, W. G. Sources of covariation among the child-externalizing disorders: informant effects and the shared environment. *Psychol. Med.* **35**, 1133 (2005).

155. Chen, Q. *et al.* Shared familial risk factors between attention-deficit/hyperactivity disorder and overweight/obesity - a population-based familial coaggregation study in Sweden. *J Child Psychol Psychiatry Allied Discipl* **58**, 711–718 (2017).
156. Crosbie, J. *et al.* Response inhibition and ADHD traits: correlates and heritability in a community sample. *J. Abnorm. Child Psychol.* **41**, 497–507 (2013).
157. Eilertsen, E. M. *et al.* Development of ADHD symptoms in preschool children: Genetic and environmental contributions. *Dev. Psychopathol.* (2018).
158. Fedko, I. O. *et al.* Heritability of behavioral problems in 7-year olds based on shared and unique aspects of parental views. *Behav. Genet.* **47**, 152–163 (2017).
159. Haberstick, B. C. *et al.* Genetic and environmental contributions to retrospectively reported DSM-IV childhood attention deficit hyperactivity disorder. *Psychol. Med.* **38**, 1057–1066 (2008).
160. Martin, N. C., Levy, F., Pieka, J. & Hay, D. A. A genetic study of attention deficit hyperactivity disorder, conduct disorder, oppositional defiant disorder and reading disability: Aetiological overlaps and implications. *Int. J. Disabil. Dev. Educ.* **53**, 21–34 (2006).
161. Merwood, A., Asherson, P. & Larsson, H. Genetic associations between the ADHD symptom dimensions and Cloninger's temperament dimensions in adult twins. *Eur. Neuropsychopharmacol.* **23**, 416–425 (2013).
162. Mogensen, N., Larsson, H., Lundholm, C. & Almqvist, C. Association between childhood asthma and ADHD symptoms in adolescence–a prospective population-based twin study. *Allergy* **66**, 1224–1230 (2011).
163. Nadder, T., Silberg, J., Eaves, L., Maes, H. & Meyer, J. Genetic Effects on ADHD Symptomatology in 7- to 13-Year-Old Twins: Results from a Telephone Survey. *Behav Genet* **28**, 83–99 (1998).
164. Rhee, S. H., Waldman, I. D., Hay, D. A. & Levy, F. Sex differences in genetic and environmental influences on DSM–III–R attention-deficit/hyperactivity disorder. *J. Abnorm. Psychol.* **108**, 24 (1999).
165. Rimfeld, K. *et al.* Genetic correlates of psychological responses to the COVID-19 crisis in young adult twins in Great Britain. *Behav. Genet.* **51**, 110–124 (2021).
166. Singh, A. & Waldman, I. The Etiology of Associations Between Negative Emotionality and Childhood Externalizing Disorders. *J. Abnorm. Psychol.* **119**, 376–388 (2010).
167. Willcutt, E., Pennington, B. & DeFries, J. Etiology of Inattention and Hyperactivity/Impulsivity in a Community Sample of Twins with Learning Difficulties. *J Abnorm Child Psychol* **28**, 149–159 (2000).
168. Willcutt, E. G., Pennington, B. F., Olson, R. K. & DeFries, J. C. Understanding comorbidity: A twin study of reading disability and attention-deficit/hyperactivity disorder. *Am. J. Med. Genet. B Neuropsychiatr. Genet.* **144**, 709–714 (2007).
169. Merwood, A. *et al.* Genetic associations between the symptoms of attention-deficit/hyperactivity disorder and emotional lability in child and adolescent twins. *J. Am. Acad. Child Adolesc. Psychiatry* **53**, 209-220. e4 (2014).
170. Thapar, A., Harrington, R., Ross, K. & McGuffin, P. Does the Definition of ADHD Affect Heritability? *J. Am. Acad. Child Adolesc. Psychiatry* **39**, 1528–1536 (2000).

171. Ehringer, M. A., Rhee, S. H., Young, S., Corley, R. & Hewitt, J. K. Genetic and environmental contributions to common psychopathologies of childhood and adolescence: a study of twins and their siblings. *J. Abnorm. Child Psychol.* **34**, 1–17 (2006).
172. Smith, A. K. *et al.* The role of attention-deficit/hyperactivity disorder in the association between verbal ability and conduct disorder. *Front. Psychiatry* **2**, 3 (2011).
173. Thapar, A., Harrington, R. & McGuffin, P. Examining the comorbidity of ADHD-related behaviours and conduct problems using a twin study design. *Br J Psychiatry* **179**, 224–229 (2001).
174. Martin, N., Scourfield, J. & McGuffin, P. Observer effects and heritability of childhood attention-deficit hyperactivity disorder symptoms. *Br. J. Psychiatry* **180**, 260–265 (2002).
175. Alarcón, M., DeFries, J. C., Light, J. G. & Pennington, B. F. A twin study of mathematics disability. *J. Learn. Disabil.* **30**, 617–623 (1997).
176. Bishop, D. V. M. Genetic influences on language impairment and literacy problems in children: Same or different? *J. Child Psychol. Psychiatry* **42**, 189–198 (2001).
177. Davis, C. J. *et al.* Etiology of reading difficulties and rapid naming: the Colorado Twin Study of Reading Disability. *Behav. Genet.* **31**, 625–635 (2001).
178. Davis, O. S. P. *et al.* Generalist genes and the Internet generation: etiology of learning abilities by web testing at age 10. *Genes Brain Behav.* **7**, 455–462 (2008).
179. Davis, O. S. *et al.* The correlation between reading and mathematics ability at age twelve has a substantial genetic component. *Nat. Commun.* **5**, 1–6 (2014).
180. DeFries, J. C. & Alarcón, M. Genetics of specific reading disability. *Ment. Retard. Dev. Disabil. Res. Rev.* **2**, 39–47 (1996).
181. DeFries, J. C., Knopik, V. S. & Wadsworth, S. J. Colorado twin study of reading disability. *Read. Atten. Disord. Neurobiol. Correl.* 17–41 (1999).
182. Erbeli, F., Hart, S. A., Wagner, R. K. & Taylor, J. Examining the etiology of reading disability as conceptualized by the hybrid model. *Sci. Stud. Read.* **22**, 167–180 (2018).
183. Erbeli, F., Hart, S. A. & Taylor, J. Genetic and environmental influences on achievement outcomes based on family history of learning disabilities status. *J. Learn. Disabil.* **52**, 135–145 (2019).
184. Gayan, J. & Olson, R. K. Genetic and environmental influences on orthographic and phonological skills in children with reading disabilities. *Dev. Neuropsychol.* **20**, 483–507 (2001).
185. Harlaar, N., Kovas, Y., Dale, P. S., Petrill, S. A. & Plomin, R. Mathematics is differentially related to reading comprehension and word decoding: Evidence from a genetically sensitive design. *J. Educ. Psychol.* **104**, 622 (2012).
186. Harlaar, N., Trzaskowski, M., Dale, P. S. & Plomin, R. Word reading fluency: Role of genome-wide single-nucleotide polymorphisms in developmental stability and correlations with print exposure. *Child Dev.* **85**, 1190–1205 (2014).
187. Hart, S. A., Petrill, S. A., Thompson, L. A. & Plomin, R. The ABCs of math: A genetic analysis of mathematics and its links with reading ability and general cognitive ability. *J. Educ. Psychol.* **101**, 388 (2009).

188. Hensler, B., Schatschneider, C., Taylor, J. & Wagner, R. Behavioral Genetic Approach to the Study of Dyslexia. *J Dev Behav Pediatr* **31**, 525–532 (2010).
189. Kovas, Y. *et al.* The genetic and environmental origins of learning abilities and disabilities in the early school years. *Monogr. Soc. Res. Child Dev.* i–156 (2007).
190. Marlow, A. *et al.* Investigation of Quantitative Measures Related to Reading Disability in a Large Sample of Sib-Pairs from the UK. *Behav Genet* **31**, 219–230 (2001).
191. Newsome, J., Boisvert, D. & Wright, J. P. Genetic and environmental influences on the co-occurrence of early academic achievement and externalizing behavior. *J. Crim. Justice* **42**, 45–53 (2014).
192. Olson, R. K., Gillis, J. J., Rack, J. P., DeFries, J. C. & Fulker, D. W. Confirmatory factor analysis of word recognition and process measures in the Colorado Reading Project. *Read. Writ.* **3**, 235–248 (1991).
193. Petrill, S. A. *et al.* Longitudinal genetic analysis of early reading: the Western Reserve reading project. *Read. Writ.* **20**, 127–146 (2007).
194. Samuelsson, S. *et al.* Genetic and environmental influences on prereading skills and early reading and spelling development in the United States, Australia, and Scandinavia. *Read. Writ.* **20**, 51–75 (2007).
195. Tosto, M. G. *et al.* Why do we differ in number sense? Evidence from a genetically sensitive investigation. *Intelligence* **43**, 35–46 (2014).
196. Wadsworth, S. J., DeFries, J. C., Willcutt, E. G., Pennington, B. F. & Olson, R. K. The Colorado longitudinal twin study of reading difficulties and ADHD: Etiologies of comorbidity and stability. *Twin Res. Hum. Genet.* **18**, 755–761 (2015).
197. Wadsworth, S. J., DeFries, J. C., Willcutt, E. G., Pennington, B. F. & Olson, R. K. Genetic etiologies of comorbidity and stability for reading difficulties and ADHD: A replication study. *Twin Res. Hum. Genet.* **19**, 647–651 (2016).
198. Wadsworth, S. J., Olson, R. K. & DeFries, J. C. Differential genetic etiology of reading difficulties as a function of IQ: an update. *Behav. Genet.* **40**, 751–758 (2010).
199. Wadsworth, S. J., Olson, R. K., Pennington, B. F. & DeFries, J. C. Differential genetic etiology of reading disability as a function of IQ. *J. Learn. Disabil.* **33**, 192–199 (2000).
200. Willcutt, E. G. *et al.* Understanding comorbidity between specific learning disabilities. *New Dir. Child Adolesc. Dev.* **2019**, 91–109 (2019).
201. Willcutt, E. G., Pennington, B. F. & DeFries, J. C. Twin study of the etiology of comorbidity between reading disability and attention-deficit/hyperactivity disorder. *Am. J. Med. Genet.* **96**, 293–301 (2000).
202. Astrom, R. L., Wadsworth, S. J., Olson, R. K., Willcutt, E. G. & DeFries, J. C. DeFries–Fulker analysis of longitudinal reading performance data from twin pairs ascertained for reading difficulties and from their nontwin siblings. *Behav. Genet.* **41**, 660–667 (2011).
203. Betjemann, R. S. *et al.* Genetic covariation between brain volumes and IQ, reading performance, and processing speed. *Behav. Genet.* **40**, 135–145 (2010).
204. Bishop, D. V., Adams, C. V. & Norbury, C. F. Using nonword repetition to distinguish genetic and environmental influences on early literacy development: A study of 6-year-old twins. *Am. J. Med. Genet. B Neuropsychiatr. Genet.* **129**, 94–96 (2004).

205. Castles, A., Datta, H., Gayan, J. & Olson, R. K. Varieties of developmental reading disorder: Genetic and environmental influences. *J. Exp. Child Psychol.* **72**, 73–94 (1999).
206. Christopher, M. E. *et al.* The genetic and environmental etiologies of individual differences in early reading growth in Australia, the United States, and Scandinavia. *J. Exp. Child Psychol.* **115**, 453–467 (2013).
207. Daucourt, M. C., Haughbrook, R., Van Bergen, E. & Hart, S. A. The association of parent-reported executive functioning, reading, and math is explained by nature, not nurture. *Dev. Psychol.* (2020).
208. DeFries, J. C., Fulker, D. W. & LaBuda, M. C. Evidence for a genetic aetiology in reading disability of twins. *Nature* **329**, 537–539 (1987).
209. Erbeli, F., Hart, S. A. & Taylor, J. Longitudinal associations among reading-related skills and reading comprehension: A twin study. *Child Dev.* **89**, e480–e493 (2018).
210. Friend, A. *et al.* Heritability of high reading ability and its interaction with parental education. *Behav. Genet.* **39**, 427–436 (2009).
211. Friend, A., DeFries, J. C., Wadsworth, S. J. & Olson, R. K. Genetic and environmental influences on word recognition and spelling deficits as a function of age. *Behav. Genet.* **37**, 477–486 (2007).
212. Garon-Carrier, G. *et al.* Persistent genetic and family-wide environmental contributions to early number knowledge and later achievement in mathematics. *Psychol. Sci.* **28**, 1707–1718 (2017).
213. Gayán, J. & Olson, R. K. Genetic and environmental influences on individual differences in printed word recognition. *J. Exp. Child Psychol.* **84**, 97–123 (2003).
214. Gillis, J. J., DeFries, J. C. & Fulker, D. W. Confirmatory factor analysis of reading and mathematics performance: A twin study. *Acta Genet. Medicae Gemellol. Twin Res.* **41**, 287–300 (1992).
215. Grasby, K. L. & Coventry, W. L. Longitudinal stability and growth in literacy and numeracy in Australian school students. *Behav. Genet.* **46**, 649–664 (2016).
216. Harlaar, N., Dale, P. S. & Plomin, R. Reading exposure: A (largely) environmental risk factor with environmentally-mediated effects on reading performance in the primary school years. *J. Child Psychol. Psychiatry* **48**, 1192–1199 (2007).
217. Hart, S. A. *et al.* Exploring how nature and nurture affect the development of reading: an analysis of the Florida Twin Project on reading. *Dev. Psychol.* **49**, 1971 (2013).
218. Hawke, J. L., Stallings, M. C., Wadsworth, S. J. & DeFries, J. C. DeFries–Fulker and Pearson–Aitken model-fitting analyses of reading performance data from selected and unselected twin pairs. *Behav. Genet.* **38**, 101–107 (2008).
219. Knopik, V. S. *et al.* Differential genetic etiology of reading component processes as a function of IQ. *Behav. Genet.* **32**, 181–198 (2002).
220. Knopik, V. S., Alarcón, M. & DeFries, J. C. Comorbidity of mathematics and reading deficits: Evidence for a genetic etiology. *Behav. Genet.* **27**, 447–453 (1997).
221. Kovas, Y. *et al.* Literacy and numeracy are more heritable than intelligence in primary school. *Psychol. Sci.* **24**, 2048–2056 (2013).

222. Kovas, Y. *et al.* Overlap and specificity of genetic and environmental influences on mathematics and reading disability in 10-year-old twins. *J. Child Psychol. Psychiatry* **48**, 914–922 (2007).
223. Lazaroo, N. K. *et al.* Genetic structure of IQ, phonemic decoding skill, and academic achievement. *Front. Genet.* **10**, 195 (2019).
224. Logan, J. A. *et al.* Reading development in young children: Genetic and environmental influences. *Child Dev.* **84**, 2131–2144 (2013).
225. Malanchini, M. *et al.* Reading self-perceived ability, enjoyment and achievement: A genetically informative study of their reciprocal links over time. *Dev. Psychol.* **53**, 698 (2017).
226. Malanchini, M. *et al.* Genetic factors underlie the association between anxiety, attitudes and performance in mathematics. *Transl. Psychiatry* **10**, 1–11 (2020).
227. Malanchini, M., Engelhardt, L. E., Grotzinger, A. D., Harden, K. P. & Tucker-Drob, E. M. "Same but different": Associations between multiple aspects of self-regulation, cognition, and academic abilities. *J. Pers. Soc. Psychol.* **117**, 1164 (2019).
228. Oliver, B. R., Dale, P. S. & Plomin, R. Writing and reading skills as assessed by teachers in 7-year olds: A behavioral genetic approach. *Cogn. Dev.* **22**, 77–95 (2007).
229. Petrill, S. A. *et al.* Genetic and environmental influences on the growth of early reading skills. *J. Child Psychol. Psychiatry* **51**, 660–667 (2010).
230. Rimfeld, K. *et al.* The stability of educational achievement across school years is largely explained by genetic factors. *NPJ Sci. Learn.* **3**, 1–10 (2018).
231. Rimfeld, K. *et al.* Teacher assessments during compulsory education are as reliable, stable and heritable as standardized test scores. *J. Child Psychol. Psychiatry* **60**, 1278–1288 (2019).
232. Rimfeld, K., Ayorech, Z., Dale, P. S., Kovas, Y. & Plomin, R. Genetics affects choice of academic subjects as well as achievement. *Sci. Rep.* **6**, 1–9 (2016).
233. Rimfeld, K., Kovas, Y., Dale, P. S. & Plomin, R. Pleiotropy across academic subjects at the end of compulsory education. *Sci. Rep.* **5**, 1–12 (2015).
234. Shakeshaft, N. G. *et al.* Strong genetic influence on a UK nationwide test of educational achievement at the end of compulsory education at age 16. *PloS One* **8**, e80341 (2013).
235. Swagerman, S. *et al.* Genetic transmission of reading ability. *Brain Lang* **172**, 3–8 (2017).
236. Taylor, J. & Schatschneider, C. Genetic influence on literacy constructs in kindergarten and first grade: Evidence from a diverse twin sample. *Behav. Genet.* **40**, 591–602 (2010).
237. Taylor, J., Erbeli, F., Hart, S. A. & Johnson, W. Early classroom reading gains moderate shared environmental influences on reading comprehension in adolescence. *J. Child Psychol. Psychiatry* **61**, 689–698 (2020).
238. Tosto, M. G. *et al.* The nature of the association between number line and mathematical performance: An international twin study. *Br. J. Educ. Psychol.* **89**, 787–803 (2019).
239. Tosto, M. G., Malykh, S., Voronin, I., Plomin, R. & Kovas, Y. The etiology of individual differences in maths beyond IQ: insights from 12-year old twins. *Procedia-Soc. Behav. Sci.* **86**, 429–434 (2013).

240. Wadsworth, S. J., Olson, R. K., Willcutt, E. G. & DeFries, J. C. Multiple regression analysis of reading performance data from twin pairs with reading difficulties and nontwin siblings: The augmented model. *Twin Res. Hum. Genet.* **15**, 116–119 (2012).
241. Wong, S. W., Chow, B. W.-Y., Ho, C. S.-H., Waye, M. M. & Bishop, D. V. Genetic and environmental overlap between Chinese and English reading-related skills in Chinese children. *Dev. Psychol.* **50**, 2539 (2014).
242. Keenan, J. M., Betjemann, R. S., Wadsworth, S. J., DeFries, J. C. & Olson, R. K. Genetic and environmental influences on reading and listening comprehension. *J. Res. Read.* **29**, 75–91 (2006).
243. Mataix-Cols, D. *et al.* Familial Risks of Tourette Syndrome and Chronic Tic Disorders: A Population-Based Cohort Study. *JAMA Psychiatry* **72**, 787 (2015).
244. Fliers, E. *et al.* ADHD and poor motor performance from a family genetic perspective. *J. Am. Acad. Child Adolesc. Psychiatry* **48**, 25–34 (2009).
245. Taylor, M. J. *et al.* Developmental associations between traits of autism spectrum disorder and attention-deficit/hyperactivity disorder: A genetically-informative, longitudinal twin study. *Psychol. Med.* **43**, 1735–1746 (2013).
246. Light, J. G., Pennington, B. F., Gilger, J. W. & DeFries, J. C. Reading disability and hyperactivity disorder: Evidence for a common genetic etiology. *Dev. Neuropsychol.* **11**, 323–335 (1995).
247. Ooki, S. Genetic and environmental influences on stuttering and tics in Japanese twin children. *Twin Res. Hum. Genet. Off. J. Int. Soc. Twin Stud.* **8**, 69–75 (2005).
248. Hur, Y.-M. Genetic and environmental etiology of the relationship between childhood hyperactivity/inattention and conduct problems in a South Korean twin sample. *Twin Res. Hum. Genet.* **18**, 290–297 (2015).
249. O'Nions, E. *et al.* Examining the Genetic and Environmental Associations between Autistic Social and Communication Deficits and Psychopathic Callous-Unemotional Traits. *PloS One* **10**, 12 (2015).
250. Spinath, F. M., Price, T. S., Dale, P. S. & Plomin, R. The genetic and environmental origins of language disability and ability. *Child Dev.* **75**, 445–454 (2004).
251. Taylor, M. J. *et al.* Language and traits of autism spectrum conditions: Evidence of limited phenotypic and etiological overlap. *Am. J. Med. Genet. B Neuropsychiatr. Genet.* **165**, 587–595 (2014).
252. Constantino, J. N. & Todd, R. D. Autistic traits in the general population: a twin study. *Arch. Gen. Psychiatry* **60**, 524–530 (2003).
253. Hallett, V., Ronald, A., Rijsdijk, F. & Happé, F. Disentangling the associations between autistic-like and internalizing traits: a community based twin study. *J. Abnorm. Child Psychol.* **40**, 815–827 (2012).
254. Hoekstra, R., Happe, F., Baron-Cohen, S. & Ronald, A. Limited genetic covariance between autistic traits and intelligence: Findings from a longitudinal twin study+. *Am J Med Genet Part B Neuropsychiatr Genet* **153B**, 994–1007 (2010).
255. Holmboe, K. *et al.* Strong genetic influences on the stability of autistic traits in childhood. *J. Am. Acad. Child Adolesc. Psychiatry* **53**, 221–230 (2014).

256. Taylor, M. J., Gillberg, C., Lichtenstein, P. & Lundstrom, S. Etiological influences on the stability of autistic traits from childhood to early adulthood: evidence from a twin study. *Mol. Autism* **8**, 5 (2017).
257. Mazefsky, C. A., Goin-Kochel, R. P., Riley, B. P. & Maes, H. H. Genetic and environmental influences on symptom domains in twins and siblings with autism. *Res. Autism Spectr. Disord.* **2**, 320–331 (2008).
258. Cole, J., Ball, H., Martin, N., Scourfield, J. & McGuffin, P. Genetic Overlap Between Measures of Hyperactivity/Inattention and Mood in Children and Adolescents. *J. Am. Acad. Child Adolesc. Psychiatry* **48**, 1094–1101 (2009).
259. Constantino, J., Hudziak, J. & Todd, R. Deficits in Reciprocal Social Behavior in Male Twins: Evidence for a Genetically Independent Domain of Psychopathology. *J. Am. Acad. Child Adolesc. Psychiatry* **42**, 458–467 (2003).
260. Eaves, L. J. *et al.* Genetics and developmental psychopathology .2. The main effects of genes and environment on behavioral problems in the Virginia twin study of adolescent behavioral development. *J. Child Psychol. Psychiatry* **38**, 965–980 (1997).
261. Eaves, L. *et al.* Genetic and Environmental Causes of Covariation in Interview Assessments of Disruptive Behavior in Child and Adolescent Twins. *Behav Genet* **30**, 321–334 (2000).
262. Gregory, A. M., Eley, T. C., O'Connor, T. G. & Plomin, R. Etiologies of associations between childhood sleep and behavioral problems in a large twin sample. *J. Am. Acad. Child Adolesc. Psychiatry* **43**, 744–751 (2004).
263. Hudziak, J., Rudiger, L., Neale, M., Heath, A. & Todd, R. A Twin Study of Inattentive, Aggressive, and Anxious/Depressed Behaviors. *J. Am. Acad. Child Adolesc. Psychiatry* **39**, 469–476 (2000).
264. Kuo, P.-H., Lin, C. C., Yang, H.-J., Soong, W.-T. & Chen, W. J. A twin study of competence and behavioral/emotional problems among adolescents in Taiwan. *Behav. Genet.* **34**, 63–74 (2004).
265. Larsson, H., Lichtenstein, P. & Larsson, J.-O. Genetic contributions to the development of ADHD subtypes from childhood to adolescence. *J. Am. Acad. Child Adolesc. Psychiatry* **45**, 973–981 (2006).
266. Van Beijsterveldt, C. E. M., Verhulst, F. C., Molenaar, P. C. M. & Boomsma, D. I. The genetic basis of problem behavior in 5-year-old Dutch twin pairs. *Behav. Genet.* **34**, 229–242 (2004).
267. Vierikko, E., Pulkkinen, L., Kaprio, J. & Rose, R. J. Genetic and environmental influences on the relationship between aggression and hyperactivity-impulsivity as rated by teachers and parents. *Twin Res. Hum. Genet.* **7**, 261–274 (2004).
268. de Zeeuw, E. L., van Beijsterveldt, C. E. M., Ehli, E. A., de Geus, E. J. C. & Boomsma, D. I. Attention Deficit Hyperactivity Disorder Symptoms and Low Educational Achievement: Evidence Supporting A Causal Hypothesis. *Behav. Genet.* **47**, 278–289 (2017).
269. Do, E. K. *et al.* The role of genetic and environmental influences on the association between childhood ADHD symptoms and BMI. *Int. J. Obes.* **43**, 33–42 (2019).

270. Larsson, J.-O., Larsson, H. & Lichtenstein, P. Genetic and environmental contributions to stability and change of ADHD symptoms between 8 and 13 years of age: a longitudinal twin study. *J. Am. Acad. Child Adolesc. Psychiatry* **43**, 1267–1275 (2004).
271. Nadder, T. S., Rutter, M., Silberg, J. L., Maes, H. H. & Eaves, L. J. Genetic effects on the variation and covariation of attention deficit-hyperactivity disorder (ADHD) and oppositional-defiant disorder/conduct disorder (ODD/CD) symptomatologies across informant and occasion of measurement. *Psychol. Med.* **32**, 39–53 (2002).
272. Rietveld, M. J., Hudziak, J. J., Bartels, M., Van Beijsterveldt, C. E. M. & Boomsma, D. I. Heritability of attention problems in children: longitudinal results from a study of twins, age 3 to 12. *J. Child Psychol. Psychiatry* **45**, 577–588 (2004).
273. Saudino, K., Ronald, A. & Plomin, R. The Etiology of Behavior Problems in 7-Year-Old Twins: Substantial Genetic Influence and Negligible Shared Environmental Influence for Parent Ratings and Ratings by Same and Different Teachers. *J Abnorm Child Psychol* **33**, 113–130 (2005).
274. Silberg, J. *et al.* Genetic and environmental influences on the covariation between hyperactivity and conduct disturbance in juvenile twins. *J. Child Psychol. Psychiatry* **37**, 803–816 (1996).
275. Sherman, D. K., Iacono, W. G. & McGue, M. K. Attention-Deficit Hyperactivity Disorder Dimensions: A Twin Study of Inattention and Impulsivity-Hyperactivity. *J. Am. Acad. Child Adolesc. Psychiatry* **36**, 745–753 (1997).
276. Alarcón, M., DeFries, J. C. & Fulker, D. W. Etiology of individual differences in reading performance: A test of sex limitation. *Behav. Genet.* **25**, 17–23 (1995).
277. Bates, T. C. *et al.* Behaviour genetic analyses of reading and spelling: A component processes approach. *Aust. J. Psychol.* **56**, 115–126 (2004).
278. Harlaar, N., Spinath, F. M., Dale, P. S. & Plomin, R. Genetic influences on early word recognition abilities and disabilities: A study of 7-year-old twins. *J. Child Psychol. Psychiatry* **46**, 373–384 (2005).
279. Reynolds, C. A. *et al.* The genetics of children's oral reading performance. *J. Child Psychol. Psychiatry* **37**, 425–434 (1996).
280. Knopik, V., Heath, A., Bucholz, K., Madden, P. & Waldron, M. Genetic and environmental influences on externalizing behavior and alcohol problems in adolescence: A female twin study. *Pharmacol Biochem Behav* **93**, 313–321 (2009).
281. Neuman, R. J. *et al.* Latent class analysis of ADHD and comorbid symptoms in a population sample of adolescent female twins. *J. Child Psychol. Psychiatry* **42**, 933–942 (2001).
282. Verhoef, E., Shapland, C. Y., Fisher, S. E., Dale, P. S. & St Pourcain, B. The developmental origins of genetic factors influencing language and literacy: Associations with early-childhood vocabulary. *J. Child Psychol. Psychiatry* (2020).
283. Gandal, M. J. *et al.* Shared molecular neuropathology across major psychiatric disorders parallels polygenic overlap. *Science* **359**, 693–697 (2018).
284. Grove, J. *et al.* Identification of common genetic risk variants for autism spectrum disorder. *Nat. Genet.* **51**, 431–444 (2019).
285. Hill, W. D. *et al.* Age-Dependent Pleiotropy Between General Cognitive Function and Major Psychiatric Disorders. *Biol. Psychiatry* **80**, 266–273 (2016).

286. Lee, S. H. *et al.* Genetic relationship between five psychiatric disorders estimated from genome-wide SNPs. *Nat. Genet.* **45**, 984 (2013).
287. Serdarevic, F. *et al.* Polygenic risk scores for developmental disorders, neuromotor functioning during infancy, and autistic traits in childhood. *Biol. Psychiatry* **87**, 132–138 (2020).
288. Solberg, B. S. *et al.* Patterns of psychiatric comorbidity and genetic correlations provide new insights into differences between attention-deficit/hyperactivity disorder and autism spectrum disorder. *Biol. Psychiatry* **86**, 587–598 (2019).
289. St Pourcain, B. *et al.* Variability in the common genetic architecture of social-communication spectrum phenotypes during childhood and adolescence. *Mol. Autism* **5**, 1–12 (2014).
290. St Pourcain, B. *et al.* Developmental changes within the genetic architecture of social communication behavior: a multivariate study of genetic variance in unrelated individuals. *Biol. Psychiatry* **83**, 598–606 (2018).
291. St Pourcain, B. *et al.* ASD and schizophrenia show distinct developmental profiles in common genetic overlap with population-based social communication difficulties. *Mol. Psychiatry* **23**, 263–270 (2018).
292. Stergiakouli, E. *et al.* Shared genetic influences between dimensional ASD and ADHD symptoms during child and adolescent development. *Mol. Autism* **8**, 1–13 (2017).
293. Warrier, V. & Baron-Cohen, S. Genetic contribution to 'theory of mind'in adolescence. *Sci. Rep.* **8**, 1–9 (2018).
294. Autism Spectrum Disorders Working Group of The Psychiatric Genomics, C. Meta-analysis of GWAS of over 16,000 individuals with autism spectrum disorder highlights a novel locus at 10q24.32 and a significant overlap with schizophrenia. *Mol. Autism* **8**, 21 (2017).
295. Pettersson, E. *et al.* Genetic influences on eight psychiatric disorders based on family data of 4 408 646 full and half-siblings, and genetic data of 333 748 cases and controls. *Psychol. Med.* **49**, 1166–1173 (2019).
296. Artigas, M. S. *et al.* Attention-deficit/hyperactivity disorder and lifetime cannabis use: genetic overlap and causality. *Mol. Psychiatry* **25**, 2493–2503 (2020).
297. Demontis, D. *et al.* Discovery of the first genome-wide significant risk loci for attention deficit/hyperactivity disorder. *Nat. Genet.* **51**, 63–75 (2019).
298. Martin, J. *et al.* A genetic investigation of sex bias in the prevalence of attention-deficit/hyperactivity disorder. *Biol. Psychiatry* **83**, 1044–1053 (2018).
299. Micalizzi, L. *et al.* Single nucleotide polymorphism heritability and differential patterns of genetic overlap between inattention and four neurocognitive factors in youth. *Dev. Psychopathol.* **33**, 76–86 (2021).
300. Middeldorp, C. M. *et al.* A genome-wide association meta-analysis of attention-deficit/hyperactivity disorder symptoms in population-based pediatric cohorts. *J. Am. Acad. Child Adolesc. Psychiatry* **55**, 896-905. e6 (2016).
301. Pappa, I. *et al.* Single nucleotide polymorphism heritability of behavior problems in childhood: genome-wide complex trait analysis. *J. Am. Acad. Child Adolesc. Psychiatry* **54**, 737–744 (2015).

302. Rovira, P. *et al.* Shared genetic background between children and adults with attention deficit/hyperactivity disorder. *Neuropsychopharmacology* 1–10 (2020).
303. Gialluisi, A. *et al.* Genome-wide association study reveals new insights into the heritability and genetic correlates of developmental dyslexia. *Mol. Psychiatry* 1–14 (2020).
304. Martin, J. *et al.* Examining sex-differentiated genetic effects across neuropsychiatric and behavioral traits. *Biol. Psychiatry* (2021).
