# Supplementary material for: A meta-analysis of genetic effects associated with neurodevelopmental disorders and co-occurring conditions
Source: Nat Hum Behav. 2023 Feb 20;7(4):642–56. doi: 10.1038/s41562-023-01530-y (PMC10129867; doi:10.1038/s41562-023-01530-y)
Supplement: Supplementary file 2 — Reporting Summary [file 41562_2023_1530_MOESM2_ESM.pdf]

nature portfolio

Corresponding author(s): Agnieszka Gidziela & Margherita Malanchini

Last updated by author(s): Jan 12, 2023

Reporting Summary

Nature Portfolio wishes to improve the reproducibility of the work that we publish. This form provides structure for consistency and transparency in reporting. For further information on Nature Portfolio policies, see our Editorial Policies and the Editorial Policy Checklist.

Statistics

For all statistical analyses, confirm that the following items are present in the figure legend, table legend, main text, or Methods section.

n/a	Confirmed	
☐	☒	The exact sample size (n) for each experimental group/condition, given as a discrete number and unit of measurement
☐	☒	A statement on whether measurements were taken from distinct samples or whether the same sample was measured repeatedly
☐	☒	The statistical test(s) used AND whether they are one- or two-sided<br>*Only common tests should be described solely by name; describe more complex techniques in the Methods section.*
☐	☒	A description of all covariates tested
☐	☒	A description of any assumptions or corrections, such as tests of normality and adjustment for multiple comparisons
☐	☒	A full description of the statistical parameters including central tendency (e.g. means) or other basic estimates (e.g. regression coefficient) AND variation (e.g. standard deviation) or associated estimates of uncertainty (e.g. confidence intervals)
☐	☒	For null hypothesis testing, the test statistic (e.g. F, t, r) with confidence intervals, effect sizes, degrees of freedom and P value noted<br>*Give P values as exact values whenever suitable.*
☒	☐	For Bayesian analysis, information on the choice of priors and Markov chain Monte Carlo settings
☒	☐	For hierarchical and complex designs, identification of the appropriate level for tests and full reporting of outcomes
☐	☒	Estimates of effect sizes (e.g. Cohen's d, Pearson's r), indicating how they were calculated

Our web collection on statistics for biologists contains articles on many of the points above.

Software and code

Policy information about availability of computer code

Data collection	Publications were searched within the Web of Science, Ovid Medline and Ovid Embase databases. Selection of publications was conducted using the Covidence systematic review software, Veritas Health Innovation, Melbourne, Australia (https://www.covidence.org/). Covidence uses iterative product development processes, and therefore does not use version numbers or years and is a web-based collaboration software platform that streamlines the production of systematic and other literature reviews.
Data analysis	Thge data were analyzed and visualized using R version 4.0.4 (R Core Team, 2021) and the following packages: metafor (Viechtbauer & Viechtbauer, 2015), psychometric (Fletcher & Fletcher, 2013), Meta-Analysis with Mean Differences (MAd) (Field & Gillett,2014), meta (Balduzzi, Rücker & Schwarzer, 2019), dmetar (Harrer, Cuijpers, Furukawa & Ebert, 2019) and ggplot2 (Wickham, Chang & Wickham, 2016).<br><br>The code for all analyses is available at https://github.com/CoDEresearchlab/Meta_analysis_NDDs_DICCs<br><br>References:<br>Balduzzi, S., Rücker, G. & Schwarzer, G. How to perform a meta-analysis with R: a practical tutorial. Evid. Based Ment. Health 22, 153–160 (2019).<br>Field, A. P. & Gillett, R. How to do a meta-analysis. Br. J. Math. Stat. Psychol. 63, 665–694 (2010).<br>Fletcher, T. D. & Fletcher, M. T. D. Package ‘psychometric’. Recuperado Httpcran Rproject Orgwebpackagespsychometricpsychometric Pdfel 4, (2013).<br>Harrer, M., Cuijpers, P., Furukawa, T. & Ebert, D. D. dmetar: Companion R package for the guide’Doing meta-analysis in R’. R Package Version 00 9000, (2019).<br>R Core Team (2021). R: A language and environment for statistical computing. R Foundation for Statistical Computing, Vienna, Austria. URL https://www.R-project.org/.<br>Viechtbauer, W. & Viechtbauer, M. W. Package ‘metafor’. Compr. R Arch. Netw. Package ‘metafor’ Httpcran R-Proj. Orgwebpackagesmetaformetafor Pdf (2015).<br>Wickham, H., Chang, W., & Wickham, M. H. (2016). Package ‘ggplot2’. Create elegant data visualisations using the grammar of graphics.

Version, 2(1), 1-189

For manuscripts utilizing custom algorithms or software that are central to the research but not yet described in published literature, software must be made available to editors and reviewers. We strongly encourage code deposition in a community repository (e.g. GitHub). See the Nature Portfolio guidelines for submitting code & software for further information.

Data

Policy information about availability of data

All manuscripts must include a data availability statement. This statement should provide the following information, where applicable:

- Accession codes, unique identifiers, or web links for publicly available datasets
- A description of any restrictions on data availability
- For clinical datasets or third party data, please ensure that the statement adheres to our policy

The data that support the findings, including the master extraction tables, are available at https://github.com/CoDEresearchlab/Meta_analysis_NDDs_DICCs

Publicly available databases:
Web of science (https://www.webofknowledge.com)
Ovid Medline (https://ovidsp.ovid.com)
Ovid Embase (https://ovidsp.ovid.com)

Field-specific reporting

Please select the one below that is the best fit for your research. If you are not sure, read the appropriate sections before making your selection.

☐ Life sciences ☒ Behavioural & social sciences ☐ Ecological, evolutionary & environmental sciences

For a reference copy of the document with all sections, see nature.com/documents/nr-reporting-summary-flat.pdf

Behavioural & social sciences study design

All studies must disclose on these points even when the disclosure is negative.

Study description

Systematic review and multilevel, random-effects meta-analysis of quantitative behaviour genetics studies reporting on the heritability and environmental influences on neurodevelopmental disorders and/or genetic and environmental correlations between neurodevelopmental disorders and disruptive, impulse control and conduct disorders in childhood and adolescence.

Research sample

The studies included were published prior to 4th of May 2021. Studies were only included if 75% or more of the sample consisted of children and/or adolescents. Based on guidelines from the World Health Organization (WHO; https://www.who.int/health-topics/adolescent-health#tab=tab_1), we defined the period from childhood to end of adolescence as ranging from age 4, the earliest age for compulsory schooling, to age 24, the end of adolescence. Studies that had selected participants based on other diagnoses not related to neurodevelopmental disorders and/or disruptive, impulse control and conduct disorder categories or based extreme vulnerability environmental insult unrelated to disorders of interest, such as alcohol abuse, were not included.

Sampling strategy

Sample sizes were not predetermined. Estimates derived from included studies were weighted by their standard error during meta-analysis. As a result, studies using larger samples were given more power than studies using smaller samples.

Data collection

Searches were conducted across three platforms: Web of Science, Ovid Medline, and Ovid Embase and the outputs managed with the aid of Covidence (https://www.covidence.org/).

After the initial searches were conducted and duplicate studies removed, 8,087 studies met the criteria for the first stage of screening, which involved title and abstract scanning. All titles and abstracts were screened by two independent, blinded reviewers to ensure inter-rater agreement. Conflicts were resolved by a third independent reviewer. After this initial screening phase, 6,834 studies were excluded as deemed not relevant for the purpose of the current meta-analysis.

The title and abstract screening process resulted in a total of 1,253 potentially eligible studies. The full text of each study was screened by two independent, blinded reviewers. Reviewer discrepancies were identified and resolved by a third independent reviewer. This resulted in 289 eligible articles. In addition, during full text screening, relevant review articles, meta-analyses, editorials, and conference abstracts were flagged to aid the potential discovery of further relevant studies by either screening the References sections or contacting the authors of conference abstracts. Through this process 7 additional studies were identified, which resulted in a total of 296 studies included in the current meta-analysis.

Timing

Studies were identified during three searches: the primary search conducted on the 20th of January 2021, the secondary search conducted on the 15th of April 2021 and the additional search of other relevant meta-analyses and reviews finalized on the 4th of May 2021.

Data exclusions

We excluded multiple-generation family designs (e.g., children-of-twins and in-vitro fertilization) due to the potential confounding in the genetic and environmental estimates that could have resulted from including parental traits in the models decomposing the covariance between family members. Studies that had selected participants based on other diagnoses not related to neurodevelopmental or disruptive, impulse control and conduct disorder categories or based on extreme vulnerability or

	environmental insult unrelated to neurodevelopmental or disruptive, impulse control and conduct disorders, such as alcohol abuse, were also excluded.
Non-participation	This meta-analysis used estimates reported by published studies, for which the participation rate was not recorded.
Randomization	This study was descriptive in nature and no experimental manipulation was involved.

Reporting for specific materials, systems and methods

We require information from authors about some types of materials, experimental systems and methods used in many studies. Here, indicate whether each material, system or method listed is relevant to your study. If you are not sure if a list item applies to your research, read the appropriate section before selecting a response.

Materials & experimental systems

n/a	Involved in the study	
☒	☐	Antibodies
☒	☐	Eukaryotic cell lines
☒	☐	Palaeontology and archaeology
☒	☐	Animals and other organisms
☒	☐	Human research participants
☒	☐	Clinical data
☒	☐	Dual use research of concern

Methods

n/a	Involved in the study	
☒	☐	ChIP-seq
☒	☐	Flow cytometry
☒	☐	MRI-based neuroimaging
